# A novel approach to identify the mechanism of miR-145-5p toxicity to podocytes based on the essential genes targeting analysis

**DOI:** 10.1016/j.omtn.2021.09.005

**Published:** 2021-09-20

**Authors:** Sipan Zhang, Junnan Wu, Xiaodong Zhu, Hui Song, Lu Ren, Qiaoli Tang, Xiaodong Xu, Chunbei Liu, Jiong Zhang, Weixin Hu, Zhihong Liu, Shaolin Shi

**Affiliations:** 1 National Clinical Research Center of Kidney Diseases, Jinling Hospital, Nanjing University School of Medicine, Nanjing, Jiangsu 21002, China

**Keywords:** miR-145-5p, podocyte, essential genes, small GTPases, toxicity, Arhgap24, single-cell RNA-seq

## Abstract

MicroRNAs (miRNAs) are emerging as effective therapeutic agents. When testing whether miR-145-5p could alleviate kidney injury, we unexpectedly found that extracellular vesicles loaded with miR-145-5p induced proteinuria and podocyte foot process effacement in normal control mice. To explore the mechanism of miR-145-5p’s toxicity to podocytes, we hypothesized that miR-145-5p could enter podocytes and inhibit genes essential for podocytes. We demonstrated that systemically administered miRNA can enter podocytes. Next, we predicted 611 podocyte essential genes based on single-cell RNA sequencing (RNA-seq) and found that 32 of them are predicted to be targeted by miR-145-5p. Functional annotation of the 32 podocyte essential genes revealed small GTPase-mediated signal transduction as the top pathway. We experimentally validated that miR-145-5p targeted Arhgap24 and Srgap1, the essential regulators of the Rho family of small GTPases, increased the activity of Rac1 and Cdc42, and reduced RhoA activity, accompanied by cellular injury, in podocytes. These results explain how miR-145-5p has deleterious effect on podocytes. Most importantly, our study provides a novel approach to investigate how a miRNA affects a given cell type, allowing not only identification of the molecular mechanism underlying an observed side effect of a miRNA drug but also prediction of miRNA drug toxicity on various cell types.

## Graphical abstract

**Figure:**
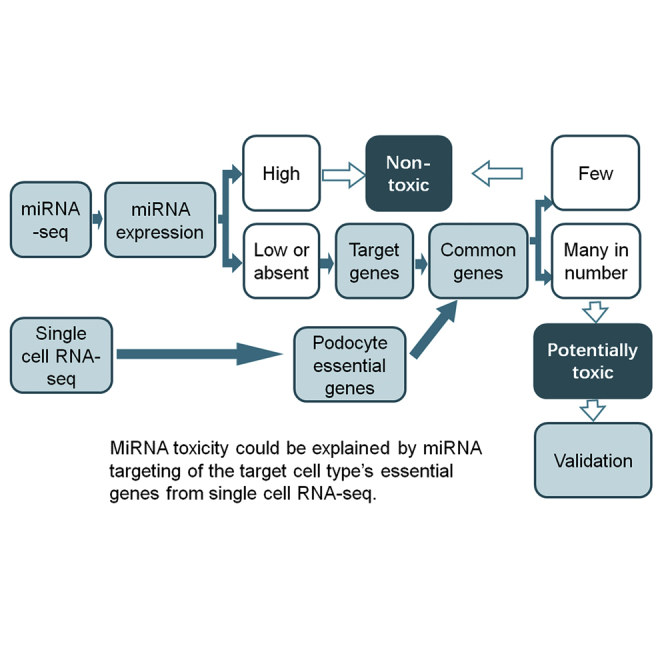

## Teaser

Zhang et al. describe an approach to predict miRNA toxicity to a given cell type based on miRNA targeting analysis of functionally essential genes of the cell type to determine whether any crucial pathway(s) is affected. This approach could facilitate miRNA drug development by eliminating potentially toxic miRNAs from the pipeline.

## Introduction

MicroRNAs (miRNAs) are posttranscriptional modulators of gene expression, and their aberrant expression is involved in the development of many diseases. Global downregulation of miRNAs is frequently observed in tumors,^1^ affecting many cellular processes, including cell cycle,^2^ proliferation,^3^ differentiation,^4^ and apoptosis,^5^ thereby facilitating tumorigenesis. Dysregulation of miRNAs is also involved in pathogenesis of non-tumoral diseases.^6^ Therefore, miRNA replacement therapy is promising. For example, let-7 was found to diminish the mass of murine lung cancer,^7^ and miR-26a adeno-assosiated virus suppressed tumorigenesis in mice.^8^ In addition, other miRNAs have also been tested for treatment of diseases (e.g., miR-34a,^9^ miR-16,^10^ miR-29,^11^ and miR-143/miR-145^12^^,^^13^). Therapeutic miRNAs are often loaded in liposomes,^14^ minicells,^10^ extracellular vehicles,^15^ or other vehicles^16^ and then systemically administered. Systemically administered vehicles and miRNAs can reach many normal tissues, including kidney. miRNA-based drugs eventually get to the kidney for clearance by excretion, making kidney cells more likely to receive the miRNAs. Thus, it is desirable to treat kidney diseases using miRNA-based drugs.

miR-145-5p dysregulation has been shown to be involved in kidney injury by mediating the effect of MALAT1^17^ and MEG3^18^ on kidney. Recently, we investigated whether systemically administered miR-145-5p could mitigate kidney injury in mouse models, and, unexpectedly, we found that treatment of normal control mice with extracellular vesicles (EVs) loaded with miR-145-5p resulted in albuminuria and podocyte foot process effacement. In the present study, we studied how the systemically administered miR-145-5p caused podocyte injury. We speculated that the circulating miR-145-5p in EVs can enter podocytes and inhibit the expression of target genes that are essential for podocytes, leading to podocyte injury and glomerular filtration barrier disruption. To prove this hypothesis, it is required to determine the essential genes of podocytes and then compare them with miR-145-5p's predicted targets in order to identify which podocyte essential genes are targeted by miR-145-5p.

Recently, we designed an approach to identify genes essential for mouse podocytes and other cell types.^19^ The rationale of the approach is that individual cells of the same type exhibit tremendous difference in gene expression, with correlation coefficients of 0.1–0.5, as shown by single-cell RNA sequencing (RNA-seq) studies,^20^, ^21^, ^22^ indicating that most genes are differentially expressed among individual cells of the same type, and the genes differentially expressed are dispensable, whereas those expressed commonly in all individual cells would be essential for the cell type. By this criterion, we identified candidate podocyte essential genes based on our mouse podocyte single-cell RNA-seq data and experimentally validated their essentiality for podocytes, demonstrating the feasibility of the approach.^19^ The availability of podocyte essential genes made it possible to test whether miR-145-5p could target podocyte essential genes and induce podocyte injury.

We then compared the predicted miR-145-5p targets with the 611 podocyte essential genes and identified 32 miR-145-5p targets. Gene ontology (GO) analysis of the predicted 32 podocyte essential genes revealed small GTPases as the top biological process affected by miR-145-5p. Optimal activity of small GTPases is known to be crucial for podocyte survival, structure, and function.^23^, ^24^, ^25^ We have proved that miR-145-5p alters small GTPase activity and induces podocyte injury. Our interpretation of the mechanism underlying miR-145-5p toxicity on podocytes provides a novel approach for understanding the mechanism underlying the side effects of miRNA-based drugs, as well as for prediction of toxicity of a miRNA drug.

## Results

### Systemically administered miR-145-5p induced podocyte injury in mice

**Figure 1:**
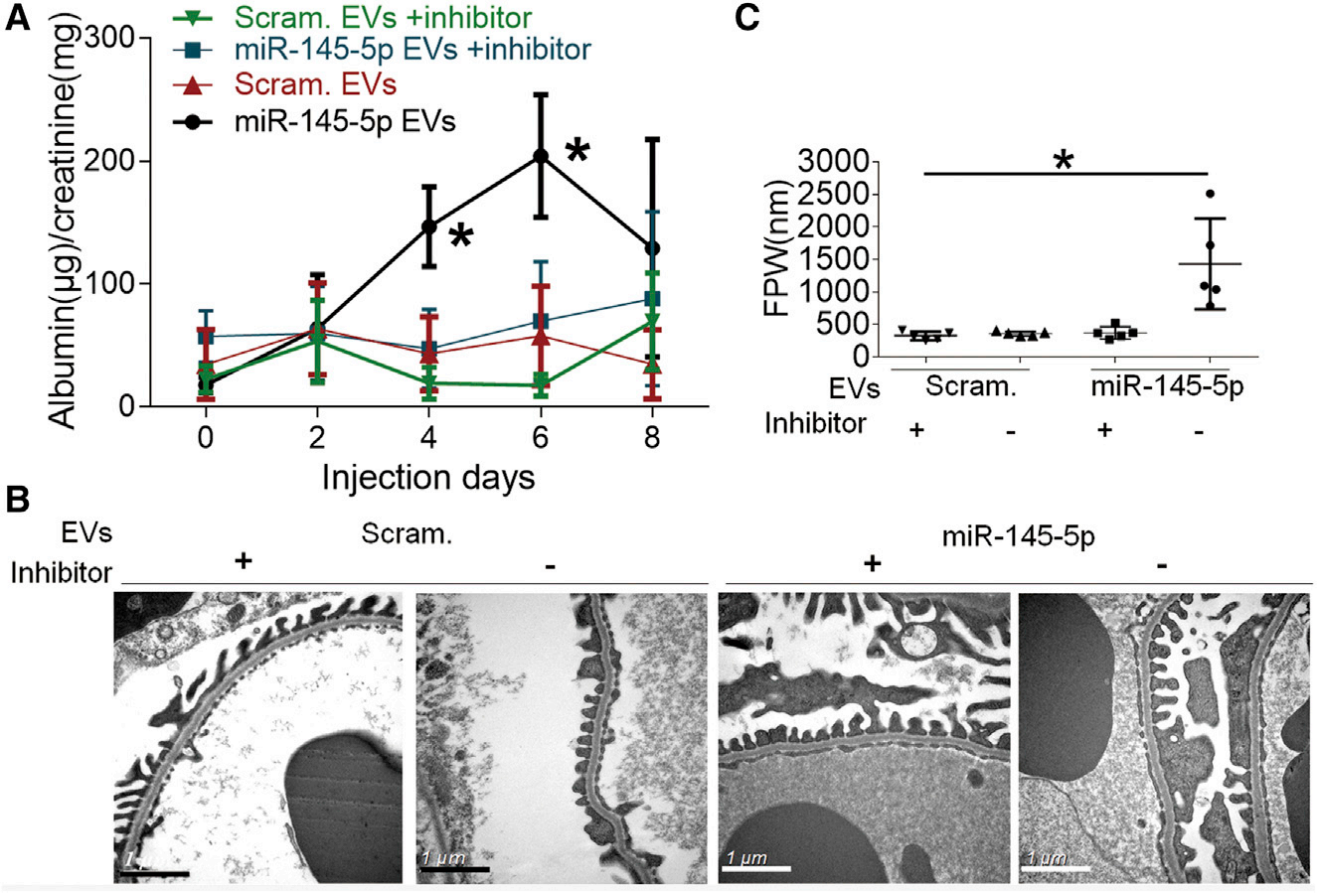
miR-145-5p induced podocyte injury in mice (A) Time course of the development of proteinuria urine albumin/creatinine ratio (ACR) in the mice treated with miR-145-5p EVs in the presence (miR-145-5p EVs +inhibitor) and absence (miR-145-5p EVs) of miR-145-5p inhibitor. miR-145-5p EV sample and inhibitor were administered on days 1, 2, 3, 4, 5, and 6. (B and C) Electronic microscopy (EM) examination revealed apparent foot process effacement of the podocytes in mice treated with miR-145-5p EVs, which was reversed by miR-145-5p inhibitor. ∗p < 0.05. Data are represented as mean ± SD.

miR-145-5p has been reported to be dysregulated and involved in kidney injuries.^17^^,^^18^ To determine whether supplement of miR-145-5p could alleviate kidney injury in mouse models, we purified the EVs from miR-145-5p transfected Jurkat or HEK293 cells and obtained the miR-145-5p-enriched EVs (Figure S1). We first tested the miR-145-5p EV samples for toxicity or side effects on healthy control mice by administering miR-145-5p EVs intravenously every other day for a total of 3 times. Unexpectedly, we found that miR-145-5p EVs induced albuminuria in the mice (Figure S2). To further confirm the albuminuria-inducing effect of miR-145-5p, we repeated the experiment with miR-145-5p EV injection once a day for a total of 6 days. To support that the toxicity of miR-145-5p EVs was miR-145-5p specific, we included a group of mice that were simultaneously injected with miR-145-5p inhibitor using TransIT-EE Delivery Solution, expecting an alleviated albuminuria in the mice. As shown in Figure 1, miR-145-5p EVs induced albuminuria and podocyte foot process effacement, and this effect was abolished by miR-145-5p inhibitor.

### miRNA loaded in extracellular vesicles can reach podocytes after being systemically administered

**Figure 2:**
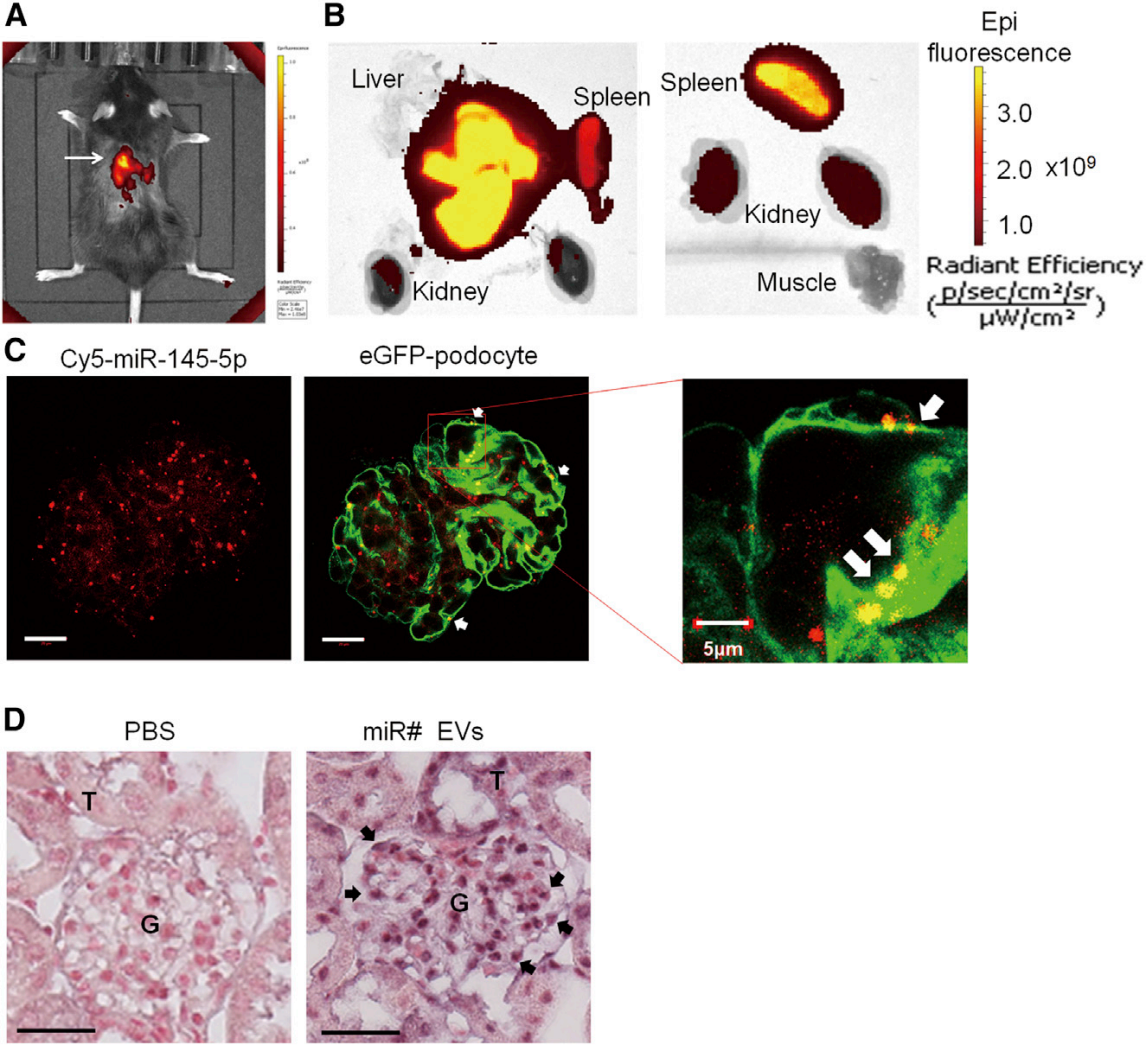
Tissue distribution of injected EVs and the miRNA in a mouse (A) Fluorescence imaging of the mouse injected with DiR-labeled EVs through tail vein. The image was taken 30 min after the injection, showing predominant fluorescence signal in liver as well as other tissues. (B) Fluorescence imaging of individual organs from the same mouse, showing that EVs were accumulated in liver, kidney, and spleen but not muscles. (C) Confocal imaging of Cy5-miR-145-5p in an isolated glomerulus 48 h after the Cy5-miR-145-5p-loaded EVs were injected via tail vein to a *Npsh2*-cre/eGFP transgenic mouse whose podocytes express eGFP. Arrows: Cy5-miR-145-5p-loaded EVs accumulated in podocytes. Scale bar: 20 μm. (D) *In situ* hybridization of artificial miR# in kidney after the miR#-containing EVs were injected via tail vein to a mouse. It was clearly shown that miR# was delivered to all glomerular cells, including podocytes that are localized at periphery of each glomerular tuft. Arrows: miR#-loaded EVs accumulated in podocytes. G, glomerulus; T, tubule. Scale bar: 20 μm.

To determine whether EVs and the enclosed miRNA can be delivered to podocytes after being administered systemically, we first performed live fluorescence imaging of the distribution of EVs labeled by DiR (a near-infrared dye) in mice after injection via tail vein. The fluorescence signal was predominantly present in liver (Figures 2A and 2B); meanwhile, abundant fluorescence was also observed in spleen and kidney, but not muscle (Figure 2B), which was in accordance with former publications.^26^^,^^27^

We next examined whether miRNA mimic labeled with Cy5 in the EVs was successfully delivered to podocytes and found that Cy5-miR-145-5p was clearly present in all glomerular cells, including podocytes that were located at the periphery of each glomerular tuft (Figure 2C). This observation was in accordance with results of *in situ* hybridization of an artificial miRNA mimic (miR#) (Figure 2D). No miR# was detected in mice injected with PBS, while in the mice injected with miR# EVs, all glomerular cells, including podocytes, were positive for miR# staining. These results were consistent with studies showing that intravenously administered EVs were readily delivered to extensive kidney tissues, including glomerular podocytes.^28^^,^^29^

### Systemically administered miR-145-5p agomir entered podocytes and caused injury

We next tested the toxicity of miR-145-5p in the form of agomir, which is commonly used for miR-based drug delivery via systemic administration.^30^ We first verified that Cy5-agomir injected via tail vein was able to reach podocytes (Figure S3A). Then, miR-145-5p and scramble agomirs were injected every other day for a total of 6 times, and proteinuria developed in the miR-145-5p agomir-treated mice but not the scramble-treated mice (Figure S3B). EM examination consistently showed podocyte foot process effacement in the miR-145-5p- but not scramble-treated mice (Figure S3C). Additionally, we injected miR-145-5p agomir locally to mouse kidney, and 3 days after injection, marked foot process effacement was observed in the kidney injected with miR-145-5p agomir but not scramble (Figure S3D).

### miR-145-5p induced injury in cultured podocytes

**Figure 3:**
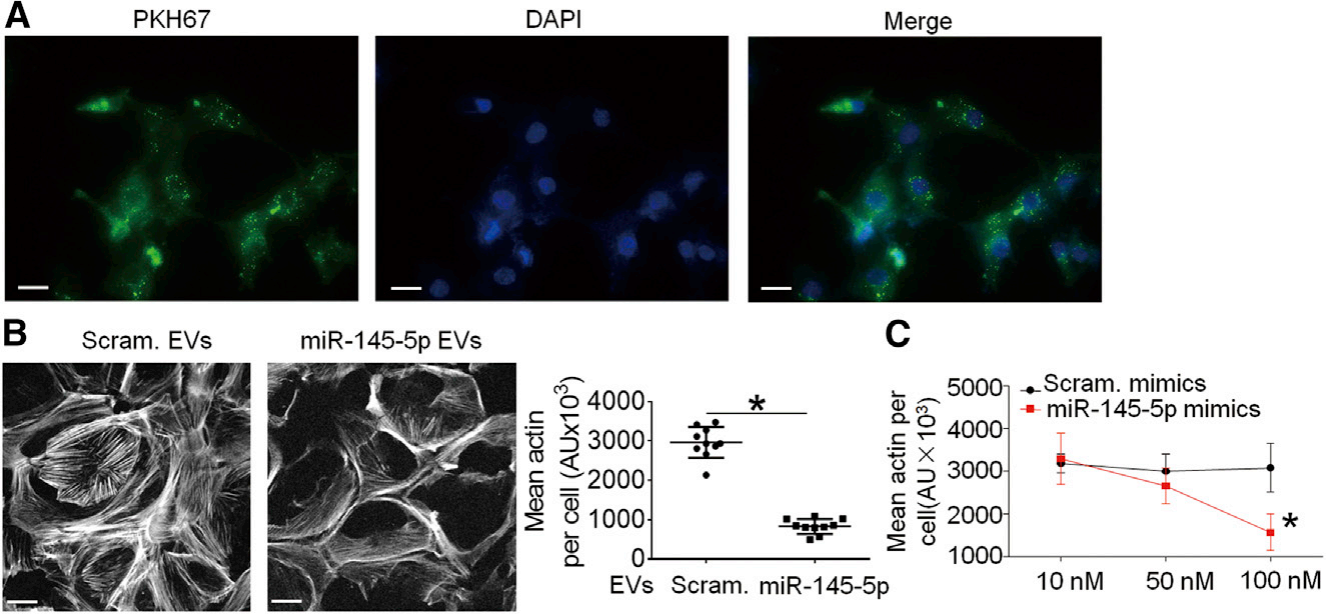
Toxicity of miR-145-5p on podocytes in culture (A) Fluorescent EVs were incubated with podocytes in culture, and the fluorescence was observed in the cells after 12 h. Scale bar: 100 μm. (B) Phalloidin staining of cultured podocytes treated with miR-145-5p EVs, showing a reduction of F-actin stress fibers in the cells. Scale bar: 50 μm. (C) Quantifications of F-actin stress fibers in cultured podocytes after transfection of miR-145-5p mimic and scramble control, which shows that miR-145-5p mimic induced a reduction of F-actin stress fibers. The results represented data from three independent experiments. ∗p < 0.05, statistically significant. Data are represented as mean ± SD.

We tested whether miR-145-5p could cause injury in immortalized podocyte cell line in culture, which is a commonly used model for podocyte research. We added miR-145-5p EVs, which were labeled with PKH67, to podocytes in culture and observed fluorescence in the cells after 12 h (Figure 3A). We then quantified the reduction of podocyte F-actin stress fibers, because loss of F-actin stress fibers is characteristic of podocyte injury.^19^ The result showed that F-actin stress fibers were greatly reduced in the miR-145-5p EV-treated podocytes (Figure 3B). We also used miR-145-5p mimic to transfect the podocytes and observed a similar reduction of F-actin stress fibers in the cells (Figure 3C). The consistent toxic effect of miR-145-5p on the podocytes in culture with those in mice suggests that the podocyte cell line is a good model for exploring how miR-145-5p induces podocyte injury.

### Identification of 611 podocyte essential genes by single-cell RNA-seq

**Figure 4:**
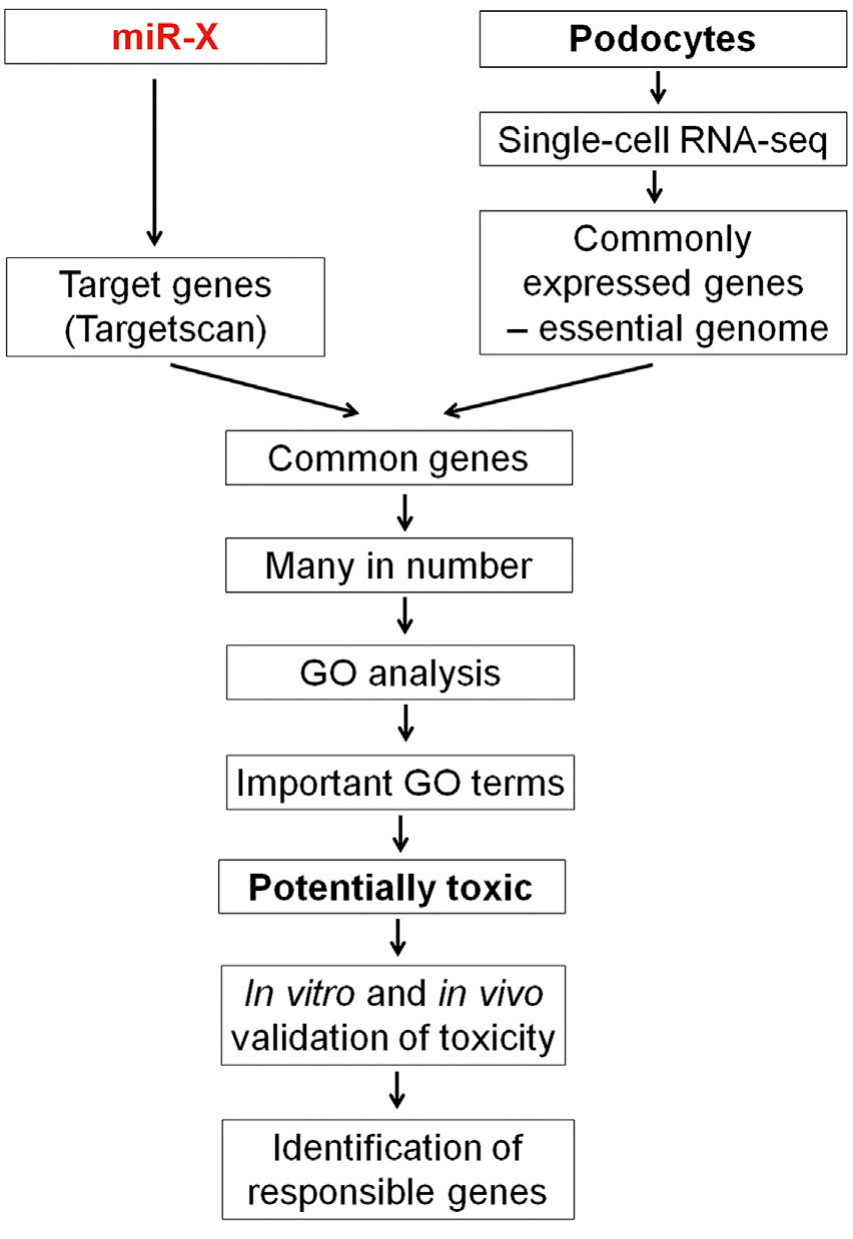
Schematic diagram of the approach for prediction of the toxicity of a miRNA based on its targeting of the essential genes of a cell type

We speculated that miR-145-5p is toxic to podocytes because it is not normally expressed in podocytes, and exogenous miR-145-5p can effectively target genes essential for podocytes, as shown in the schematic diagram (Figure 4). We previously performed single-cell RNA-seq of mouse podocytes and found enormous heterogeneity of gene expression among individual podocytes. By using the concept that genes commonly expressed in all individual podocytes are likely podocyte essential genes, we predicted and validated 335 podocyte essential genes when expression cutoff was set as >0.5 Reads Per Kilobase per Million mapped reads (RPKM).^19^ According to the estimation that there are approximately 2,000 genes that are essential for a cell type,^31^, ^32^, ^33^ we lowered the expression cutoff to >0.1 RPKM for the present study, resulting in 611 genes that are predicted to be podocyte essential genes (Table S1). GO analysis of the predicted 611 essential genes revealed housekeeping and other processes known to be essential for podocytes (e.g., actin cytoskeleton organization [Figure S4]), supporting the reliability of our prediction approach.

### Identification of podocyte essential genes targeted by the miR-145-5p

To determine how many of the podocyte essential genes can be targeted by miR-145-5p, we searched Targetscan and retrieved the top 500 predicted target genes of miR-145-5p. Comparison of miR-145-5p’s target genes with the predicted 611 podocyte essential genes revealed 32 podocyte essential genes that may be targeted by miR-145-5p (Table 1).

**Table 1:** Predicted targets of miR-145-5p among the 611 podocyte essential genes

Symbol	Entrez gene name	Average expression in single podocyte (RPKM)	Cumulative weighted context++ score
ACTB	actin, beta	13,889.86	−0.43
GNB1	guanine nucleotide-binding protein (G protein), beta polypeptide 1	2,439.721	−0.15
ST13	suppression of tumorigenicity 13	2,106.819	−0.04
DPYSL2	dihydropyrimidinase-like 2	1,419.238	−0.05
EIF4A2	eukaryotic translation initiation factor 4A2	1,082.761	−0.14
Podxl	podocalyxin-like	1,050.493	−0.18
SET	SET nuclear proto-oncogene	951.0317	−0.11
QKI	QKI, KH domain containing, RNA binding	776.3372	−0.12
TMOD3	tropomodulin 3 (ubiquitous)	531.727	−0.24
SKP1	S-phase kinase-associated protein 1	503.8126	−0.08
ARHGAP24	Rho GTPase-activating protein 24	452.6906	−0.23
MPP5	membrane protein, palmitoylated 5 (MAGUK p55 subfamily member 5)	270.9088	−0.35
NRAS	neuroblastoma RAS viral (v-ras) oncogene homolog	249.8165	−0.15
ACSL4	acyl-CoA synthetase long-chain family member 4	246.0129	−0.09
EPB41L5	erythrocyte membrane protein band 4.1 like 5	214.3441	−0.24
ARHGAP28	Rho GTPase-activating protein 28	199.0145	−0.07
SRGAP1	SLIT-ROBO Rho GTPase-activating protein 1	172.8949	−0.31
CORO2B	coronin, actin-binding protein, 2B	172.8764	−0.19
DENND5B	DENN/MADD domain containing 5B	152.5768	−0.12
SPOP	speckle-type POZ protein	146.9724	−0.43
OGT	O-linked N-acetylglucosamine (GlcNAc) transferase	137.9184	−0.05
PLCE1	phospholipase C, epsilon 1	128.7123	−0.29
MAGI2	membrane-associated guanylate kinase, WW and PDZ domain containing 2	120.5446	−0.33
PDCD4	programmed cell death 4 (neoplastic transformation inhibitor)	109.5968	−0.18
NFIA	nuclear factor I/A	97.51652	−0.1
Dst	dystonin	97.29717	−0.33
NFE2L1	nuclear factor, erythroid 2-like 1	56.95308	−0.17
UBN2	ubinuclein 2	44.89126	−0.1
PURA	purine-rich element binding protein A	35.38958	−0.12
SIK2	salt-inducible kinase 2	32.43537	−0.12
ERMP1	endoplasmic reticulum metallopeptidase 1	12.50254	−0.1
HELLS	helicase, lymphoid-specific	1.301073	−0.2

### miR-145-5p is predicted to target Rho family of small GTPases in podocytes

**Figure 5:**
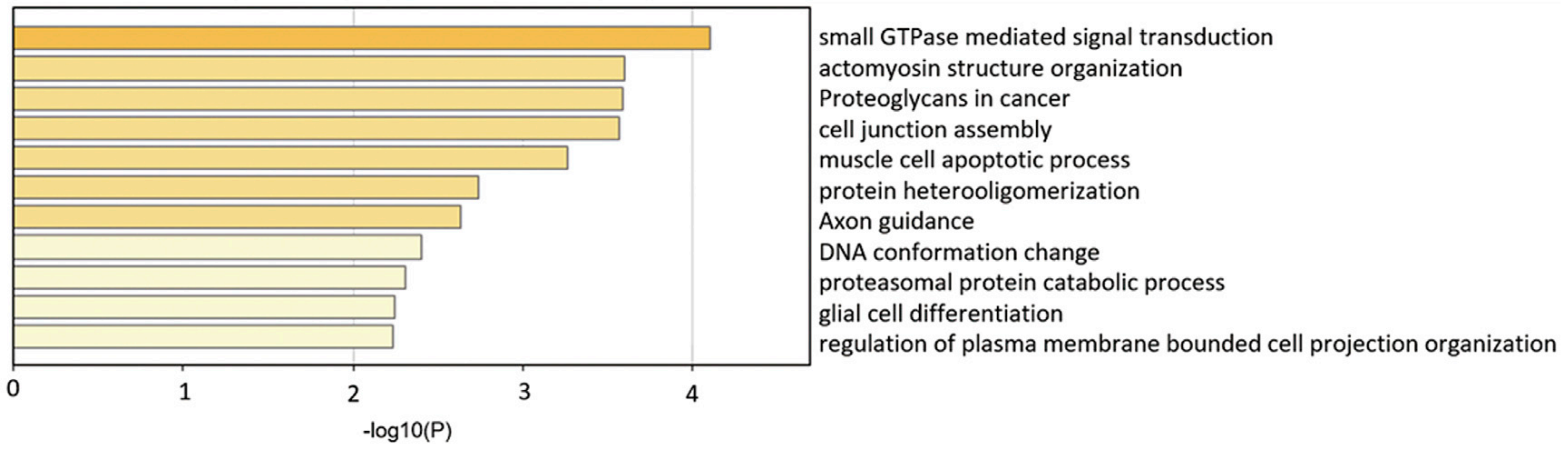
GO analysis of the 32 podocyte essential genes of miR-145-5p potential target

To explore the mechanism of miR-145-5p toxicity on podocytes, we performed GO functional annotation of the 32 podocyte essential genes that are miR-145-5p potential targets (Table 1) and found small GTPase-mediated signal transduction was at the top of the functions (Figure 5; Table S2). The members of Rho family of small GTPases (RhoA, Rac1, and Cdc42) have been best described and shown to be crucial for many cellular processes in podocytes. Aberrant activity of RhoA, Rac1, and Cdc42 has been shown to cause podocyte injury.^23^^,^^24^^,^^34^

### miR-145-5p inhibited Arhgap24 and Srgap1, altering the activity of Rho family of small GTPases in podocytes

**Figure 6:**
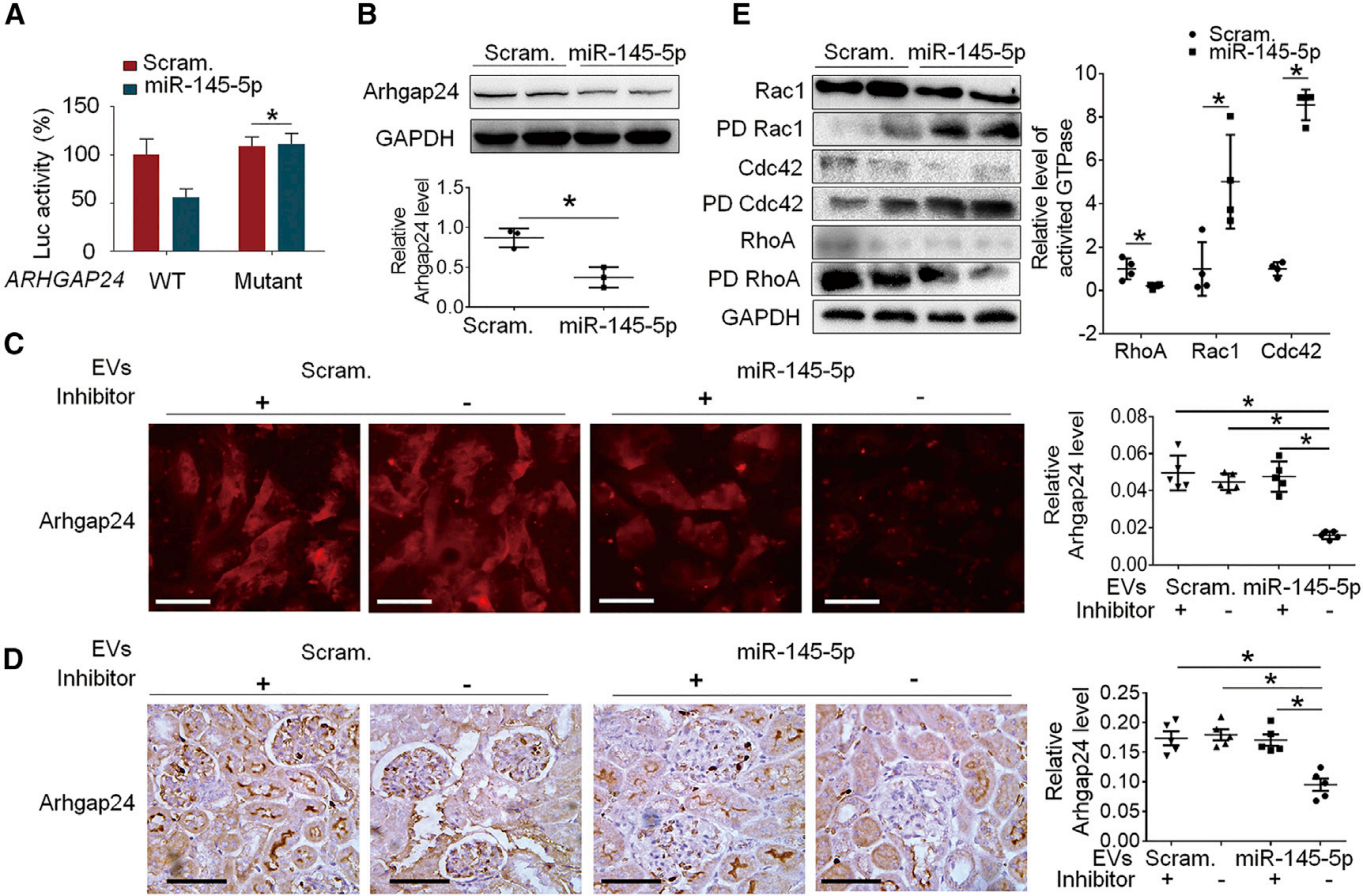
miR-145-5p targeted Arhgap24 and increased activity of Rac1 and Cdc42 in podocytes (A) Luciferase reporter assay: the reporter constructs contained Arhgap24 3′ UTRs with or without mutations in the miR-145-5p binding site downstream of Luc open reading frame (ORF) were prepared and were co-transfected with miR-145-5p mimic into cultured podocytes. (B) Immunoblotting of the proteins of Arhgap24 in the cultured podocytes after transfection with miR-145-5p mimic and scramble control, showing that the Arhgap24 protein was significantly downregulated by miR-145-5p. (C) Immunofluorescence staining of cultured podocytes showed downregulation of Arhgap24 protein by the miR-145-5p in EVs, and the effect of miR-145-5p EVs was abolished by the inhibitor. Scale bar: 100 μm. (D) Immunohistochemical staining of the Arhgap24 proteins in the glomeruli of mice treated as indicated. Scale bar: 40 μm. ∗p < 0.05, statistically significant. (E) miR-145-5p upregulated Rac1 and Cdc42 activities but reduced RhoA activity as shown by immunoblotting of the total and active form (PD, pulldown) of the proteins in cultured podocytes. ∗p < 0.05, statistically significant. Data are represented as mean ± SD.

miR-145-5p was predicted to target three GTPase-activating proteins, Arhgap24, Arhgap28, and Srgap1, which are associated with the small GTPase pathway (Table 2). Since loss of Arhgap24 is known to cause podocyte injury,^35^ we examined whether Arhgap24 is truly a miR-145-5p target and contributes to injurious effect of miR-145-5p on podocytes. We found that Arhgap24 is expressed specifically in podocytes in glomeruli (Figure S5). Luciferase reporter assay and immunoblotting showed that miR-145-5p significantly repressed Arhgap24 expression in podocytes *in vitro* (Figures 6A–6C). In mice, miR-145-5p EV administration resulted in significant reduction of Arhgap24 in podocytes, as shown by immunohistochemistry of the kidney (Figure 6D). Both *in vitro* and *in vivo*, miR-145-5p inhibitor was capable of abolishing the effect of miR-145-5p EVs on Arhgap24 expression (Figures 6C and 6D), indicating that it was miR-145-5p in the EVs that inhibited Arhgap24 expression in the podocytes. These results together supported that Arhgap24 is a direct target of miR-145-5p in podocytes. Furthermore, Arhgap24 is known to downregulate the activity of Rac1 and Cdc42,^35^ and we consistently found that miR-145-5p-transfected podocytes, in which Arhgap24 was downregulated, had increased activity of both Rac1 and Cdc42 (Figure 6E). We also tested whether miR-145-5p could downregulate Srgap1 in podocytes. As shown in Figure S6, miR-145-5p induced a marked decrease of Srgap1 protein in cultured podocytes, as well as the podocytes in mice. Srgap1 was reported to activate RhoA and inactivate Rac1.^36^ Consistently, we found that miR-145-5p decreased RhoA activity (Figure 6E), likely through its inhibition of Srgap1. We also tested glomerular Rac1/Cdc42 total protein levels in mice and found that miR-145-5p did not change the total protein levels (Figure S7).

**Table 2:** Top 10 most abundant miR families in podocytes and their targets of podocyte essential genes

miRs	let-7	miR-26-5p	miR-10a	miR-486-5p	miR-22-3p	miR-27-3p	miR-30-5p	miR-125-5p	miR-99-5p	miR-191-5p	Total
RPKM	83,700	37,646	9,938	8,870	8,185	5,951	5,352	5,689	3,802	1,883	171,016
% total (189,784)	44.1	19.8	5.2	4.7	4.3	3.1	2.8	3.0	2.0	1.0	90.1
Gene no.	12	16	9	4	13	14	16	11	2	5	average: 10.2
	Gng5	App	Fnbp1l	Tob1	Lin7c	Kiaa1109	Twf1	Podxl	Ctdspl	Chmp5	
	Apbb3	Tob1	Bbx	Prr2c	Ywhaz	Apaf1	Zdhhc21	Osbpl9	Trib2	Nfia	
	Nras	Serbp1	Dusp3	Znf207	Npnt	Aff4	Reep3	C6orf47		Lrrc8a	
	Eif4g2	Ubn2	Clasp2	Dynll1	Wapal	Nfia	Pbrm1	Enpep		Tjp1	
	Timm17b	Plod2	Son		Trib2	Pura	Lrrc58	Gpc1		Fubp3	
	Gas7	Pura	Ctdspl		Magi2	Khsrp	Qki	Wdr1			
	Snx5	Vdac1	Wapal		Apbb2	Cd2ap	Ptp4a1	Trib2			
	Tgfbr3	Trib2	Zdhhc21		Csnk1a1	Nbeal1	Vim	Dazap2			
	Swt1	Twf1	Aff4		Nras	Nedd4	Nedd4	Foxn3			
	Lgr4	Ptp4a1			Pnisr	Qki	Ywhaz	Reep3			
	Arhgap28	Eif4g2			Pcnp	Tgfbr3	Lin7c	Qki			
	Ptprd	Ywhae			Zbtb20	Lpl	Csnk1a1				
		Bbx			Ftl	Atp6v1a	Nfia				
		Srgap1				Lpin2	Ssbp2				
		Matr3					Jak1				
		Gsk3b					Smim14				

These miRNAs target a total of 72 podocyte essential genes, and GO functional annotation of the 72 genes did not yield any significant enrichment of terms, suggesting the endogenous miRNAs of podocytes do not affect podocyte homeostasis.

### miR-145-5p altered podocyte behaviors and induced injuries that involve Rho family of small GTPases

**Figure 7:**
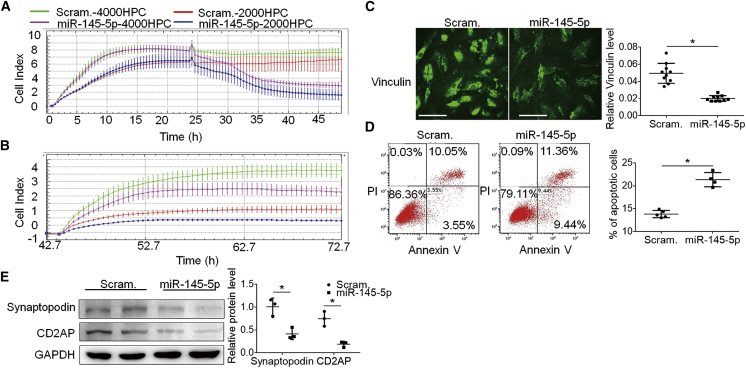
miR-145-5p significantly reduced spreading and adhesion of the podocytes and induced injury in cultured podocytes (A) RTCA of cultured podocytes transfected with miR-145-5p mimic. (B) RTCA of cultured podocytes transfected with scramble control. (C) Immunofluorescence staining of vinculin, a marker and component of focal adhesion, showing a great reduction of the protein in the cultured podocytes transfected with miR-145-5p. Scale bar: 50 μm. (D) Annexin V analysis of podocytes treated with miR-145-5p indicated increased apoptosis of the cells compared with scramble control. (E) Immunoblotting of synaptopodin and CD2AP showing their downregulation in cultured podocytes treated with miR-145-5p. Data are represented as mean ± SD. The results represented data from three independent experiments. ∗p < 0.05, statistically significant.

In addition to podocyte cytoskeletal injury induced by miR-145-5p as shown in Figure 3, which involves aberrant activity of Rho family of small GTPases, other podocyte cellular processes that involve the small GTPases include cell spreading, adhesion, migration, and apoptosis.^23^^,^^24^^,^^34^^,^^37^ Since miR-145-5p was capable of altering the Rho GTPase activities in podocytes (Figure 6), we tested miR-145-5p’s effect on spreading, adhesion, migration, and apoptosis of podocytes. Real-time cell analysis (RTCA) spreading assay showed that cell index of miR-145-5p-transfected podocytes was significantly lower than that of scramble-transfected cells (Figure 7A), indicating that miR-145-5p compromised podocyte spreading. In adhesion assay, cell index of the miR-145-5p-treated podocytes was lower than the scramble-transfected podocytes (Figure 7B); consistently, the level of vinculin, a component of focal adhesion, decreased in the miR-145-5p-treated podocytes (Figure 7C). In contrast, miR-145-5p appeared not to affect podocyte migration, as shown by both RTCA and wound-healing assays (Figures S8A and S8B). miR-145-5p-treated podocytes exhibited increased apoptosis, as shown by nuclear condensation (Figure S8C) and annexin V-fluorescein isothiocyanate/propidium iodide (PI) double staining (Figure 7D). miR-145-5p transfection also downregulated CD2AP and synaptopodin (Figure 7E), which are sensitive markers of podocyte injury (https://www.nephroseq.org/) and known to be regulated by Rho GTPases.^24^^,^^38^

### Endogenously expressed miRNAs are predicted to target few podocyte essential genes

**Figure 8:**
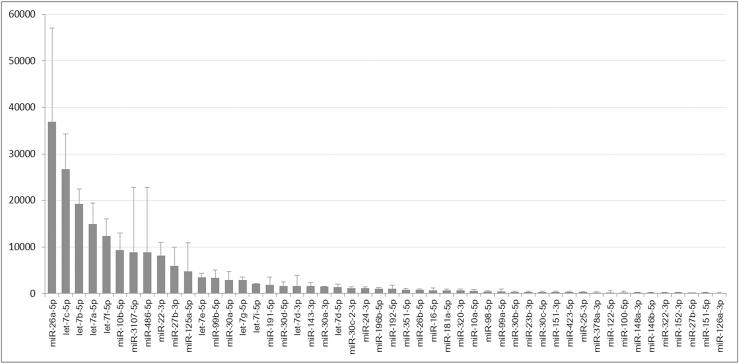
The top 50 miRNAs detected in mouse podocytes by RNA sequencing

Since the miR-145-5p is predicted to target 32 podocyte essential genes (Table 1), among which genes in the small GTPase-mediated pathway are highly enriched, we wondered how many podocyte essential genes would be targeted by the miRNAs endogenously expressed in podocytes. We sequenced miRNA contents of podocytes (Figure 8). We sorted out the 10 most abundant miRNA families, which together accounted for 90.1% of total miRNA content in podocytes (Table 2). Comparisons of their targets with the predicted 611 podocyte essential genes (Table S1) showed that they could target 2 to 16 podocyte essential genes (10.2 on average) (Table 2), which are much fewer than that of miR-145-5p (32 genes) (Table 1). Moreover, there was not any significant enrichment of genes for any specific molecular and cellular processes among the predicted target genes according to GO analysis (data not shown). From the sequencing result as shown in Figure S8, we confirmed that miR-145-5p is indeed not expressed in podocytes.

## Discussion

In the present study, we found miR-145-5p induced podocyte injury both *in vitro* and *in vivo*. We speculated that miR-145-5p could induce podocyte injury because it might effectively target podocyte essential genes, and the targeted genes are enriched in important biological processes in podocytes.

Proving the speculation requires identification of podocyte essential genes. Genome-wide identification of genes essential for a cell type was impossible until our recent study. We recently performed single-cell RNA-seq on mouse podocytes, and, based on the notion that genes that are expressed in every cell of the given cell type would be indispensable for the cell type, we identified genes essential for mouse podocytes.^19^ This work made our approach to predict a miRNA’s toxicity to podocytes possible. However, we reported only 335 podocyte essential genes in the work due to the use of the stringent cutoff of 0.5 RPKM for defining expression of a gene in a podocyte. Since the typical number of genes that are required for survival and function of a cell type is about 2,000 (9%–10% of entire genes in the genome),^31^, ^32^, ^33^ which is far beyond 335, we thus lowered the expression cutoff to 0.1 RPKM, resulting in 611 genes that were considered commonly expressed in all single podocytes and used as the predicted podocyte essential genes for the present study.

To explore how miR-145-5p is toxic to podocytes, we performed GO functional annotation of the 32 genes and found that miR-145-5p may preferentially target small GTPase-mediated signal transduction (Figure 5; Table S2). The GTPase pathway has been extensively studied in podocytes and shown to be crucial for many cellular processes in podocytes;^23^^,^^24^^,^^34^^,^^37^ we therefore focused on this pathway. There are three GTPase-activating proteins, Arhgap24, Arhgap28, and Srgap1 among predicted podocyte essential genes that are possible miR-145-5p targets. Since miRNAs act on a pathway often through inhibiting multiple components in the pathway, it is possible that inhibition of each of the three GTPase-activating proteins and other proteins contributed to the injurious effect of miR-145-5p on podocytes. We tested Arhgap24 and Srgap1, which were known to regulate the members of the Rho family of small GTPases (RhoA, Rac1, and Cdc42) in podocytes; Arhgap24 mutations can cause focal segmental glomerulosclerosis (FSGS),^35^ and Srgap1 is important for podocyte foot process maintance.^36^ The results confirmed that miR-145-5p reduced the expression of Arhgap24 and Srgap1 and induced activity alteration of RhoA, Rac1, and Cdc42. In addition to Arhgap24, Srgap1, and the other GTPase-activating proteins, other genes involving small GTPase-mediated pathways (Table 2) may also mediate miR-145-5p-induced podocyte injury (e.g., PLCE1, which mutations have been shown to cause podocyte injury and kidney diseases).^39^ Besides the pathway of small GTPase-mediated signal transduction, the other highly ranked predicted pathways enriched with miR-145-5p-targeted podocyte essential genes include those associated with cytoskeleton, cell junction, and cell protrusions (actomyosin structure organization, cell junction assembly, and axon guidance). These pathways and cellular processes have also been reported to be important for podocyte structure and function. Among the genes, Epb41l5 was reported to regulate actomyosin contractility and focal adhesion formation to maintain the kidney filtration barrier.^40^ In addition, the deletion of Podxl in podocytes was found to result in nephrotic syndrome and FSGS in mice.^41^ Thus, miR-145-5p toxicity to podocytes may involve its target genes in other important pathways in podocytes at the same time. Based on these studies, we suggest that toxicity of a miRNA is determined by whether the miRNA could effectively alter a pathway or process that is essential for the cells and that targeting effectiveness of the miRNA is determined by whether the miRNA could simultaneously target multiple components in the pathway. Accordingly, prediction of toxicity of a miRNA drug could be implemented by examining whether the miRNA could target many essential genes of the cell type and whether any pathways essential for the cell type are enriched in the functional annotation of the targeted essential genes of the cell type.

To support the validity of our approach that is based on podocyte essential genes and the enrichment of miR-145-5p targets in a critical function or process in podocytes, it would be helpful to learn how many podocyte essential genes are targeted by the miRNAs endogenously expressed in podocytes. As the endogenous miRNAs are not toxic to podocytes, they are expected to target very few podocyte essential genes. We then examined the 10 most abundant miRNA families (accounting for 90.1% of total miRNA content in podocytes) and found that they could target only 10.2 podocyte essential genes on average (Table 2). Importantly, there was not any significant enrichment of genes in a function or process according to GO analysis. These results support that our approach to predict miRNA toxicity is valid.

The applications of our method to investigate miRNA toxicity can be extended. First of all, it can be applied to any other cell types as long as they have been subjected to single-cell RNA-seq and their essential genes are thus available. At present, most cell types have undergone single-cell RNA-seq, and their essential genes can be determined by using our method.^19^ We expect that there will be databases of essential genes of all cell types that are derived from single-cell RNA-seq very soon. Based on these databases, a bioinformatics tool will be generated, which allows toxicity screening for all miRNAs in all cell types in the body, thereby facilitating the understanding of side effects of miRNA drugs, as well as the development of miRNA-based drugs, by skipping the miRNAs that are predicted to inhibit genes essential for a cell type and thus be toxic.

The idea of the approach can be adapted for extended uses. First, the approach can be used to associate diseases with the elevated levels of miRNAs in circulation and further explore roles of the miRNAs in the pathogenesis of the diseases. Second, the idea of the approach also applies to toxicity studies of miRNA inhibitor-based drugs.^42^ In this scenario, prediction and validation of upregulation of target genes of the miRNA can be conducted to identify the responsible genes whose upregulation results in injury of a cell type. Similarly, if an anti-miRNA drug is already known to be toxic to a cell type, it is feasible to identify the responsible genes in the cell type using the same approach as miRNA-based drugs.

## Materials and methods

### Cell culture and transfection, EV isolation, and labeling

Conditional immortalized human podocytes (kind gift from M. Saleem, University of Bristol, Bristol, UK) were cultured as described^43^ and are detailed in the supplemental methods. HEK293 cells were cultured in DMEM (high glucose) (Gibco-BRL, Gaithersburg, MD, USA) with 10% FBS and 1% penicillin-streptomycin. Jurkat cells were cultured in RPMI 1640 (Gibco-BRL, Gaithersburg, MD, USA) with 10% FBS and 1% penicillin-streptomycin. For transient transfection, Lipofectamine RNAiMAX (Life, China) was used following the manufacturer’s instructions. EVs were collected from cell culture medium through a series of differential centrifugation as described.^44^ The purity of EVs was assessed by electron microscopy and immunoblotting. To trace EVs *in vitro* and *in vivo*, EVs were labeled with PKH67 (Sigma) and DiR (Invitrogen) as previously described.^45^^,^^46^ Detailed methods are in the supplemental methods.

### Animals and treatment

All following animal protocols and procedures were approved by the Institutional Animal Care and Use Committee (IACUC) of Jinling Hospital (2019JLHGKJDWLS-141). Eight-week-old male BALB/c or *NPSH2-Cre*/eGFP mice were injected with EVs or miRNA agomir through the tail vein. miR-145-5p inhibitor was delivered simultaneously with EVs using TransIT-EE Delivery Solution. Kidney samples were collected after mice were euthanized. For transmission electron microscopy and quantification of podocyte foot process effacement, renal cortex was minced, fixed in 2.5% glutaraldehyde, and post-fixed in phosphate-buffered 1% osmium tetroxide. Ultrathin sections (50 nm) were stained and examined by Hitachi 7500 transmission electron microscope (Hitachi, Tokyo, Japan). Three glomeruli per mouse were evaluated with five images for each glomerulus. Resulting images were analyzed by Gatan 2.0 software. Podocyte foot process width quantification was adapted from a previous report.^47^ Mean foot process width (FPW) was calculated by the equation FPW = π/4 × (ΣGBMlength/Σfoot process). Detailed methods are in supplemental methods.

### miR# *in situ* hybridization

miR# was an artificial mutation of mmu-miR-21: mmu-miR-21 (5′-uag cuu auc aga cug augu uga-3′), mutant mmu-miR-21 (miR#) (5′-uag cuu auc aga cug caca aua-3′). Locked nucleic acid (LNA) probes were purchased from Exiqon (Copenhagen, Denmark). Mouse kidneys were fixed in 10% formaldehyde and incubated with 18% sucrose/PBS overnight at 4°C. Kidney sections (15 mm) were incubated with LNA miRNA probes labeled with digoxigenin at 55°C overnight. After wash, kidney sections were incubated with anti-digoxigenin antibody conjugated with alkaline phosphatase at 25°C for 3 h. Nitro-Blue-Tetrazolium/5-bromo-4-chloro-3-indolyl-phosphate (NBT/BCIP) (Roche) was used for color development with substrates.

### Luciferase reporter assay

The 3′ UTR of ARHGAP24 was obtained by PCR using human genomic DNA and inserted downstream of the pGL3-promoter (Promega, Madison, WI, USA). Luciferase assays were done using the Dual-Luciferase Report Assay System as described in the supplemental methods.

### Flow cytometric analysis of apoptosis via annexin V staining

Podocyte apoptosis was measured by fluorescein isothiocyanate (FITC)-conjugated annexin V/PI apoptosis kit (Multisciences, China) as detailed in the supplemental methods.

### Podocyte adhesion and wound-healing assay

Real-time adhesion and migration assays were performed using the xCELLigence system (ACEA Biosciences, China) in E-plate 16 and CIM plate16, respectively, according to the manufacturer’s instructions, and are detailed in the supplemental methods**.**

### Quantitation of the actin cytoskeleton, immunohistochemical staining, immunofluorescence staining of podocytes, western blotting, and GTPase activity assay

See supplemental methods.

### Statistical analyses

The data are presented as the mean ± SD. Differences between two groups were analyzed using the t test incorporated in Prism6 software (GraphPad Software, La Jolla, CA, USA). p < 0.05 was considered statistically significant. Differences among four groups were compared using the ANOVA method, and post hoc analyses were analyzed using the Bonferroni correction or Dunnett T correction. Differences in albuminuria between the groups at time points were analyzed by linear mixed model by SPSS 25.0 (IBM, New York, NY). Differences were considered statistically significant when the two-sided p value was less than 0.05.

## Supplemental information

Document S1. Supplemental methods, Tables S1 and S2, and Figures S1–S8

Document S2. Article plus supplemental information
