# Supplementary material for: A novel approach to identify the mechanism of miR-145-5p toxicity to podocytes based on the essential genes targeting analysis
Source: Mol Ther Nucleic Acids. 2021 Sep 20;26:749–59. doi: 10.1016/j.omtn.2021.09.005 (PMC8526908; doi:10.1016/j.omtn.2021.09.005)
Supplement: Document S1. Supplemental methods, Tables S1 and S2, and Figures S1–S8 [file mmc1.pdf]

OMTN, Volume *26*

Supplemental information

A novel approach to identify the mechanism of miR-145-5p toxicity to podocytes based on the essential genes targeting analysis

Sipan Zhang, Junnan Wu, Xiaodong Zhu, Hui Song, Lu Ren, Qiaoli Tang, Xiaodong Xu, Chunbei Liu, Jiong Zhang, Weixin Hu, Zhihong Liu, and Shaolin Shi

Supplementary information

Supplementary methods

Cell culture, treatment and transfection

Conditional immortalized human podocytes were a kind gift from Dr. M Saleem (University of Bristol, Bristol, United Kingdom). Podocytes were cultured as described [1]. Briefly, Podocytes were cultured in RPMI-1640 medium containing 10% fetal bovine serum, 1% Penicillin-Streptomycin and 1XITS (Gibco-BRL, Gaithersburg, MD, USA). Podocytes grew in 33°C and differentiated in 37°C for 14 days. HEK-293 and Jurkat (Clone E6-1) cells were purchased from Cell bank of Chinese Academy of Sciences. HEK-293 cells were cultured in DMED (high glucose) (Gibco-BRL, Gaithersburg, MD, USA) with 10% FBS and 1% Penicillin-Streptomycin in 37°C, 5% CO_2. Jurkat cells were cultured in RPMI 1640 (Gibco-BRL, Gaithersburg, MD, USA) with 10% FBS and 1% Penicillin-Streptomycin in 37°C, 5% CO_2. For transient transfection, Lipofectamine RNAiMAX (Life, Shanghai, China) was used following the manufacturer's instructions.

Animals and treatment

The use of animals in this study was approved by the Institutional Animal Care and Use Committee at Jinling Hospital. Eight weeks-old male Balb/c mice were purchased from Animal Center of Jinling Hospital, Nanjing University School of Medicine. For miR-145-5p EV experiments, thirty miligrams of EVs that were harvested from culture medium of Jurkat cells or HEK 293 cells [2] was injected through tail vein every day to mice for a total of six days. MiR-145-5p inhibitor was delivered simultaneously with EVs using TransIT®-EE Delivery Solution. Spot urine samples were collected at day 0 before injection and every day

after injection and subject to measurements of albumin and creatinine. At day 8, all mice were euthanized and the kidney samples were collected. For EV-miR biodistribution experiments, 100μg of EVs were injected into mice through tail vein. For chemically modified miRNA delivery experiments, five nanomoles of Cy5-conjugated agomir negative control (Ribio, Guangzhou, China) was injected through tail vein according to manufactures' manual. Mice were euthanized 48 hours after injection for kidney sample collection and glomeruli isolation after normal saline perfusion. To examine the effect of miR-145-5p agomir locally injected in kidney, five nanomoles miR-145-5p/Scramble agomir (Ribio, Guangzhou, China) was injected into left kidneys of mice. Three days after injection, mice were euthanized and kidney samples were collected. To examine the effect of systemically administered miR-145-5p agomir, five nanomoles of miR-145 agomir was injected into 8w male Balb/c mice through tail vein every other day. Spot urine samples were collected at day 0 before injection and the day after injection for albumin and creatinine measurements. At day 16, all mice were euthanized and kidney samples were collected. To euthanize mice, they were briefly anesthetized by inhaling Isoflurane in a chamber, followed by intraperitoneal injection of ketamine/xylazine hydrochloride solution (dose of ketamine, 100 mg/kg; xylazine, 10 mg/kg body weight). When mice were completely anesthetized, they were killed by perfusion with PBS buffer through left ventricle and the kidneys were then collected. A part of a kidney was embedded in OCT compound and snap frozen with liquid nitrogen for immunofluorescence staining and another part was placed in 4% paraformaldehyde (PFA) followed by paraffin embedding for IHC staining.

Urinary albumin and creatinine measurements

Urinary albumin and creatinine levels of the mice were measured using Albuwell M and Creatinine Companion Kits (Exocell Inc.) according to the manufacturer's instructions.

EV collection and labeling

EVs were collected from cell culture medium through a series of differential centrifugation as described in previous studies [3]. Briefly, culture medium was centrifuged at 300 g for 10 min in 4°C, to remove cells. Supernatant was then centrifuged at 2000 g for 20 min at 4°C to remove cell debris. The supernatant was then centrifuged at 100 000 g for 75 min at 4°C. Supernatant was pulled and pellet was washed in ice-cold PBS, centrifuged at 100 000 g for 75 min at 4°C. Pellets were resuspended in PBS. EV protein was quantified by BCA protein assay (Beyotime, Beijing, China). Protein quantity of EVs was the same in samples from the same amount of cells in 24 hours in different experiments. The purity of EVs was assessed by electron microscopy and immunoblotting of CD63 (Santa Cruz Biotechnology, sc-5275) and CD3-e (Santa Cruz Biotechnology, sc-1179) as described elsewhere [3].

Glomeruli isolation and sorting of podocytes

Glomeruli were isolated by Dynabead perfusion and magnetic concentration, followed by enzymatic dissociation of glomeruli and FAC sorting of EGFP-labeled podocytes [4]. Briefly, a mouse was perfused with prewarmed 20 ml PBS, 5 ml bead solution and 2 ml bead solution with digestion buffer containing 1mg/ml collagenase (C6885, Sigma-Aldrich, St. Louis, MO), 1 mg/ml Pronase E (P6911, Sigma-Aldrich) and 50 U/ml Dnase I (P5025, Sigma-Aldrich). Kidneys were decapsulated, and the cortex was minced into 1 mm^3 pieces, followed by digestion in 3 ml digestion buffer at 37°C for 5 min on a rotator (350 rpm). Digested tissues were passed through a 100-μm cell strainer and washed with PBS three times. Glomeruli were

collected by a magnetic concentrator and washed three times. The separated glomeruli were resuspended in 2 ml digestion buffer and incubated at 37°C for 30 min at 1,400 rpm/min on a thermomixer. Then, cell suspension was put on the concentrator to remove Dynabead, and sieved through a 40-μm cell strainer. The cells were centrifuged at 1,500 rpm for 5 min at 4°C. Single cells were resuspended in 0.5 ml of Hanks' buffered salt solution supplemented with 2% fetal bovine serum in 25 mM HEPES. Podocytes were sorted as EGFP-positive populations with a Beckman Coulter cell sorter with a laser excitation at 488 nm. On average, ~ 500,000 podocytes were obtained from each mouse.

miRNA sequencing

MiRNA sequencing of podocytes was performed by Kangchen Inc (Shanghai). Briefly, total RNA of the sorted EGFP-labeled mouse podocytes was prepared using the mirVana miRNA Isolation Kit (Thermo) and was used to prepare the miRNA sequencing library. The procedure included 1) 3'-adapter ligation with T4 RNA ligase 2; 2) 5'-adapter ligation with T4 RNA ligase; 3) cDNA synthesis with RT primer; 4) PCR amplification; 5) extraction and purification of ~135-155 bp PCR amplified fragments that contained both miRNA and adaptor sequences from the PAGE gel. The products of library were quantified with Agilent 2100 Bioanalyzer. Then, the DNA in the libraries were treated with 0.1 M NaOH to generate single-stranded DNA molecules that will be captured on Illumina flow cells. Then the captured DNA fragments were amplified *in situ* and sequenced for 50 cycles on Illumina HiSeq according to the manufacturer's instruction. The clean reads were processed to remove the adaptor sequence. The trimmed reads were aligned to the miRBase pre-miRNAs. miRNA read counts were normalized as tag counts per million miRNA alignments (TPM).

Transmission electron microscopy and quantification of podocyte foot process effacement

Renal cortex was minced into 1-mm^3 pieces, fixed in 2.5% glutaraldehyde and post-fixed in phosphate buffered 1% osmium tetroxide. The specimens were embedded in epoxy resin after dehydration. Ultrathin sections (50 nm) were stained. Sections were examined by Hitachi 7500 transmission electron microscope (Hitachi, Tokyo, Japan). Three glomeruli per mouse were evaluated with five images for each glomerulus. Resulting images were analyzed by Gatan 2.0 software. Podocyte foot process width quantification was adapted from previous report [5]. Mean foot process width (FPW) was calculated by the equation FPW= $\pi/4 \times (\Sigma GBMlength/\Sigma foot\ process)$.

Immunohistochemical staining

For IHC staining, 3-μm sections of the paraffin-embedded kidneys were deparaffinized and rehydrated. The slides were then blocked with 5% fetal calf serum and incubated with primary antibodies against Arhgap24 (Abcam, Cambridge, UK). Immunohistochemical staining was performed with DAB and hematoxylin. Positively stained areas were quantified with Image J software (National Institutes of Health, Bethesda, MD).

Immunofluorescence staining of podocytes

Cryosections of mouse kidneys and PFA-treated immortalized podocytes were stained using antibodies against CD2AP (H-290)(Santa Cruz Biotechnology, sc-9137), Vinculin (Proteintech, 66305-1-Ig), Synaptopodin (N-14)(Santa Cruz Biotechnology, sc-21536), Arhgap24 (Abcam, ab84046) as previously described [6].

Quantitative of the actin cytoskeleton

F-actin was stained using rhodamine-labeled phalloidin, and the resulting microscopy images were digitized. The images were converted to 8-bit and then inverted. Rhodamine stained areas were quantified using Image J software (National Institutes of Health, Bethesda, MD). Mean actin per pixel and total actin content per cell were calculated and given as arbitrary units as previously described [6].

Luciferase reporter assay

The 3'-UTR of ARHGAP24 was obtained by PCR using human genomic DNA and inserted downstream of the pGL3-promoter (Promega, Madison, WI). Site-directed mutagenesis was conducted in the region corresponding to the miR-145-5p seed. The mutations were confirmed by sequencing. The resulting constructs were co-transfected with Renilla luciferase into podocytes in culture using Lipofectamine 2000. Cell lysates were subjected to luciferase assays using the Dual-Luciferase Report Assay System 24 hours after transfection (Promega). The firefly luciferase activities were normalized to the corresponding Renilla luciferase activities.

Western blotting

Cell lysates were prepared with radioimmunoprecipitation assay (RIPA) buffer containing protease inhibitor cocktail (Roche, Indianapolis, IN) and phosphatase inhibitor. The blots were incubated with primary antibodies against CD2AP(H-290) (Santa Cruz Biotechnology, sc-9137), Arhgap24 (Abcam, ab84046), Rac1 (Santa Cruz Biotechnology, sc-192), RhoA(11) (Santa Cruz Biotechnology, sc-179), Cdc42(P1) (Santa Cruz Biotechnology, sc-87), CD3-e (Santa Cruz Biotechnology, sc-1179) and CD63 (Santa Cruz Biotechnology, sc-5275).

GTPase activity assay

To measure endogenous small GTPase activity of podocytes, the Rho-GTP pull-down assay was performed using a RhoA/Rac1/Cdc42 Activation Assay Combo Kit (Cell Biolabs, San Diego, CA). Briefly, podocytes were washed two times in cold PBS and treated with ice-cold lysis buffer. Cell lysates were incubated with the Rhotekin PBD or PAK PBD-agarose beads at 4°C for 1 h on a rotator. The beads were spun down, repeatedly washed and lysed to generate GTP-bound forms of Rac1, and Cdc42. Activated, GTP-bound forms of Rac1, and Cdc42 were evaluated by western blotting with specific antibodies to the GTPases. Total Rac1, and Cdc42 protein of each sample were blotted at the same time.

Flow cytometric analysis of apoptosis via annexin V staining

After incubation with EVs, podocytes were digested with trypsin and washed twice with ice-cold PBS. The cells were then resuspended in 500 μl binding buffer containing 5 μl FITC-conjugated annexin V and 10 μl PI at room temperature for 5 min followed by flow cytometry analysis (BC).

Podocyte adhesion and wound healing assay

Real-time adhesion and migration assay were performed using the xCELLigence system (ACEA Biosciences) in E-plate 16 and CIM plate16 respectively according to the manufacturer's instructions. Briefly, after incubation for 12 days at 37°C, differentiated podocytes were seeded at confluence in type-I collagen-coated E-plates or CIM plates and allowed to adhere overnight. In spreading assay, podocytes were transfected with miRNA mimics and scramble for 24 hours and then digested with trypsin and seeded to E-plates. In adhesion assay, podocytes were transfected with miRNA mimics and scramble for 24 hours

after seeding to RTCA plates. Adhesion and migration assays were both repeated for five times with four replicates each time. Wound healing experiment was conducted as previously described, and repeated six times in triplicate.

Supplementary Figures and Tables

Table S1. Predicted 611 podocyte essential genes.

ENSMUSG00000064352	COL4A4	TJP1	TMEM245	LAPTM4A	MT-ATP6	ACTB	COX6B1
MYOM2	Dst	NDUFB8	SQSTM1	KDELR2	RSRP1	PFN1	SPTBN1
FGFR1	Foxd2os	SPARC	PCMTD1	APLP2	ENSMUSG00000022820	MT-CYB	PPP1CB
LOC102640619	SDC4	RAD21	GRK4	SSBP2	ATP6V0E1	PSMA3	MORF4L1
IFT80	LGR4	FGD4	ENSMUSG00000085334	FUBP3	PSMC2	ITCH	RPL7
MMP12	ARHGEF18	GSN	ATP6AP1	SEP15	SPOP	Cox6c	BIRC6
SLC18B1	GADD45A	ITGA3	TMEM59	SEPT2	NKTR	ITGB1	EIF4A1
CPA6	ATP6V1B2	CD2AP	PLAT	CANX	YME1L1	MYL6	APBB2
ENSMUSG00000023737	ARPC1A	SMG1	KIAA1107	CAPS2	DYNLL1	KHSRP	SON
ENSMUSG00000081552	GPC1	Ptprd	Neat1	LRRC58	CLK1	ALKBH5	PIGV
ZNHIT3	SCHIP1	TMED7	HTRA1	NDUFA1	ATP5J	ZFR	PTGES3
TMEM69	PDIA4	ACOT2	USP9X	KIF1B	GAS5	AFF4	UBN2
AOX1	CLIC5	ITM2B	CD81	DECR2	YWHAQ	ATP5J2	RPS29
Vmn2r55	MPP5	UCP2	ORC3	ENSMUSG00000096808	OGT	MT-CO3	PRDX1
C920009B18Rik	ATG16L1	ENSMUSG00000090353	ENSMUSG00000085279	MRPL20	YBX1	TAX1BP1	SNU13
ENSMUSG00000086967	SRGAP1	NOP10	ATP5A1	Dync1i2	GATAD1	IFFO1	RPL26
ENSMUSG00000085950	HLA-A	ENSMUSG00000097287	ATP6V1G1	VIM	ACTN4	Ndufs5	Foxn3
Gm6211	ENSMUSG00000057577	PKIB	ST13	MYCBP2	RBM39	CST3	RPS14

ALOX15B	HLA-A	EHD2	DPYSL2	PTP4A1	HNRNPL	MIER1	AKR1A1
ENSMUSG00000085586	HYPK	KIF5B	POMP	BCAT2	LYPLA1	CDC42BPA	ENSMUSG00000093760
Gm16222	TSPAN13	ENSMUSG00000094472	KRCC1	RBMS3	RMDN1	PBRM1	Rps27/Rps27rt
ZNF253	SEMA3G	SYNJ2BP	RBM26	MALAT1	SMIM14	MTMR2	Eif1
D330041H03Rik	ZNF277	TSPAN15	CALR	GLRX2	NDUFA6	TOP1	FKBP1A
TOP2A	VEGFA	TIMM17B	JUP	SRP14	TMEM30A	RPL23	RPN2
SLC25A43	TNFRSF10A	ENSMUSG00000097911	RASL11A	DUSP3	Atp5e	RAB11B	CROT
S1PR4	PRDX3	Calm1 (includes others)	NPR3	RPS27L	APAF1	SOD1	RPS18
AIM1L	ITGB5	PTRF	PNISR	COX6A1	SBDS	MT-ND2	RPL38
Gm33780	RACGAP1	NFE2L1	ANXA2	IER3IP1	SEPW1	WTAP	SEPT7
SMCO1	Scd2	ERMP1	SSR3	GSK3B	MT-ND6	UNC13D	GNG5
HELLS	ENSMUSG00000090286	SUCLA2	PDLIM2	TMBIM6	TM4SF1	NDUFA7	CDC26
CLIC3	THSD7A	PHYKPL	SERBP1	MRFAP1	CTDSPL	ATP5C1	Gm16702
AIF1L	ZBTB8OS	ACSL4	TLN1	VPS53	SRPK1	ZAK	9930111J21Rik2
TCF21	Nes	BBX	HSPB11	RPL35	NRAS	ZSCAN26	ENSMUSG00000098183
YIPF1	Podxl	SEPT11	DENND5B	PTBP3	COX8A	IGFBP7	SENP1
DOCK5	MERTK	TIMP3	PAM	UQCR11	EEF1A1	H3F3A/H3F3B	RPL37
ANXA4	Nebl	ENSMUSG00000081471	SMDT1	SMIM10L1	N4BP2L2	MT-CO1	RPL35A
P3H2	NPHS1	PLOD2	SNX5	TGFBR3	Rbm25	MT-ND5	Rps3a1
ENPEP	ILDR2	TRIB2	ZNF207	KIAA1109	TMEM50A	EIF4G2	RPL21
HAUS8	TOB1	UBL5	LIN7C	MYH9	PLS3	ARGLU1	CMPK1
RAB3B	SYNPO	FYCO1	IMMT	CCNT1	NFIA	MATR3	RPL10A
PAK1	NSF	TMEM234	Tmsb4x (includes	RBM28	CTNNA1	Ubb	TPM3
EPB41L5	TMEM80	NOTCH2	MSI2	ENSMUSG00000083594	DDX5	Gm21596/Hmgb1	RPL41

ARHGAP24	WT1	MRPS14	OAZ1	ATP5B	APBB3	DYNLRB1	CLASP2
PTPRO	PDIA6	EMC2	SCP2	CYB5A	IQGAP1	COX7B	RPL37A
EZR	LYRM9	Vmn1r63 (includes others)	CSNK1A1	H3F3A/H3F3B	ACTR2	RAC1	PPIA
GAS7	TMBIM1	VDAC1	ENSMUSG000 00071107	DYNLT3	AP2M1	Rpl32	RPL27A
FAM81A	Zfp940	HSD3B1	NDUFA3	YARS	DDX58	Cdc42	ZKSCAN3
MAGI2	PTH1R	OSBPL9	HSP90AB1	PAIP1	KLHL9	GNB1	MRPL51
Cdkn1c	ROBO2	ENSMUSG0000 0061331	MT-ND4	RPL4	WAPAL	NBEAL1	RPS24
Gm26782	MAPT	EIF3M	SLC25A3	HP1BP3	ENSMUSG000 00090262	MT-ND1	RPS7
KANK1	ENSMUSG000 00083563	TMCO1	YWHAE	ZNF488	ENSMUSG000 00097695	HSBP1	TPT1
DTNB	ARF3	R3HDM4	Rpl14-ps1	RPL8	MT-ND3	PDIA3	FTL
DPP4	mt-Atp8	DSTN	Calm1 (includes others)	PTP4A2	JAK1	RPS11	Gm33780
Cd59a	ALCAM	TSPAN3	PRRC2C	Zfp60	MYO1C	WDR1	Plscr2
MAFB	LRRFIP1	AEBP1	SRSF2	RPS5	PRMT1	ZNF3	Ptma (includes others)
SEMA3E	ATP6V1A	MRPL27	CSDE1	APP	HLA-A	ACAD9	IVD
IQGAP2	MPC2	SGIP1	ATP5G3	RAB7A	DAZAP2	RPS16	ENSMUSG000 00040078
Gm3839	TSC22D1	DYNLT1	ZBTB20	SEPP1	RPS25	NCL	CD9
TDRD5	PARVA	ENSMUSG0000 0097815	GPBP1L1	SIK2	MYLK	RPS12	ATP5L
ARHGAP28	ENSMUSG000 00072692	LUC7L3	EIF4A2	MYO1D	Nedd4	TWF1	NFRKB
VEPH1	ENSMUSG000 00094030	GPX8	Srsf5	SELK	CBX1	Rps27/Rps27r t	RPL13
NPHS2	NUPR1	RBMS2	TRAM1	TTC3	ATP5F1	PAN3	RPS8
Gm11783	Psg16	ENSMUSG0000 0067344	SEC22B	MYL12B	MRPL48	MKLN1	ENSMUSG000 00044285
SHISA3	DNAJC11	NAP1L1	HSP90B1	ATRX	RPL10	TBP	RPS23
LPIN2	ENSMUSG000 00089940	CHPT1	PCNP	CHMP2A	RPL14	SAP18	B2M
COL4A3	ITGAV	PABPC1	PDCD4	SKP1	RHOA	REEP3	CBX3

CORO2B	SEPT10	QKI	ENSMUSG000 00085328	CNBP	HNRNPU	TNS3	ZDHHC21
LCP1	MOCS2	IFNGR1	PIAS4	CLTC	C6orf47	MT-CO2	NOA1
CD59	Fnbp1l	NCK2	SPCS1	RPS20	IFITM2	YWHAZ	CDK14
GOLIM4	CDC42SE2	PDXDC1	CPNE3	SSR1	UQCRB	Rplp1 (includes	SERINC3
SH2D4A	CRYAB	TNS2	LOC102637129 /S100a11	SLC39A1	COX4I1	CHCHD2	ENSMUSG000 00064339
NPNT	LPL	FKBP8	TCP1	ZMAT1	ENSMUSG000 00004980	C19orf53	ENSMUSG000 00098178
ENSMUSG000 00092400	MTSS1	GTF2A1	TIMMDC1	NDUFA4	Tmsb4x (includes	LRRC8A	
PLCE1	CTSV	MTCH1	RHEB	PURA	FTH1	RPLP0	
ENSMUSG000 00089828	LACTB2	GPX4	TIAL1	ENSMUSG000 00027942	CDC7	SWT1	
CERS6	TMOD3	CHMP5	ANAPC16	NDUFA13	MAP1LC3B	POLDIP3	
CYB5R4	DEGS1	AP1S3	ARPC2	SET	ANXA1	RPL9	

Table S2. Functional annotation of 32 predicted podocyte essential genes targeted by miR-145-5p.

Group	Description	LogP	InTerm_InList	Symbols
1	small GTPase mediated signal transduction	-4.100057945	6/485	Nras,Plce1,Ogt,Srgap1,Arhgap24,Arhgap28,Actb
2	actomyosin structure organization	-3.600584107	4/204	Tmod3,Epb41l5,Coro2b,Arhgap28,Actb,Dpysl2,Ogt,Dst
3	Proteoglycans in cancer	-3.584433416	4/206	Actb,Nras,Pdcd4,Plce1,Magi2,Gnb1
4	cell junction assembly	-3.568448891	4/208	Dst,Mpp5,Epb41l5,Coro2b,Podxl
5	muscle cell apoptotic process	-3.265781476	3/105	Gnb1,Pdcd4,Qk
6	protein heterooligomerization	-2.737266768	3/160	Gnb1,Magi2,Ogt,Actb,Mpp5
7	Axon guidance	-2.626508664	3/175	Dpysl2,Nras,Srgap1
8	DNA conformation change	-2.403335535	3/210	Hells,Pura,Set
9	proteasomal protein catabolic process	-2.302254377	4/459	Nfe2l1,Spop,Skp1a,Ogt
10	glial cell differentiation	-2.24697087	3/239	Nfe2l1,Qk,Mpp5

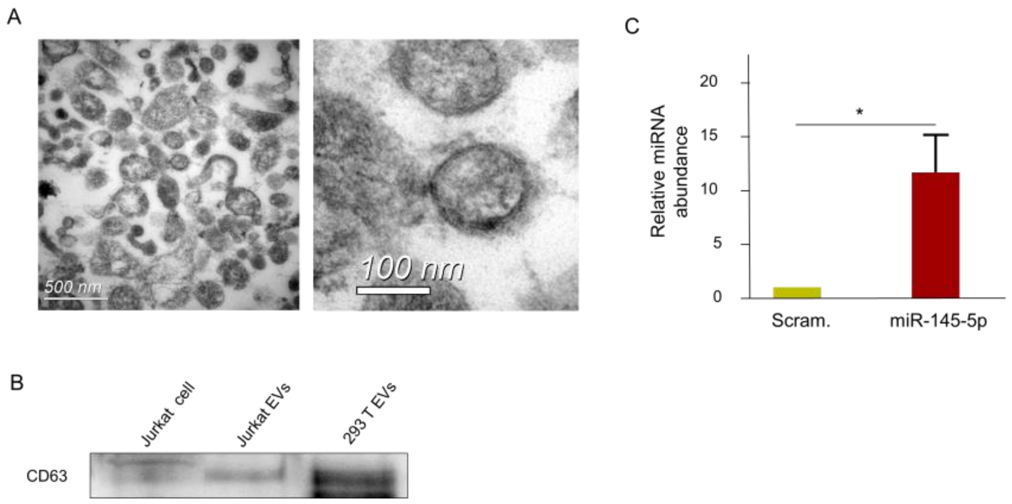

Figure S1. Characterization of miR-145-5p EM. A. EM micrographs showing morphology of the EVs. B. Immunoblotting of EV markers, CD63, which was positive for the EV samples. C. qPCR quantifications of miR-145-5p in the scramble EV and miR-145-5p EV samples (n=3), indicating that the miR-145-5p EVs had abundant miR-145-5p. * p<0.05, statistically significant.

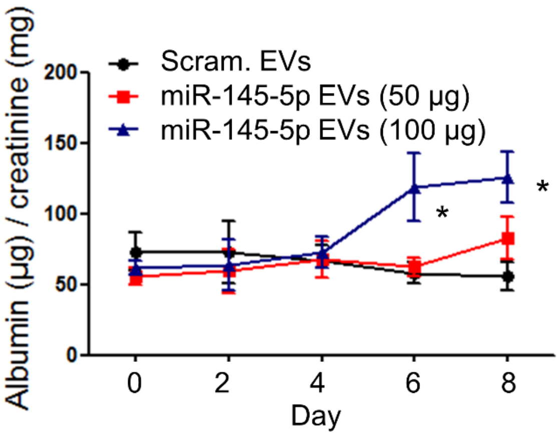

Figure S2. miR-145-5p enriched EVs induced albuminuria in healthy mice. miR-145-5p EVs were administered intravenously to mice each other day for a total of 6 days. But 100 μg dose (determined by EV-protein) of miR-145-5p EVs for every injection induced albuminuria in control mice. Data are represented as mean ±SD. *P<0.05, miR-145-5p EVs (100 μg) compared with Scram. EVs.

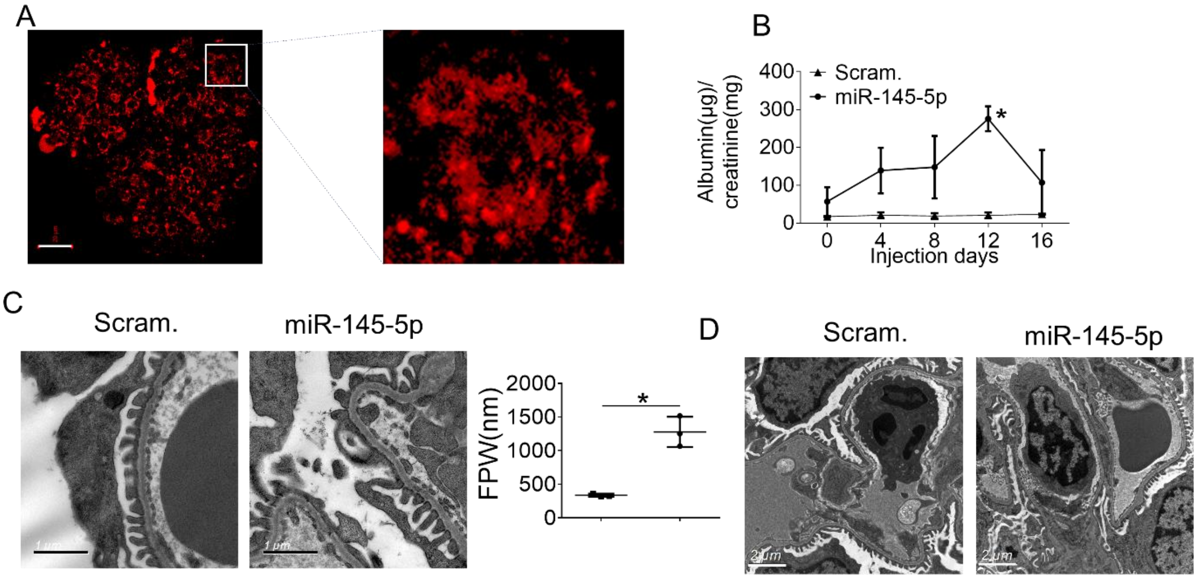

Figure S3. The in vivo effect of miR-145-5p agomir on mouse podocytes. A. Confocal microscopic imaging of a glomerulus from a mouse injected with Cy5-labeled scramble agomir via tail vein, which shows that Cy5-labeled scramble agomir was present in glomerular cells. Note that the cells surrounding capillary lumen (mainly podocytes) were heavily stained. Scale bar: 20 μm. B. Time course of albuminuria (ACR) after miR-145-5p agomir was injected through tail vein into the mice (n=3). C. EM showing podocyte foot process effacement in the mice in B. Scale bar: 1 μm. *P <0.05, statistically significant. D. EM revealed overt foot process effacement of podocytes in the mice to which miR-145-5p agomir was locally injected to the kidney. Scale bar: 2 μm.

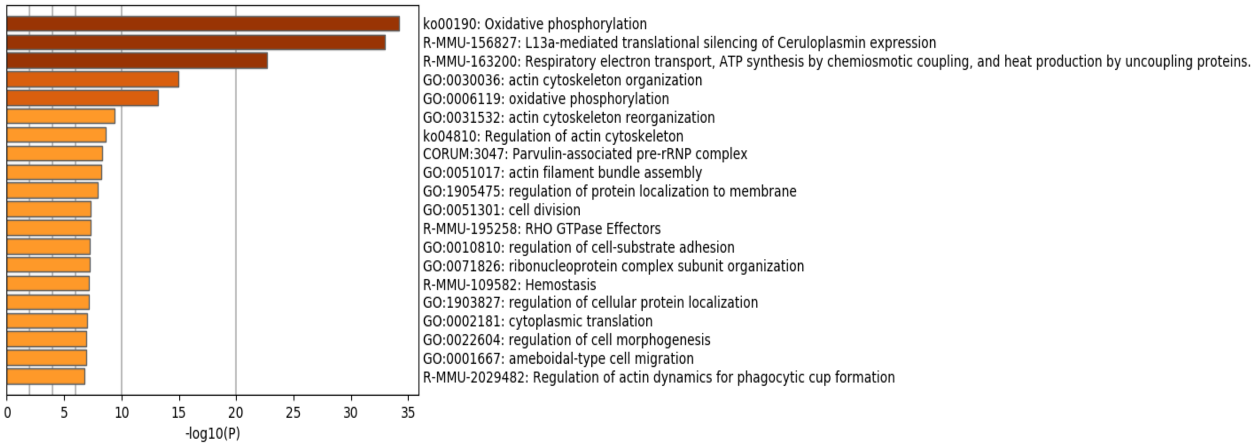

Figure S4. GO analysis of the predicted 611 podocyte essential genes.

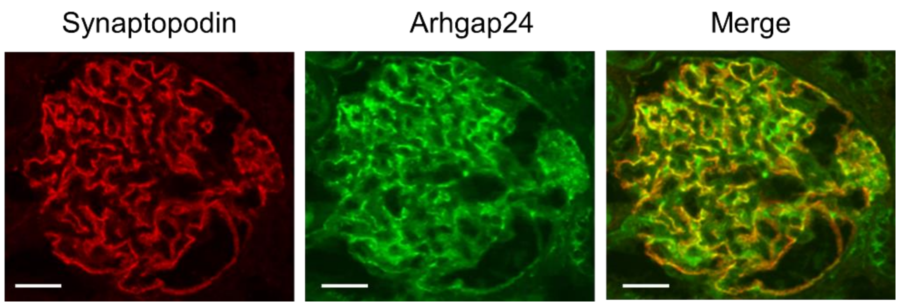

Figure S5. Immunofluorescent staining of Arhgap24 proteins in glomeruli, showing that it was colocalized with podocyte marker, synaptopodin. Scale bar: 20 μm.

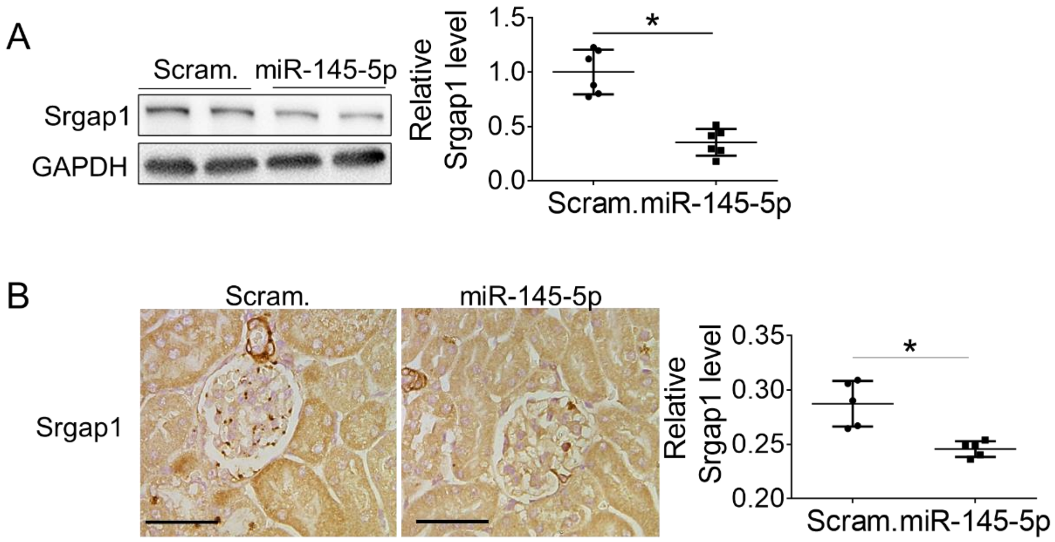

Figure S6. miR-145-5p induced Srgap1 protein loss in cultured human podocytes (A) and mice glomerular podocytes (B, n=5 mice in each group). *P<0.05. Data are represented as Mean ±SD. Scale bar, 40 μm.

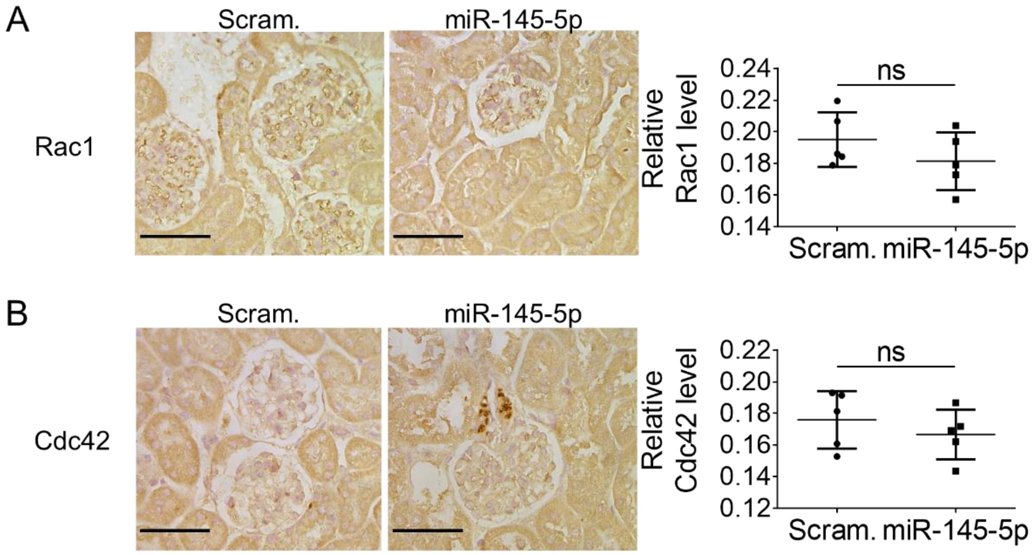

Figure S7. miR-145-5p didn't change glomerular Rac1 (A) and Cdc42 (B) total protein levels in mice. n=5 mice in each group. ns, not significant. Data are represented as Mean ±SD. Scale bar, 40 μm.

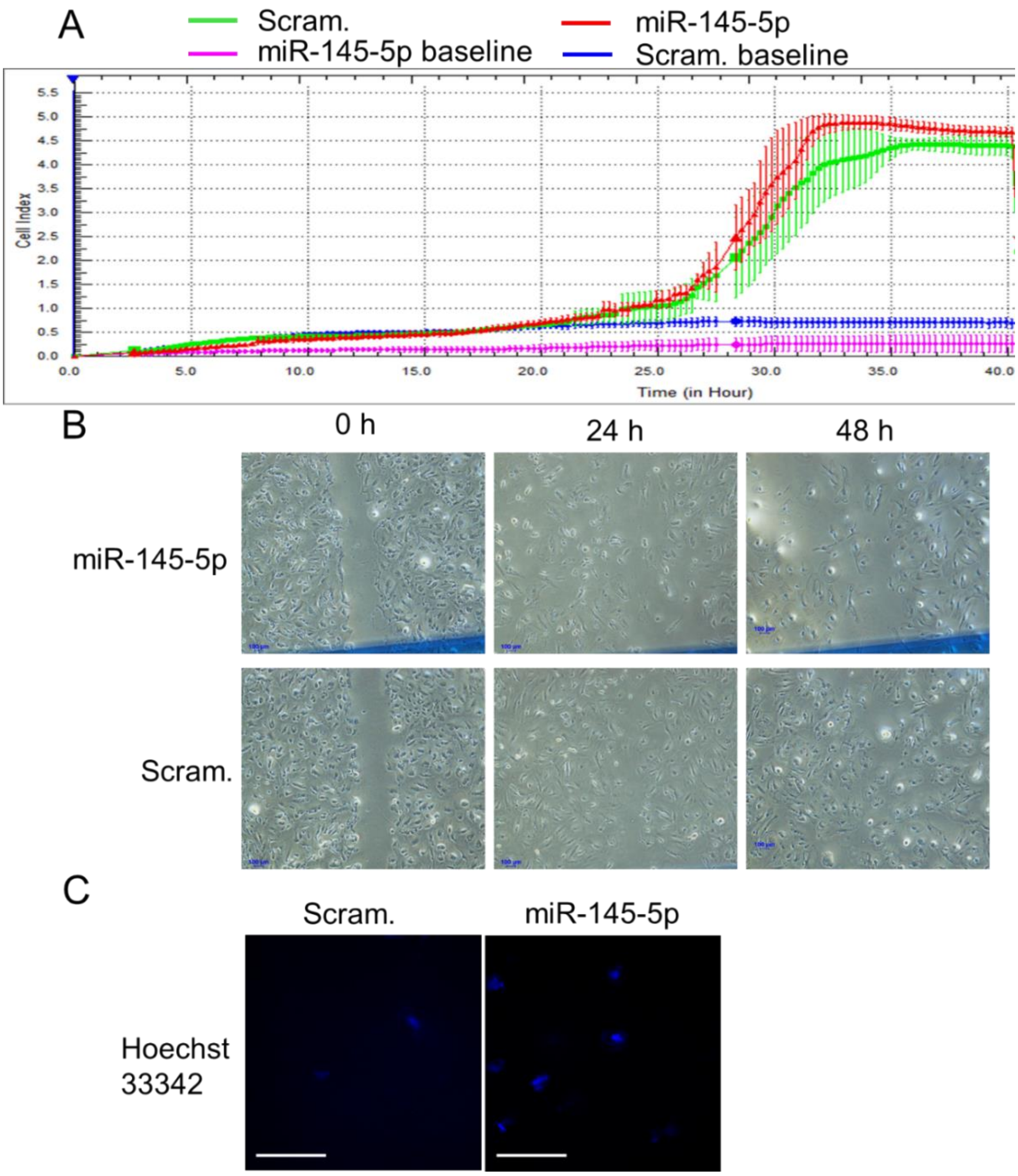

Figure S8. miR-145-5p did not affect migration but induced apoptosis of podocytes in culture. A. RTCA assay of cultured podocytes that were transfected with miR-145-5p mimic and scramble, respectively, showing no any difference between the cells treated as indicated. B. Wound healing assay showing there was no difference in scratch healing between miR-145-5p- and scramble-treated podocytes. C. Hoechst 33342 staining of the podocytes treated as indicated shows nuclear condensation that indicated the cells undergoing apoptosis. Scale bar: 100 μm.

References

1. Saleem, MA, O'Hare, MJ, Reiser, J, Coward, RJ, Inward, CD, Farren, T, *et al.* (2002). A conditionally immortalized human podocyte cell line demonstrating nephrin and podocin expression. *J Am Soc Nephrol* **13**: 630-638.

2. Bruno, S, Grange, C, Deregibus, MC, Calogero, RA, Saviozzi, S, Collino, F, *et al.* (2009). Mesenchymal Stem Cell-Derived Microvesicles Protect Against Acute Tubular Injury. *Journal of the American Society of Nephrology* **20**: 1053-1067.

3. Clotilde Th´ery, AC, Sebastian Amigorena, and Grac¸a Raposo (2006). Isolation and Characterization of Exosomes from Cell Culture Supernatants and Biological Fluids. *Current Protocols in Cell Biology*: 3.22.21-23.22.29.

4. Fu, J, Wei, C, Lee, K, Zhang, W, He, W, Chuang, P, *et al.* (2016). Comparison of Glomerular and Podocyte mRNA Profiles in Streptozotocin-Induced Diabetes. *J Am Soc Nephrol* **27**: 1006-1014.

5. van den berg, JG, van den Bergh Weerman, MA, Assmann, KJM, Weening, JJ, and Florquin, S (2004). Podocyte foot process effacement is not correlated with the level of proteinuria in human glomerulopathies. *Kidney International* **66**: 1901-1906.

6. Wu, J, Zheng, C, Wang, X, Yun, S, Zhao, Y, Liu, L, *et al.* (2015). MicroRNA-30 family members regulate calcium/calcineurin signaling in podocytes. *J Clin Invest* **125**: 4091-4106.
