# Supplementary material for: A novel approach to identify the mechanism of miR-145-5p toxicity to podocytes based on the essential genes targeting analysis
Source: Mol Ther Nucleic Acids. 2021 Sep 20;26:749–59. doi: 10.1016/j.omtn.2021.09.005 (PMC8526908; doi:10.1016/j.omtn.2021.09.005)
Supplement: Document S2. Article plus supplemental information [file mmc2.pdf]

Molecular Therapy
Nucleic Acids
Original Article

A novel approach to identify the mechanism of miR-145-5p toxicity to podocytes based on the essential genes targeting analysis

Sipan Zhang,[1] Junnan Wu,[1] Xiaodong Zhu,[1] Hui Song,[1] Lu Ren,[1] Qiaoli Tang,[1] Xiaodong Xu,[1] Chunbei Liu,[1] Jiong Zhang,[1] Weixin Hu,[1] Zhihong Liu,[1] and Shaolin Shi[1]

[1]National Clinical Research Center of Kidney Diseases, Jinling Hospital, Nanjing University School of Medicine, Nanjing, Jiangsu 21002, China

MicroRNAs (miRNAs) are emerging as effective therapeutic agents. When testing whether miR-145-5p could alleviate kidney injury, we unexpectedly found that extracellular vesicles loaded with miR-145-5p induced proteinuria and podocyte foot process effacement in normal control mice. To explore the mechanism of miR-145-5p's toxicity to podocytes, we hypothesized that miR-145-5p could enter podocytes and inhibit genes essential for podocytes. We demonstrated that systemically administered miRNA can enter podocytes. Next, we predicted 611 podocyte essential genes based on single-cell RNA sequencing (RNA-seq) and found that 32 of them are predicted to be targeted by miR-145-5p. Functional annotation of the 32 podocyte essential genes revealed small GTPase-mediated signal transduction as the top pathway. We experimentally validated that miR-145-5p targeted Arhgap24 and Srgap1, the essential regulators of the Rho family of small GTPases, increased the activity of Rac1 and Cdc42, and reduced RhoA activity, accompanied by cellular injury, in podocytes. These results explain how miR-145-5p has deleterious effect on podocytes. Most importantly, our study provides a novel approach to investigate how a miRNA affects a given cell type, allowing not only identification of the molecular mechanism underlying an observed side effect of a miRNA drug but also prediction of miRNA drug toxicity on various cell types.

INTRODUCTION

MicroRNAs (miRNAs) are posttranscriptional modulators of gene expression, and their aberrant expression is involved in the development of many diseases. Global downregulation of miRNAs is frequently observed in tumors,[1] affecting many cellular processes, including cell cycle,[2] proliferation,[3] differentiation,[4] and apoptosis,[5] thereby facilitating tumorigenesis. Dysregulation of miRNAs is also involved in pathogenesis of non-tumoral diseases.[6] Therefore, miRNA replacement therapy is promising. For example, let-7 was found to diminish the mass of murine lung cancer,[7] and miR-26a adeno-assosiated virus suppressed tumorigenesis in mice.[8] In addition, other miRNAs have also been tested for treatment of diseases (e.g., miR-34a,[9] miR-16,[10] miR-29,[11] and miR-143/miR-145[12,13]). Therapeutic miRNAs are often loaded in liposomes,[14] minicells,[10] extracellular vehicles,[15] or other vehicles[16] and then systemically administered. Systemically administered vehicles and miRNAs can reach many normal tissues, including kidney. miRNA-based drugs eventually get to the kidney for clearance by excretion, making kidney cells more likely to receive the miRNAs. Thus, it is desirable to treat kidney diseases using miRNA-based drugs.

miR-145-5p dysregulation has been shown to be involved in kidney injury by mediating the effect of MALAT1[17] and MEG3[18] on kidney. Recently, we investigated whether systemically administered miR-145-5p could mitigate kidney injury in mouse models, and, unexpectedly, we found that treatment of normal control mice with extracellular vesicles (EVs) loaded with miR-145-5p resulted in albuminuria and podocyte foot process effacement. In the present study, we studied how the systemically administered miR-145-5p caused podocyte injury. We speculated that the circulating miR-145-5p in EVs can enter podocytes and inhibit the expression of target genes that are essential for podocytes, leading to podocyte injury and glomerular filtration barrier disruption. To prove this hypothesis, it is required to determine the essential genes of podocytes and then compare them with miR-145-5p's predicted targets in order to identify which podocyte essential genes are targeted by miR-145-5p.

Recently, we designed an approach to identify genes essential for mouse podocytes and other cell types.[19] The rationale of the approach is that individual cells of the same type exhibit tremendous difference in gene expression, with correlation coefficients of 0.1–0.5, as shown by single-cell RNA sequencing (RNA-seq) studies,[20–22] indicating that most genes are differentially expressed among individual cells of the same type, and the genes differentially expressed are

Received 4 May 2021; accepted 9 September 2021;
https://doi.org/10.1016/j.omtn.2021.09.005.

Correspondence: Shaolin Shi, PhD, National Clinical Research Center of Kidney Diseases, Jinling Hospital, Nanjing University School of Medicine, Nanjing, Jiangsu 210002, China.
E-mail: shaolin.shi@nju.edu.cn
Correspondence: Zhihong Liu, MD, National Clinical Research Center of Kidney Diseases, Jinling Hospital, Nanjing University School of Medicine, Nanjing, Jiangsu 210002, China.
E-mail: liuzhihong@nju.edu.cn

This is an open access article under the CC BY license (http://creativecommons.org/licenses/by/4.0/).

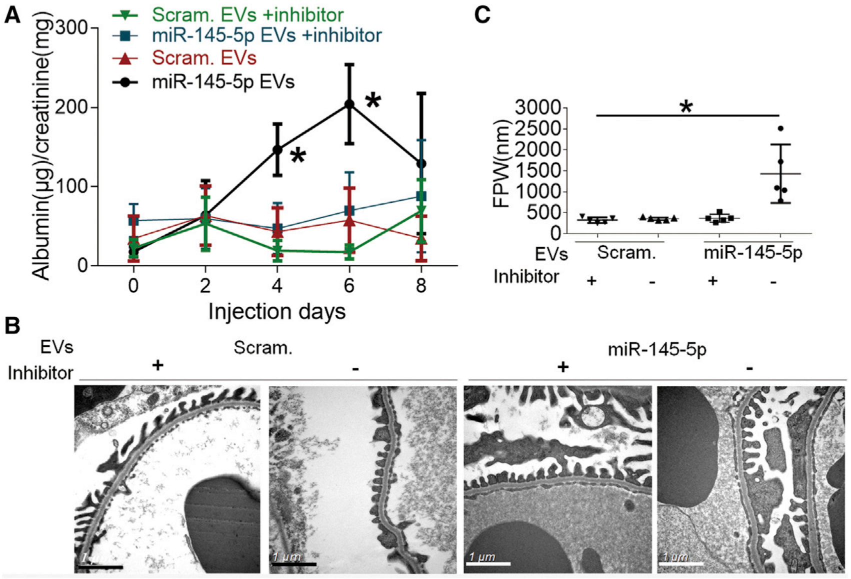

Figure 1. miR-145-5p induced podocyte injury in mice

(A) Time course of the development of proteinuria urine albumin/creatinine ratio (ACR) in the mice treated with miR-145-5p EVs in the presence (miR-145-5p EVs +inhibitor) and absence (miR-145-5p EVs) of miR-145-5p inhibitor. miR-145-5p EV sample and inhibitor were administered on days 1, 2, 3, 4, 5, and 6. (B and C) Electronic microscopy (EM) examination revealed apparent foot process effacement of the podocytes in mice treated with miR-145-5p EVs, which was reversed by miR-145-5p inhibitor. $^{*}p < 0.05$. Data are represented as mean ± SD.

dispensable, whereas those expressed commonly in all individual cells would be essential for the cell type. By this criterion, we identified candidate podocyte essential genes based on our mouse podocyte single-cell RNA-seq data and experimentally validated their essentiality for podocytes, demonstrating the feasibility of the approach.[19] The availability of podocyte essential genes made it possible to test whether miR-145-5p could target podocyte essential genes and induce podocyte injury.

We then compared the predicted miR-145-5p targets with the 611 podocyte essential genes and identified 32 miR-145-5p targets. Gene ontology (GO) analysis of the predicted 32 podocyte essential genes revealed small GTPases as the top biological process affected by miR-145-5p. Optimal activity of small GTPases is known to be crucial for podocyte survival, structure, and function.[23–25] We have proved that miR-145-5p alters small GTPase activity and induces podocyte injury. Our interpretation of the mechanism underlying miR-145-5p toxicity on podocytes provides a novel approach for understanding the mechanism underlying the side effects of miRNA-based drugs, as well as for prediction of toxicity of a miRNA drug.

RESULTS

Systemically administered miR-145-5p induced podocyte injury in mice

miR-145-5p has been reported to be dysregulated and involved in kidney injuries.[17,18] To determine whether supplement of miR-145-5p could alleviate kidney injury in mouse models, we purified the EVs from miR-145-5p transfected Jurkat or HEK293 cells and obtained the miR-145-5p-enriched EVs (Figure S1). We first tested the miR-145-5p EV samples for toxicity or side effects on healthy control mice by administering miR-145-5p EVs intravenously every other day for a total of 3 times. Unexpectedly, we found that miR-145-5p EVs induced albuminuria in the mice (Figure S2). To further confirm the albuminuria-inducing effect of miR-145-5p, we repeated the experiment with miR-145-5p EV injection once a day for a total of 6 days. To support that the toxicity of miR-145-5p EVs was miR-145-5p specific, we included a group of mice that were simultaneously injected with miR-145-5p inhibitor using TransIT-EE Delivery Solution, expecting an alleviated albuminuria in the mice. As shown in Figure 1, miR-145-5p EVs induced albuminuria and podocyte foot process effacement, and this effect was abolished by miR-145-5p inhibitor.

miRNA loaded in extracellular vesicles can reach podocytes after being systemically administered

To determine whether EVs and the enclosed miRNA can be delivered to podocytes after being administered systemically, we first performed live fluorescence imaging of the distribution of EVs labeled by DiR (a near-infrared dye) in mice after injection via tail vein. The fluorescence signal was predominantly present in liver (Figures 2A and 2B); meanwhile, abundant fluorescence was also observed in spleen and kidney, but not muscle (Figure 2B), which was in accordance with former publications.[26,27]

We next examined whether miRNA mimic labeled with Cy5 in the EVs was successfully delivered to podocytes and found that Cy5-miR-145-5p was clearly present in all glomerular cells, including podocytes that were located at the periphery of each glomerular tuft (Figure 2C). This observation was in accordance with results of *in situ* hybridization of an artificial miRNA mimic (miR#) (Figure 2D). No miR# was detected in mice injected with PBS, while in the mice injected with miR# EVs, all glomerular cells, including podocytes, were positive for miR# staining. These results were consistent with studies showing that intravenously administered EVs were readily delivered to extensive kidney tissues, including glomerular podocytes.[28,29]

Systemically administered miR-145-5p agomir entered podocytes and caused injury

We next tested the toxicity of miR-145-5p in the form of agomir, which is commonly used for miR-based drug delivery via systemic administration.[30] We first verified that Cy5-agomir injected via tail vein was able to reach podocytes (Figure S3A). Then, miR-145-5p and scramble agomirs were injected every other day for a total of 6 times, and

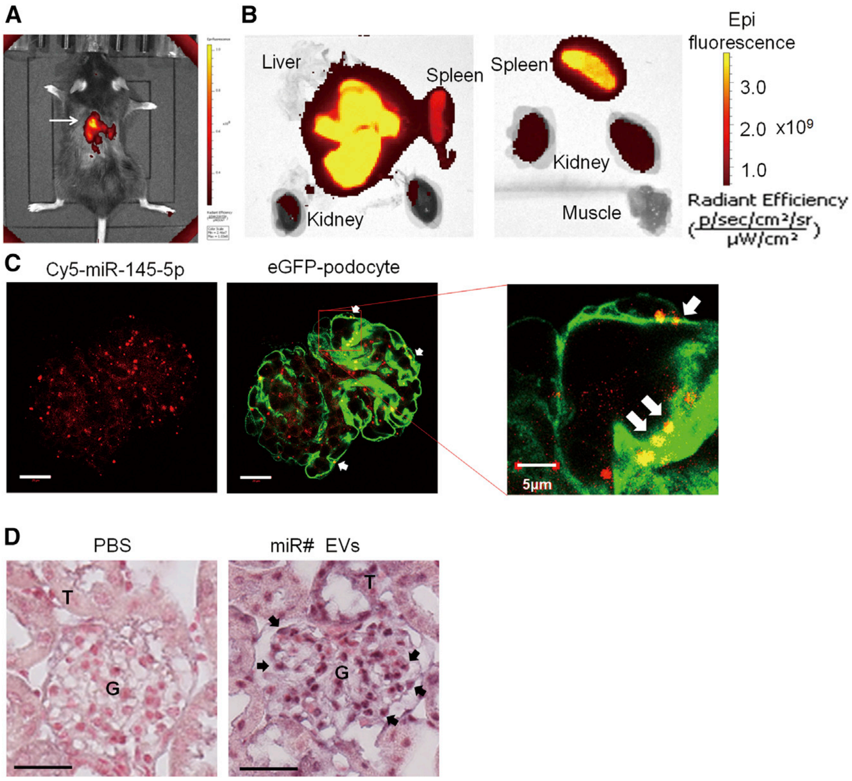

Figure 2. Tissue distribution of injected EVs and the miRNA in a mouse

(A) Fluorescence imaging of the mouse injected with DiR-labeled EVs through tail vein. The image was taken 30 min after the injection, showing predominant fluorescence signal in liver as well as other tissues. (B) Fluorescence imaging of individual organs from the same mouse, showing that EVs were accumulated in liver, kidney, and spleen but not muscles. (C) Confocal imaging of Cy5-miR-145-5p in an isolated glomerulus 48 h after the Cy5-miR-145-5p-loaded EVs were injected via tail vein to a *Npsh2*-cre/eGFP transgenic mouse whose podocytes express eGFP. Arrows: Cy5-miR-145-5p-loaded EVs accumulated in podocytes. Scale bar: 20 μm. (D) *In situ* hybridization of artificial miR# in kidney after the miR#-containing EVs were injected via tail vein to a mouse. It was clearly shown that miR# was delivered to all glomerular cells, including podocytes that are localized at periphery of each glomerular tuft. Arrows: miR#-loaded EVs accumulated in podocytes. G, glomerulus; T, tubule. Scale bar: 20 μm.

proteinuria developed in the miR-145-5p agomir-treated mice but not the scramble-treated mice (Figure S3B). EM examination consistently showed podocyte foot process effacement in the miR-145-5p- but not scramble-treated mice (Figure S3C). Additionally, we injected miR-145-5p agomir locally to mouse kidney, and 3 days after injection, marked foot process effacement was observed in the kidney injected with miR-145-5p agomir but not scramble (Figure S3D).

miR-145-5p induced injury in cultured podocytes

We tested whether miR-145-5p could cause injury in immortalized podocyte cell line in culture, which is a commonly used model for podocyte research. We added miR-145-5p EVs, which were labeled with PKH67, to podocytes in culture and observed fluorescence in the cells after 12 h (Figure 3A). We then quantified the reduction of podocyte F-actin stress fibers, because loss of F-actin stress fibers is characteristic of podocyte injury.[19] The result showed that F-actin stress fibers were greatly reduced in the miR-145-5p EV-treated podocytes (Figure 3B). We also used miR-145-5p mimic to transfect the podocytes and observed a similar reduction of F-actin stress fibers in the cells (Figure 3C). The consistent toxic effect of miR-145-5p on the podocytes in culture with those in mice suggests that the podocyte cell line is a good model for exploring how miR-145-5p induces podocyte injury.

Identification of 611 podocyte essential genes by single-cell RNA-seq

We speculated that miR-145-5p is toxic to podocytes because it is not normally expressed in podocytes, and exogenous miR-145-5p can effectively target genes essential for podocytes, as shown in the schematic diagram (Figure 4). We previously performed single-cell RNA-seq of mouse podocytes and found enormous heterogeneity of gene expression among individual podocytes. By using the concept that genes commonly expressed in all individual podocytes are likely podocyte essential genes, we predicted and validated 335 podocyte essential genes when expression cutoff was set as >0.5 Reads Per Kilobase per Million mapped reads (RPKM).[19] According to the estimation that there are approximately 2,000 genes that are essential for a cell type,[31–33] we lowered the expression cutoff to >0.1 RPKM for the present study, resulting in 611 genes that are predicted to be podocyte essential genes (Table S1). GO analysis of the predicted 611 essential genes revealed housekeeping and other processes known to be essential for podocytes (e.g., actin cytoskeleton organization [Figure S4]), supporting the reliability of our prediction approach.

Identification of podocyte essential genes targeted by the miR-145-5p

To determine how many of the podocyte essential genes can be targeted by miR-145-5p, we searched Targetscan and retrieved the top 500 predicted target genes of miR-145-5p. Comparison of miR-145-5p's target genes with the predicted 611 podocyte essential genes revealed 32 podocyte essential genes that may be targeted by miR-145-5p (Table 1).

miR-145-5p is predicted to target Rho family of small GTPases in podocytes

To explore the mechanism of miR-145-5p toxicity on podocytes, we performed GO functional annotation of the 32 podocyte essential genes that are miR-145-5p potential targets (Table 1) and found small GTPase-mediated signal transduction was at the top of the functions (Figure 5; Table S2). The members of Rho family of small GTPases

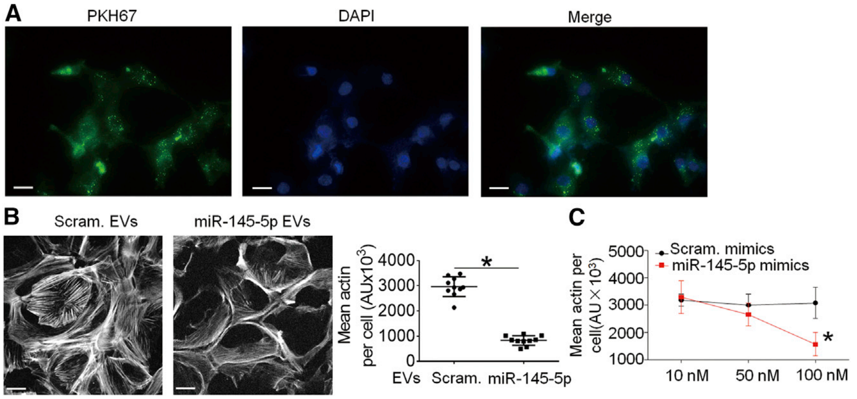

Figure 3. Toxicity of miR-145-5p on podocytes in culture

(A) Fluorescent EVs were incubated with podocytes in culture, and the fluorescence was observed in the cells after 12 h. Scale bar: 100 μm. (B) Phalloidin staining of cultured podocytes treated with miR-145-5p EVs, showing a reduction of F-actin stress fibers in the cells. Scale bar: 50 μm. (C) Quantifications of F-actin stress fibers in cultured podocytes after transfection of miR-145-5p mimic and scramble control, which shows that miR-145-5p mimic induced a reduction of F-actin stress fibers. The results represented data from three independent experiments. $^{*}p < 0.05$, statistically significant. Data are represented as mean ± SD.

(RhoA, Rac1, and Cdc42) have been best described and shown to be crucial for many cellular processes in podocytes. Aberrant activity of RhoA, Rac1, and Cdc42 has been shown to cause podocyte injury.[23,24,34]

miR-145-5p inhibited Arhgap24 and Srgap1, altering the activity of Rho family of small GTPases in podocytes

miR-145-5p was predicted to target three GTPase-activating proteins, Arhgap24, Arhgap28, and Srgap1, which are associated with the small GTPase pathway (Table 2). Since loss of Arhgap24 is known to cause podocyte injury,[35] we examined whether Arhgap24 is truly a miR-145-5p target and contributes to injurious effect of miR-145-5p on podocytes. We found that Arhgap24 is expressed specifically in podocytes in glomeruli (Figure S5). Luciferase reporter assay and immunoblotting showed that miR-145-5p significantly repressed Arhgap24 expression in podocytes *in vitro* (Figures 6A–6C). In mice, miR-145-5p EV administration resulted in significant reduction of Arhgap24 in podocytes, as shown by immunohistochemistry of the kidney (Figure 6D). Both *in vitro* and *in vivo*, miR-145-5p inhibitor was capable of abolishing the effect of miR-145-5p EVs on Arhgap24 expression (Figures 6C and 6D), indicating that it was miR-145-5p in the EVs that inhibited Arhgap24 expression in the podocytes. These results together supported that Arhgap24 is a direct target of miR-145-5p in podocytes. Furthermore, Arhgap24 is known to downregulate the activity of Rac1 and Cdc42,[35] and we consistently found that miR-145-5p-transfected podocytes, in which Arhgap24 was downregulated, had increased activity of both Rac1 and Cdc42 (Figure 6E). We also tested whether miR-145-5p could downregulate Srgap1 in podocytes. As shown in Figure S6, miR-145-5p induced a marked decrease of Srgap1 protein in cultured podocytes, as well as the podocytes in mice. Srgap1 was reported to activate RhoA and inactivate Rac1.[36] Consistently, we found that miR-145-5p decreased RhoA activity (Figure 6E), likely through its inhibition of Srgap1. We also tested glomerular Rac1/Cdc42 total protein levels in mice and found that miR-145-5p did not change the total protein levels (Figure S7).

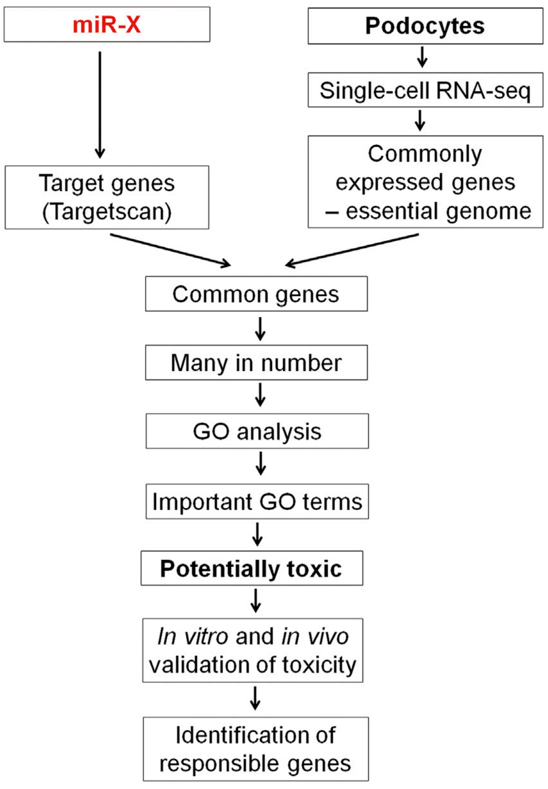

Figure 4. Schematic diagram of the approach for prediction of the toxicity of a miRNA based on its targeting of the essential genes of a cell type

miR-145-5p altered podocyte behaviors and induced injuries that involve Rho family of small GTPases

In addition to podocyte cytoskeletal injury induced by miR-145-5p as shown in Figure 3, which involves aberrant activity of Rho family of small GTPases, other podocyte cellular processes that involve the small GTPases include cell spreading, adhesion, migration, and apoptosis.[23,24,34,37] Since miR-145-5p was capable of altering the Rho GTPase activities in podocytes (Figure 6), we tested miR-145-5p's effect on spreading, adhesion, migration, and apoptosis of podocytes. Real-time cell analysis (RTCA) spreading assay showed that cell index of miR-145-5p-transfected podocytes was significantly lower than that of scramble-transfected cells (Figure 7A), indicating that miR-145-5p compromised podocyte spreading. In adhesion assay,

Table 1. Predicted targets of miR-145-5p among the 611 podocyte essential genes

Symbol	Entrez gene name	Average expression in single podocyte (RPKM)	Cumulative weighted context++ score
ACTB	actin, beta	13,889.86	−0.43
GNB1	guanine nucleotide-binding protein (G protein), beta polypeptide 1	2,439.721	−0.15
ST13	suppression of tumorigenicity 13	2,106.819	−0.04
DPYSL2	dihydropyrimidinase-like 2	1,419.238	−0.05
EIF4A2	eukaryotic translation initiation factor 4A2	1,082.761	−0.14
Podxl	podocalyxin-like	1,050.493	−0.18
SET	SET nuclear proto-oncogene	951.0317	−0.11
QKI	QKI, KH domain containing, RNA binding	776.3372	−0.12
TMOD3	tropomodulin 3 (ubiquitous)	531.727	−0.24
SKP1	S-phase kinase-associated protein 1	503.8126	−0.08
ARHGAP24	Rho GTPase-activating protein 24	452.6906	−0.23
MPP5	membrane protein, palmitoylated 5 (MAGUK p55 subfamily member 5)	270.9088	−0.35
NRAS	neuroblastoma RAS viral (v-ras) oncogene homolog	249.8165	−0.15
ACSL4	acyl-CoA synthetase long-chain family member 4	246.0129	−0.09
EPB41L5	erythrocyte membrane protein band 4.1 like 5	214.3441	−0.24
ARHGAP28	Rho GTPase-activating protein 28	199.0145	−0.07
SRGAP1	SLIT-ROBO Rho GTPase-activating protein 1	172.8949	−0.31
CORO2B	coronin, actin-binding protein, 2B	172.8764	−0.19
DENND5B	DENN/MADD domain containing 5B	152.5768	−0.12
SPOP	speckle-type POZ protein	146.9724	−0.43
OGT	O-linked N-acetylglucosamine (GlcNAc) transferase	137.9184	−0.05
PLCE1	phospholipase C, epsilon 1	128.7123	−0.29
MAGI2	membrane-associated guanylate kinase, WW and PDZ domain containing 2	120.5446	−0.33
PDCD4	programmed cell death 4 (neoplastic transformation inhibitor)	109.5968	−0.18
NFIA	nuclear factor I/A	97.51652	−0.1
Dst	dystonin	97.29717	−0.33
NFE2L1	nuclear factor, erythroid 2-like 1	56.95308	−0.17
UBN2	ubinuclein 2	44.89126	−0.1
PURA	purine-rich element binding protein A	35.38958	−0.12
SIK2	salt-inducible kinase 2	32.43537	−0.12
ERMP1	endoplasmic reticulum metallopeptidase 1	12.50254	−0.1
HELLS	helicase, lymphoid-specific	1.301073	−0.2

cell index of the miR-145-5p-treated podocytes was lower than the scramble-transfected podocytes (Figure 7B); consistently, the level of vinculin, a component of focal adhesion, decreased in the miR-145-5p-treated podocytes (Figure 7C). In contrast, miR-145-5p appeared not to affect podocyte migration, as shown by both RTCA and wound-healing assays (Figures S8A and S8B). miR-145-5p-treated podocytes exhibited increased apoptosis, as shown by nuclear condensation (Figure S8C) and annexin V-fluorescein isothiocyanate/propidium iodide (PI) double staining (Figure 7D). miR-145-5p transfection also downregulated CD2AP and synaptopodin (Figure 7E), which are sensitive markers of podocyte injury (https://www.nephroseq.org/) and known to be regulated by Rho GTPases.[24,38]

Endogenously expressed miRNAs are predicted to target few podocyte essential genes

Since the miR-145-5p is predicted to target 32 podocyte essential genes (Table 1), among which genes in the small GTPase-mediated

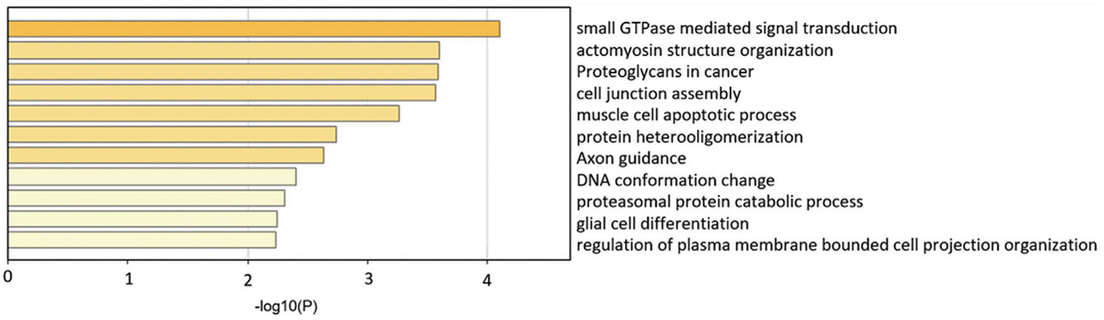

Figure 5. GO analysis of the 32 podocyte essential genes of miR-145-5p potential target

pathway are highly enriched, we wondered how many podocyte essential genes would be targeted by the miRNAs endogenously expressed in podocytes. We sequenced miRNA contents of podocytes (Figure 8). We sorted out the 10 most abundant miRNA families, which together accounted for 90.1% of total miRNA content in podocytes (Table 2). Comparisons of their targets with the predicted 611 podocyte essential genes (Table S1) showed that they could target 2 to 16 podocyte essential genes (10.2 on average) (Table 2), which are much fewer than that of miR-145-5p (32 genes) (Table 1). Moreover, there was not any significant enrichment of genes for any specific molecular and cellular processes among the predicted target genes according to GO analysis (data not shown). From the sequencing result as shown in Figure S8, we confirmed that miR-145-5p is indeed not expressed in podocytes.

DISCUSSION

In the present study, we found miR-145-5p induced podocyte injury both *in vitro* and *in vivo*. We speculated that miR-145-5p could induce podocyte injury because it might effectively target podocyte essential genes, and the targeted genes are enriched in important biological processes in podocytes.

Table 2. Top 10 most abundant miR families in podocytes and their targets of podocyte essential genes

miRs	let-7	miR-26-5p	miR-10a	miR-486-5p	miR-22-3p	miR-27-3p	miR-30-5p	miR-125-5p	miR-99-5p	miR-191-5p	Total
RPKM	83,700	37,646	9,938	8,870	8,185	5,951	5,352	5,689	3,802	1,883	171,016
% total (189,784)	44.1	19.8	5.2	4.7	4.3	3.1	2.8	3.0	2.0	1.0	90.1
Gene no.	12	16	9	4	13	14	16	11	2	5	average: 10.2
	Gng5	App	Fnbp1l	Tob1	Lin7c	Kiaa1109	Twf1	Podxl	Ctdspl	Chmp5	
	Apbb3	Tob1	Bbx	Prr2c	Ywhaz	Apaf1	Zdhhc21	Osbpl9	Trib2	Nfia	
	Nras	Serbp1	Dusp3	Znf207	Npnt	Aff4	Reep3	C6orf47		Lrrc8a	
	Eif4g2	Ubn2	Clasp2	Dynll1	Wapal	Nfia	Pbrm1	Enpep		Tjp1	
	Timm17b	Plod2	Son		Trib2	Pura	Lrrc58	Gpc1		Fubp3	
	Gas7	Pura	Ctdspl		Magi2	Khsrp	Qki	Wdr1			
	Snx5	Vdac1	Wapal		Apbb2	Cd2ap	Ptp4a1	Trib2			
	Tgfbr3	Trib2	Zdhhc21		Csnk1a1	Nbeal1	Vim	Dazap2			
	Swt1	Twf1	Aff4		Nras	Nedd4	Nedd4	Foxn3			
	Lgr4	Ptp4a1			Pnisr	Qki	Ywhaz	Reep3			
	Arhgap28	Eif4g2			Pcnp	Tgfbr3	Lin7c	Qki			
	Ptprd	Ywhae			Zbtb20	Lpl	Csnk1a1				
		Bbx			Ftl	Atp6v1a	Nfia				
		Srgap1				Lpin2	Ssbp2				
		Matr3					Jak1				
		Gsk3b					Smim14				

These miRNAs target a total of 72 podocyte essential genes, and GO functional annotation of the 72 genes did not yield any significant enrichment of terms, suggesting the endogenous miRNAs of podocytes do not affect podocyte homeostasis.

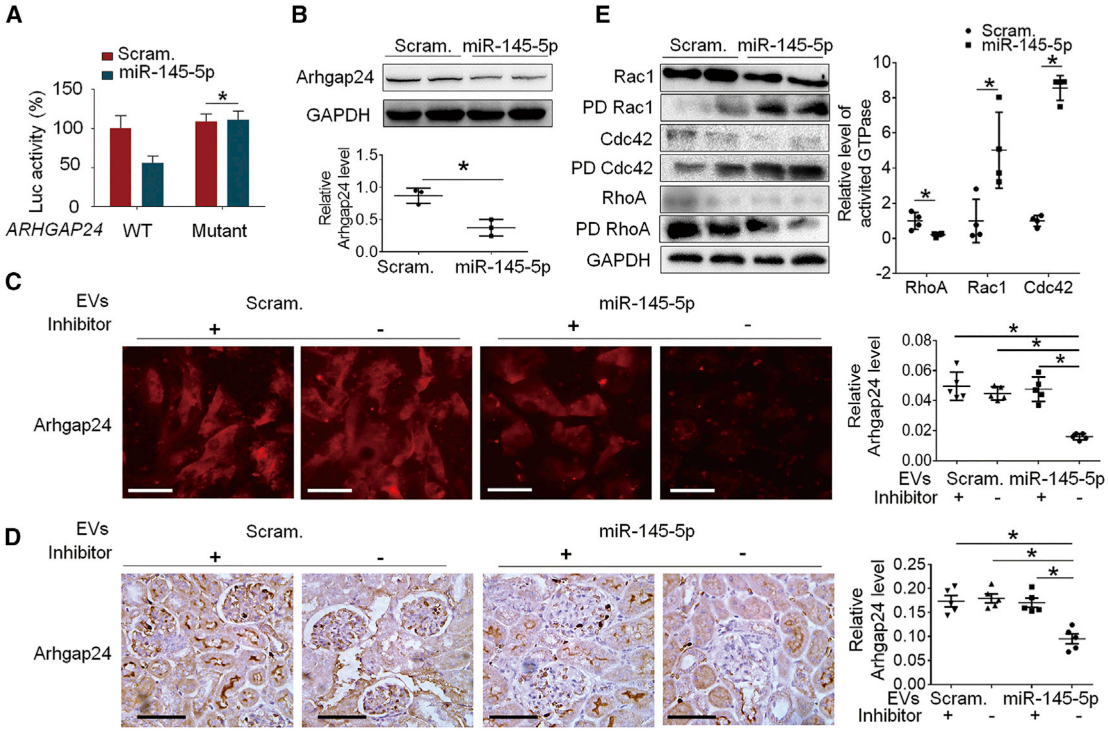

Figure 6. miR-145-5p targeted Arhgap24 and increased activity of Rac1 and Cdc42 in podocytes

(A) Luciferase reporter assay: the reporter constructs contained Arhgap24 3′ UTRs with or without mutations in the miR-145-5p binding site downstream of Luc open reading frame (ORF) were prepared and were co-transfected with miR-145-5p mimic into cultured podocytes. (B) Immunoblotting of the proteins of Arhgap24 in the cultured podocytes after transfection with miR-145-5p mimic and scramble control, showing that the Arhgap24 protein was significantly downregulated by miR-145-5p. (C) Immunofluorescence staining of cultured podocytes showed downregulation of Arhgap24 protein by the miR-145-5p in EVs, and the effect of miR-145-5p EVs was abolished by the inhibitor. Scale bar: 100 μm. (D) Immunohistochemical staining of the Arhgap24 proteins in the glomeruli of mice treated as indicated. Scale bar: 40 μm. *$p < 0.05$, statistically significant. (E) miR-145-5p upregulated Rac1 and Cdc42 activities but reduced RhoA activity as shown by immunoblotting of the total and active form (PD, pulldown) of the proteins in cultured podocytes. *$p < 0.05$, statistically significant. Data are represented as mean ± SD.

Proving the speculation requires identification of podocyte essential genes. Genome-wide identification of genes essential for a cell type was impossible until our recent study. We recently performed single-cell RNA-seq on mouse podocytes, and, based on the notion that genes that are expressed in every cell of the given cell type would be indispensable for the cell type, we identified genes essential for mouse podocytes.[19] This work made our approach to predict a miRNA's toxicity to podocytes possible. However, we reported only 335 podocyte essential genes in the work due to the use of the stringent cutoff of 0.5 RPKM for defining expression of a gene in a podocyte. Since the typical number of genes that are required for survival and function of a cell type is about 2,000 (9%–10% of entire genes in the genome),[31–33] which is far beyond 335, we thus lowered the expression cutoff to 0.1 RPKM, resulting in 611 genes that were considered commonly expressed in all single podocytes and used as the predicted podocyte essential genes for the present study.

To explore how miR-145-5p is toxic to podocytes, we performed GO functional annotation of the 32 genes and found that miR-145-5p may preferentially target small GTPase-mediated signal transduction (Figure 5; Table S2). The GTPase pathway has been extensively studied in podocytes and shown to be crucial for many cellular processes in podocytes;[23,24,34,37] we therefore focused on this pathway. There are three GTPase-activating proteins, Arhgap24, Arhgap28, and Srgap1 among predicted podocyte essential genes that are possible miR-145-5p targets. Since miRNAs act on a pathway often through inhibiting multiple components in the pathway, it is possible that inhibition of each of the three GTPase-activating proteins and other proteins contributed to the injurious effect of miR-145-5p on podocytes. We tested Arhgap24 and Srgap1, which were known to regulate the members of the Rho family of small GTPases (RhoA, Rac1, and Cdc42) in podocytes; Arhgap24 mutations can cause focal segmental glomerulosclerosis (FSGS),[35] and Srgap1 is important for podocyte foot process maintance.[36] The results confirmed that miR-145-5p reduced the expression of Arhgap24 and Srgap1 and induced activity alteration of RhoA, Rac1, and Cdc42. In addition to Arhgap24, Srgap1, and the other GTPase-activating proteins, other genes involving small GTPase-mediated pathways (Table 2) may also mediate miR-145-5p-induced podocyte injury (e.g., PLCE1, which mutations have been shown to cause podocyte injury and kidney diseases).[39] Besides the pathway of small GTPase-mediated signal transduction, the

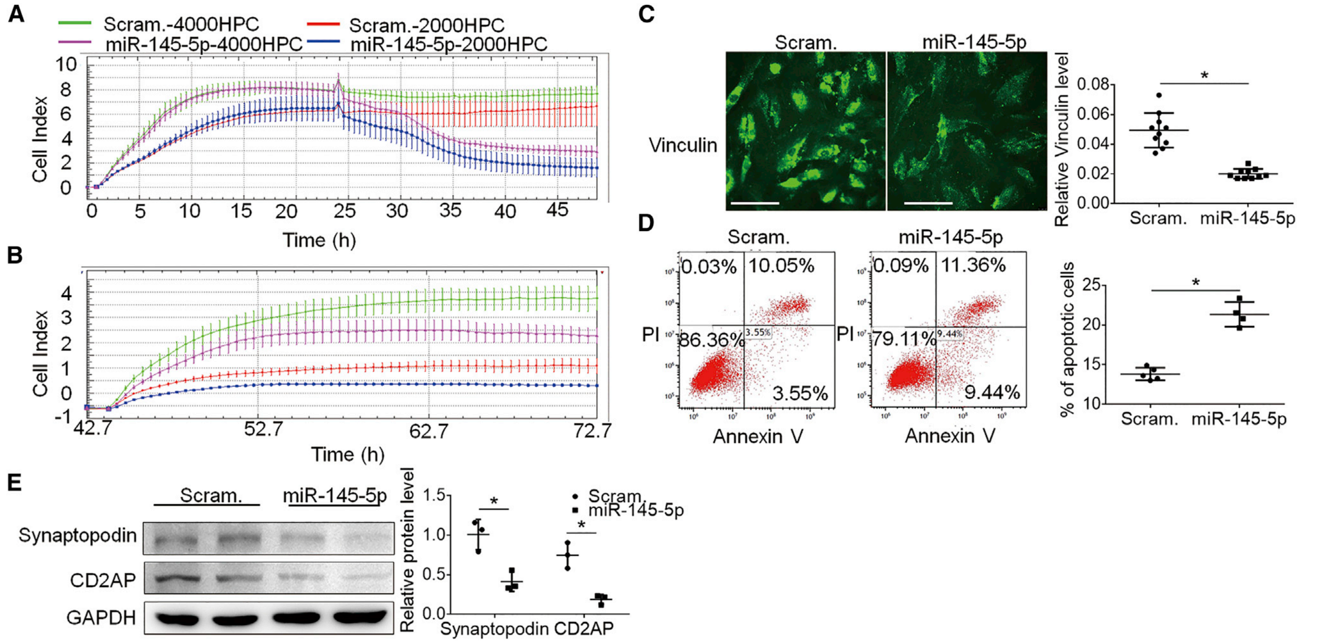

Figure 7. miR-145-5p significantly reduced spreading and adhesion of the podocytes and induced injury in cultured podocytes

(A) RTCA of cultured podocytes transfected with miR-145-5p mimic. (B) RTCA of cultured podocytes transfected with scramble control. (C) Immunofluorescence staining of vinculin, a marker and component of focal adhesion, showing a great reduction of the protein in the cultured podocytes transfected with miR-145-5p. Scale bar: 50 μm. (D) Annexin V analysis of podocytes treated with miR-145-5p indicated increased apoptosis of the cells compared with scramble control. (E) Immunoblotting of synaptopodin and CD2AP showing their downregulation in cultured podocytes treated with miR-145-5p. Data are represented as mean ± SD. The results represented data from three independent experiments. *$p < 0.05$, statistically significant.

other highly ranked predicted pathways enriched with miR-145-5p-targeted podocyte essential genes include those associated with cytoskeleton, cell junction, and cell protrusions (actomyosin structure organization, cell junction assembly, and axon guidance). These pathways and cellular processes have also been reported to be important for podocyte structure and function. Among the genes, Epb41l5 was reported to regulate actomyosin contractility and focal adhesion formation to maintain the kidney filtration barrier.[40] In addition, the deletion of Podxl in podocytes was found to result in nephrotic syndrome and FSGS in mice.[41] Thus, miR-145-5p toxicity to podocytes may involve its target genes in other important pathways in podocytes at the same time. Based on these studies, we suggest that toxicity of a miRNA is determined by whether the miRNA could effectively alter a pathway or process that is essential for the cells and that targeting effectiveness of the miRNA is determined by whether the miRNA could simultaneously target multiple components in the pathway. Accordingly, prediction of toxicity of a miRNA drug could be implemented by examining whether the miRNA could target many essential genes of the cell type and whether any pathways essential for the cell type are enriched in the functional annotation of the targeted essential genes of the cell type.

To support the validity of our approach that is based on podocyte essential genes and the enrichment of miR-145-5p targets in a critical function or process in podocytes, it would be helpful to learn how many podocyte essential genes are targeted by the miRNAs endogenously expressed in podocytes. As the endogenous miRNAs are not toxic to podocytes, they are expected to target very few podocyte essential genes. We then examined the 10 most abundant miRNA families (accounting for 90.1% of total miRNA content in podocytes) and found that they could target only 10.2 podocyte essential genes on average (Table 2). Importantly, there was not any significant enrichment of genes in a function or process according to GO analysis. These results support that our approach to predict miRNA toxicity is valid.

The applications of our method to investigate miRNA toxicity can be extended. First of all, it can be applied to any other cell types as long as they have been subjected to single-cell RNA-seq and their essential genes are thus available. At present, most cell types have undergone single-cell RNA-seq, and their essential genes can be determined by using our method.[19] We expect that there will be databases of essential genes of all cell types that are derived from single-cell RNA-seq very soon. Based on these databases, a bioinformatics tool will be generated, which allows toxicity screening for all miRNAs in all cell types in the body, thereby facilitating the understanding of side effects of miRNA drugs, as well as the development of miRNA-based drugs, by skipping the miRNAs that are predicted to inhibit genes essential for a cell type and thus be toxic.

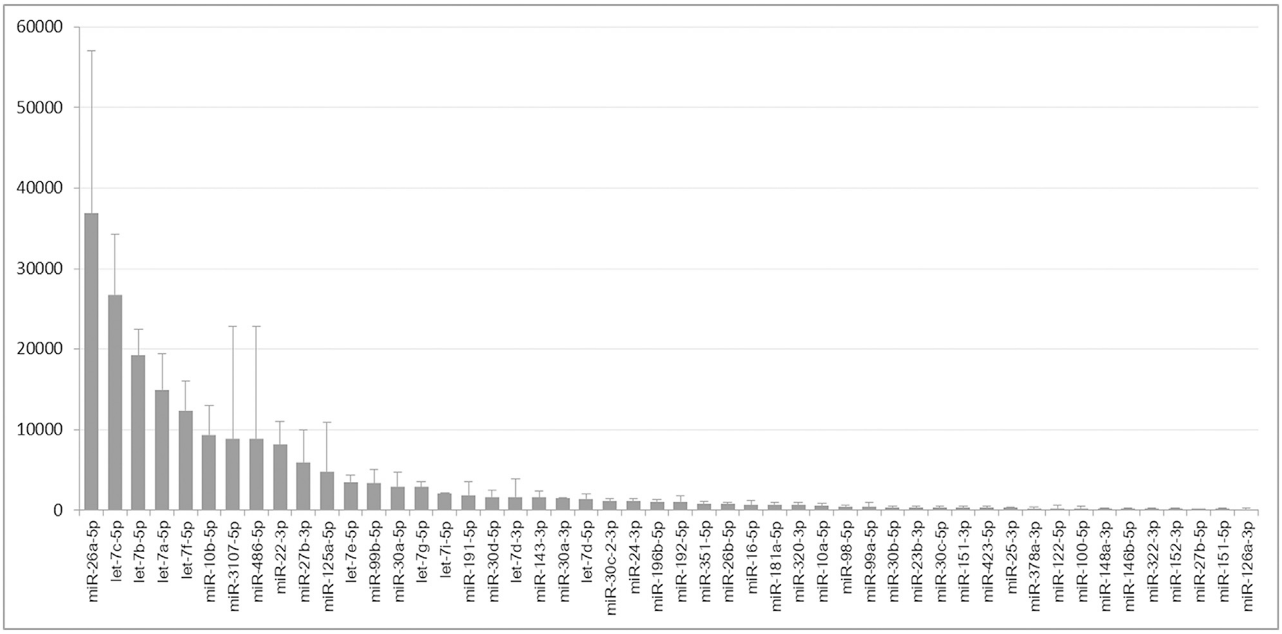

Figure 8. The top 50 miRNAs detected in mouse podocytes by RNA sequencing

The idea of the approach can be adapted for extended uses. First, the approach can be used to associate diseases with the elevated levels of miRNAs in circulation and further explore roles of the miRNAs in the pathogenesis of the diseases. Second, the idea of the approach also applies to toxicity studies of miRNA inhibitor-based drugs.[42] In this scenario, prediction and validation of upregulation of target genes of the miRNA can be conducted to identify the responsible genes whose upregulation results in injury of a cell type. Similarly, if an anti-miRNA drug is already known to be toxic to a cell type, it is feasible to identify the responsible genes in the cell type using the same approach as miRNA-based drugs.

MATERIALS AND METHODS

Cell culture and transfection, EV isolation, and labeling

Conditional immortalized human podocytes (kind gift from M. Saleem, University of Bristol, Bristol, UK) were cultured as described[43] and are detailed in the supplemental methods. HEK293 cells were cultured in DMEM (high glucose) (Gibco-BRL, Gaithersburg, MD, USA) with 10% FBS and 1% penicillin-streptomycin. Jurkat cells were cultured in RPMI 1640 (Gibco-BRL, Gaithersburg, MD, USA) with 10% FBS and 1% penicillin-streptomycin. For transient transfection, Lipofectamine RNAiMAX (Life, China) was used following the manufacturer's instructions. EVs were collected from cell culture medium through a series of differential centrifugation as described.[44] The purity of EVs was assessed by electron microscopy and immunoblotting. To trace EVs *in vitro* and *in vivo*, EVs were labeled with PKH67 (Sigma) and DiR (Invitrogen) as previously described.[45,46] Detailed methods are in the supplemental methods.

Animals and treatment

All following animal protocols and procedures were approved by the Institutional Animal Care and Use Committee (IACUC) of Jinling Hospital (2019JLHGKJDWLS-141). Eight-week-old male BALB/c or *NPSH2-Cre*/eGFP mice were injected with EVs or miRNA agomir through the tail vein. miR-145-5p inhibitor was delivered simultaneously with EVs using TransIT-EE Delivery Solution. Kidney samples were collected after mice were euthanized. For transmission electron microscopy and quantification of podocyte foot process effacement, renal cortex was minced, fixed in 2.5% glutaraldehyde, and post-fixed in phosphate-buffered 1% osmium tetroxide. Ultrathin sections (50 nm) were stained and examined by Hitachi 7500 transmission electron microscope (Hitachi, Tokyo, Japan). Three glomeruli per mouse were evaluated with five images for each glomerulus. Resulting images were analyzed by Gatan 2.0 software. Podocyte foot process width quantification was adapted from a previous report.[47] Mean foot process width (FPW) was calculated by the equation FPW = $\pi/4 \times (\Sigma \text{GBMlength}/\Sigma \text{foot process})$. Detailed methods are in supplemental methods.

miR# *in situ* hybridization

miR# was an artificial mutation of mmu-miR-21: mmu-miR-21 (5′-uag cuu auc aga cug augu uga-3′), mutant mmu-miR-21 (miR#) (5′-uag cuu auc aga cug caca aua-3′). Locked nucleic acid (LNA) probes were purchased from Exiqon (Copenhagen, Denmark). Mouse kidneys were fixed in 10% formaldehyde and incubated with 18% sucrose/PBS overnight at 4°C. Kidney sections (15 mm) were incubated with LNA miRNA probes labeled with digoxigenin at 55°C overnight. After wash, kidney sections were incubated with anti-digoxigenin antibody conjugated with alkaline phosphatase at 25°C

for 3 h. Nitro-Blue-Tetrazolium/5-bromo-4-chloro-3-indolyl-phosphate (NBT/BCIP) (Roche) was used for color development with substrates.

Luciferase reporter assay

The 3′ UTR of ARHGAP24 was obtained by PCR using human genomic DNA and inserted downstream of the pGL3-promoter (Promega, Madison, WI, USA). Luciferase assays were done using the Dual-Luciferase Report Assay System as described in the supplemental methods.

Flow cytometric analysis of apoptosis via annexin V staining

Podocyte apoptosis was measured by fluorescein isothiocyanate (FITC)-conjugated annexin V/PI apoptosis kit (Multisciences, China) as detailed in the supplemental methods.

Podocyte adhesion and wound-healing assay

Real-time adhesion and migration assays were performed using the xCELLigence system (ACEA Biosciences, China) in E-plate 16 and CIM plate16, respectively, according to the manufacturer's instructions, and are detailed in the supplemental methods.

Quantitation of the actin cytoskeleton, immunohistochemical staining, immunofluorescence staining of podocytes, western blotting, and GTPase activity assay

See supplemental methods.

Statistical analyses

The data are presented as the mean ± SD. Differences between two groups were analyzed using the t test incorporated in Prism6 software (GraphPad Software, La Jolla, CA, USA). $p < 0.05$ was considered statistically significant. Differences among four groups were compared using the ANOVA method, and post hoc analyses were analyzed using the Bonferroni correction or Dunnett T correction. Differences in albuminuria between the groups at time points were analyzed by linear mixed model by SPSS 25.0 (IBM, New York, NY). Differences were considered statistically significant when the two-sided p value was less than 0.05.

SUPPLEMENTAL INFORMATION

Supplemental information can be found online at https://doi.org/10.1016/j.omtn.2021.09.005.

ACKNOWLEDGMENTS

This work is supported by funding from the Key Research and Development Program of Jiangsu Province (BE2020698), the National Natural Science Foundation of China (82100764, 81970619, and 81770701), the Jiangsu Province Postdoctoral Research Funding Program (2019K181), and the National Key R&D Program of China (2018YFC1312705 and 2016YFC0904103). We thank Lulu Zhuang, Wanting Shi, and Yue Lang for excellent technical support.

AUTHOR CONTRIBUTIONS

S.S., Z.L., and S.Z. designed the study; S.Z., J.W., X.Z., H.S., L.R., Q.T., X.X., C.L., and J.Z. performed the experiments; S.Z., S.S., J.W., Z.L., and W.H. analyzed data; and S.Z., S.S., and Z.L. wrote the manuscript.

DECLARATION OF INTERESTS

The authors declare no competing interests.

REFERENCES

1. Lu, J., Getz, G., Miska, E.A., Alvarez-Saavedra, E., Lamb, J., Peck, D., Sweet-Cordero, A., Ebert, B.L., Mak, R.H., Ferrando, A.A., et al. (2005). MicroRNA expression profiles classify human cancers. Nature *435*, 834–838.
2. Johnson, S.M., Grosshans, H., Shingara, J., Byrom, M., Jarvis, R., Cheng, A., Labourier, E., Reinert, K.L., Brown, D., and Slack, F.J. (2005). RAS is regulated by the let-7 microRNA family. Cell *120*, 635–647.
3. Tazawa, H., Tsuchiya, N., Izumiya, M., and Nakagama, H. (2007). Tumor-suppressive miR-34a induces senescence-like growth arrest through modulation of the E2F pathway in human colon cancer cells. Proc. Natl. Acad. Sci. USA *104*, 15472–15477.
4. Xu, N., Papagiannakopoulos, T., Pan, G., Thomson, J.A., and Kosik, K.S. (2009). MicroRNA-145 regulates OCT4, SOX2, and KLF4 and represses pluripotency in human embryonic stem cells. Cell *137*, 647–658.
5. Chang, T.C., Wentzel, E.A., Kent, O.A., Ramachandran, K., Mullendore, M., Lee, K.H., Feldmann, G., Yamakuchi, M., Ferlito, M., Lowenstein, C.J., et al. (2007). Transactivation of miR-34a by p53 broadly influences gene expression and promotes apoptosis. Mol. Cell *26*, 745–752.
6. Roderburg, C., Urban, G.-W., Bettermann, K., Vucur, M., Zimmermann, H., Schmidt, S., Janssen, J., Koppe, C., Knolle, P., Castoldi, M., et al. (2011). MicroRNA profiling reveals a role for miR-29 in human and murine liver fibrosis. Hepatology *53*, 209–218.
7. Esquela-Kerscher, A., Trang, P., Wiggins, J.F., Patrawala, L., Cheng, A., Ford, L., Weidhaas, J.B., Brown, D., Bader, A.G., and Slack, F.J. (2008). The let-7 microRNA reduces tumor growth in mouse models of lung cancer. Cell Cycle *7*, 759–764.
8. Kota, J., Chivukula, R.R., O'Donnell, K.A., Wentzel, E.A., Montgomery, C.L., Hwang, H.-W., Chang, T.-C., Vivekanandan, P., Torbenson, M., Clark, K.R., et al. (2009). Therapeutic microRNA delivery suppresses tumorigenesis in a murine liver cancer model. Cell *137*, 1005–1017.
9. Liu, C., Kelnar, K., Liu, B., Chen, X., Calhoun-Davis, T., Li, H., et al. (2011). The microRNA miR-34a inhibits prostate cancer stem cells and metastasis by directly repressing CD44. Nat Med *17*, 211–215.
10. van Zandwijk, N., Pavlakis, N., Kao, S.C., Linton, A., Boyer, M.J., Clarke, S., Huynh, Y., Chrzanowska, A., Fulham, M.J., Bailey, D.L., et al. (2017). Safety and activity of microRNA-loaded minicells in patients with recurrent malignant pleural mesothelioma: a first-in-man, phase 1, open-label, dose-escalation study. Lancet Oncol. *18*, 1386–1396.
11. Xiong, Y., Fang, J.H., Yun, J.P., Yang, J., Zhang, Y., Jia, W.H., and Zhuang, S.M. (2010). Effects of microRNA-29 on apoptosis, tumorigenicity, and prognosis of hepatocellular carcinoma. Hepatology *51*, 836–845.
12. Ishii, H., Vodnala, S.K., Achyut, B.R., So, J.Y., Hollander, M.C., Greten, T.F., Lal, A., and Yang, L. (2018). miR-130a and miR-145 reprogram Gr-1$^+$CD11b$^+$ myeloid cells and inhibit tumor metastasis through improved host immunity. Nat. Commun. *9*, 2611.
13. Xu, C., Zhang, Y., Xu, K., Nie, J.J., Yu, B., Li, S., Cheng, G., Li, Y., Du, J., and Xu, F.J. (2019). Multifunctional cationic nanosystems for nucleic acid therapy of thoracic aortic dissection. Nat. Commun. *10*, 3184.
14. Beg, M.S., Brenner, A.J., Sachdev, J., Borad, M., Kang, Y.K., Stoudemire, J., Smith, S., Bader, A.G., Kim, S., and Hong, D.S. (2017). Phase I study of MRX34, a liposomal miR-34a mimic, administered twice weekly in patients with advanced solid tumors. Invest. New Drugs *35*, 180–188.
15. Usman, W.M., Pham, T.C., Kwok, Y.Y., Vu, L.T., Ma, V., Peng, B., Chan, Y.S., Wei, L., Chin, S.M., Azad, A., et al. (2018). Efficient RNA drug delivery using red blood cell extracellular vesicles. Nat. Commun. *9*, 2359.

16. Ganju, A., Khan, S., Hafeez, B.B., Behrman, S.W., Yallapu, M.M., Chauhan, S.C., and Jaggi, M. (2017). miRNA nanotherapeutics for cancer. Drug Discov. Today *22*, 424–432.

17. Liu, B., Qiang, L., Wang, G.D., Duan, Q., and Liu, J. (2019). LncRNA MALAT1 facilities high glucose induced endothelial to mesenchymal transition and fibrosis via targeting miR-145/ZEB2 axis. Eur. Rev. Med. Pharmacol. Sci. *23*, 3478–3486.

18. Liu, D., Liu, Y., Zheng, X., and Liu, N. (2021). c-MYC-induced long noncoding RNA MEG3 aggravates kidney ischemia-reperfusion injury through activating mitophagy by upregulation of RTKN to trigger the Wnt/β-catenin pathway. Cell Death Dis. *12*, 191.

19. Lu, Y., Ye, Y., Bao, W., Yang, Q., Wang, J., Liu, Z., and Shi, S. (2017). Genome-wide identification of genes essential for podocyte cytoskeletons based on single-cell RNA sequencing. Kidney Int. *92*, 1119–1129.

20. Marinov, G.K., Williams, B.A., McCue, K., Schroth, G.P., Gertz, J., Myers, R.M., and Wold, B.J. (2014). From single-cell to cell-pool transcriptomes: stochasticity in gene expression and RNA splicing. Genome Res. *24*, 496–510.

21. Raser, J.M., and O'Shea, E.K. (2005). Noise in gene expression: origins, consequences, and control. Science *309*, 2010–2013.

22. Miyamoto, D.T., Zheng, Y., Wittner, B.S., Lee, R.J., Zhu, H., Broderick, K.T., Desai, R., Fox, D.B., Brannigan, B.W., Trautwein, J., et al. (2015). RNA-Seq of single prostate CTCs implicates noncanonical Wnt signaling in antiandrogen resistance. Science *349*, 1351–1356.

23. Yu, H., Suleiman, H., Kim, A.H.J., Miner, J.H., Dani, A., Shaw, A.S., and Akilesh, S. (2013). Rac1 activation in podocytes induces rapid foot process effacement and proteinuria. Mol. Cell. Biol. *33*, 4755–4764.

24. Asanuma, K., Yanagida-Asanuma, E., Faul, C., Tomino, Y., Kim, K., and Mundel, P. (2006). Synaptopodin orchestrates actin organization and cell motility via regulation of RhoA signalling. Nat. Cell Biol. *8*, 485–491.

25. Yanagida-Asanuma, E., Asanuma, K., Kim, K., Donnelly, M., Young Choi, H., Hyung Chang, J., Suetsugu, S., Tomino, Y., Takenawa, T., Faul, C., and Mundel, P. (2007). Synaptopodin protects against proteinuria by disrupting Cdc42:IRSp53:Mena signaling complexes in kidney podocytes. Am. J. Pathol. *171*, 415–427.

26. Wiklander, O.P., Nordin, J.Z., O'Loughlin, A., Gustafsson, Y., Corso, G., Mäger, I., Vader, P., Lee, Y., Sork, H., Seow, Y., et al. (2015). Extracellular vesicle in vivo biodistribution is determined by cell source, route of administration and targeting. J. Extracell. Vesicles *4*, 26316.

27. Lai, C.P., Mardini, O., Ericsson, M., Prabhakar, S., Maguire, C., Chen, J.W., Tannous, B.A., and Breakefield, X.O. (2014). Dynamic biodistribution of extracellular vesicles in vivo using a multimodal imaging reporter. ACS Nano *8*, 483–494.

28. Cantaluppi, V., Gatti, S., Medica, D., Figliolini, F., Bruno, S., Deregibus, M.C., Sordi, A., Biancone, L., Tetta, C., and Camussi, G. (2012). Microvesicles derived from endothelial progenitor cells protect the kidney from ischemia-reperfusion injury by microRNA-dependent reprogramming of resident renal cells. Kidney Int. *82*, 412–427.

29. Ståhl, A.L., Arvidsson, I., Johansson, K.E., Chromek, M., Rebetz, J., Loos, S., Kristoffersson, A.C., Békássy, Z.D., Mörgelin, M., and Karpman, D. (2015). A novel mechanism of bacterial toxin transfer within host blood cell-derived microvesicles. PLoS Pathog. *11*, e1004619.

30. Liu, R., Lu, Z., Gu, J., Liu, J., Huang, E., Liu, X., Wang, L., Yang, J., Deng, Y., Qian, J., et al. (2018). MicroRNAs 15A and 16-1 Activate Signaling Pathways That Mediate Chemotaxis of Immune Regulatory B cells to Colorectal Tumors. Gastroenterology *154*, 637–651.e7.

31. Wang, T., Birsoy, K., Hughes, N.W., Krupczak, K.M., Post, Y., Wei, J.J., Lander, E.S., and Sabatini, D.M. (2015). Identification and characterization of essential genes in the human genome. Science *350*, 1096–1101.

32. Mair, B., Tomic, J., Masud, S.N., Tonge, P., Weiss, A., Usaj, M., Tong, A.H.Y., Kwan, J.J., Brown, K.R., Titus, E., et al. (2019). Essential Gene Profiles for Human Pluripotent Stem Cells Identify Uncharacterized Genes and Substrate Dependencies. Cell Rep. *27*, 599–615.e12.

33. Iacono, G., Massoni-Badosa, R., and Heyn, H. (2019). Single-cell transcriptomics unveils gene regulatory network plasticity. Genome Biol. *20*, 110.

34. Shibata, S., Nagase, M., Yoshida, S., Kawarazaki, W., Kurihara, H., Tanaka, H., Miyoshi, J., Takai, Y., and Fujita, T. (2008). Modification of mineralocorticoid receptor function by Rac1 GTPase: implication in proteinuric kidney disease. Nat. Med. *14*, 1370–1376.

35. Akilesh, S., Suleiman, H., Yu, H., Stander, M.C., Lavin, P., Gbadegesin, R., Antignac, C., Pollak, M., Kopp, J.B., Winn, M.P., and Shaw, A.S. (2011). Arhgap24 inactivates Rac1 in mouse podocytes, and a mutant form is associated with familial focal segmental glomerulosclerosis. J. Clin. Invest. *121*, 4127–4137.

36. Rogg, M., Maier, J.I., Dotzauer, R., Artelt, N., Kretz, O., Helmstädter, M., Abed, A., Sammarco, A., Sigle, A., Sellung, D., et al. (2021). SRGAP1 Controls Small Rho GTPases To Regulate Podocyte Foot Process Maintenance. J. Am. Soc. Nephrol. *32*, 563–579.

37. Babelova, A., Jansen, F., Sander, K., Löhn, M., Schäfer, L., Fork, C., Ruetten, H., Plettenburg, O., Stark, H., Daniel, C., et al. (2013). Activation of Rac-1 and RhoA contributes to podocyte injury in chronic kidney disease. PLoS ONE *8*, e80328.

38. Yaddanapudi, S., Altintas, M.M., Kistler, A.D., Fernandez, I., Möller, C.C., Wei, C., Peev, V., Flesche, J.B., Forst, A.L., Li, J., et al. (2011). CD2AP in mouse and human podocytes controls a proteolytic program that regulates cytoskeletal structure and cellular survival. J. Clin. Invest. *121*, 3965–3980.

39. Laurin, L.P., Lu, M., Mottl, A.K., Blyth, E.R., Poulton, C.J., and Weck, K.E. (2014). Podocyte-associated gene mutation screening in a heterogeneous cohort of patients with sporadic focal segmental glomerulosclerosis. Nephrol. Dial. Transplant. *29*, 2062–2069.

40. Schell, C., Rogg, M., Suhm, M., Helmstädter, M., Sellung, D., Yasuda-Yamahara, M., Kretz, O., Küttner, V., Suleiman, H., Kollipara, L., et al. (2017). The FERM protein EPB41L5 regulates actomyosin contractility and focal adhesion formation to maintain the kidney filtration barrier. Proc. Natl. Acad. Sci. USA *114*, E4621–E4630.

41. Refaeli, I., Hughes, M.R., Wong, A.K., Bissonnette, M.L.Z., Roskelley, C.D., Wayne Vogl, A., Barbour, S.J., Freedman, B.S., and McNagny, K.M. (2020). Distinct Functional Requirements for Podocalyxin in Immature and Mature Podocytes Reveal Mechanisms of Human Kidney Disease. Sci. Rep. *10*, 9419.

42. Adams, B.D., Parsons, C., Walker, L., Zhang, W.C., and Slack, F.J. (2017). Targeting noncoding RNAs in disease. J. Clin. Invest. *127*, 761–771.

43. Saleem, M.A., O'Hare, M.J., Reiser, J., Coward, R.J., Inward, C.D., Farren, T., Xing, C.Y., Ni, L., Mathieson, P.W., and Mundel, P. (2002). A conditionally immortalized human podocyte cell line demonstrating nephrin and podocin expression. J. Am. Soc. Nephrol. *13*, 630–638.

44. Théry, C., Amigorena, S., Raposo, G., and Clayton, A. (2006). Isolation and Characterization of Exosomes from Cell Culture Supernatants and Biological Fluids. Curr. Protoc. Cell. Biol. https://doi.org/10.1002/0471143030.cb0322s30.

45. van der Vlist, E.J., Nolte-'t Hoen, E.N., Stoorvogel, W., Arkesteijn, G.J., and Wauben, M.H. (2012). Fluorescent labeling of nano-sized vesicles released by cells and subsequent quantitative and qualitative analysis by high-resolution flow cytometry. Nat. Protoc. *7*, 1311–1326.

46. Srinivasan, S., Vannberg, F.O., and Dixon, J.B. (2016). Lymphatic transport of exosomes as a rapid route of information dissemination to the lymph node. Sci. Rep. *6*, 24436.

47. van den Berg, J.G., van den Bergh Weerman, M.A., Assmann, K.J.M., Weening, J.J., and Florquin, S. (2004). Podocyte foot process effacement is not correlated with the level of proteinuria in human glomerulopathies. Kidney Int *66*, 1901–1906.

OMTN, Volume *26*

Supplemental information

A novel approach to identify the mechanism of miR-145-5p toxicity to podocytes based on the essential genes targeting analysis

Sipan Zhang, Junnan Wu, Xiaodong Zhu, Hui Song, Lu Ren, Qiaoli Tang, Xiaodong Xu, Chunbei Liu, Jiong Zhang, Weixin Hu, Zhihong Liu, and Shaolin Shi

Supplementary information

Supplementary methods

Cell culture, treatment and transfection

Conditional immortalized human podocytes were a kind gift from Dr. M Saleem (University of Bristol, Bristol, United Kingdom). Podocytes were cultured as described [1]. Briefly, Podocytes were cultured in RPMI-1640 medium containing 10% fetal bovine serum, 1% Penicillin-Streptomycin and 1XITS (Gibco-BRL, Gaithersburg, MD, USA). Podocytes grew in 33°C and differentiated in 37°C for 14 days. HEK-293 and Jurkat (Clone E6-1) cells were purchased from Cell bank of Chinese Academy of Sciences. HEK-293 cells were cultured in DMED (high glucose) (Gibco-BRL, Gaithersburg, MD, USA) with 10% FBS and 1% Penicillin-Streptomycin in 37°C, 5% CO_2. Jurkat cells were cultured in RPMI 1640 (Gibco-BRL, Gaithersburg, MD, USA) with 10% FBS and 1% Penicillin-Streptomycin in 37°C, 5% CO_2. For transient transfection, Lipofectamine RNAiMAX (Life, Shanghai, China) was used following the manufacturer's instructions.

Animals and treatment

The use of animals in this study was approved by the Institutional Animal Care and Use Committee at Jinling Hospital. Eight weeks-old male Balb/c mice were purchased from Animal Center of Jinling Hospital, Nanjing University School of Medicine. For miR-145-5p EV experiments, thirty miligrams of EVs that were harvested from culture medium of Jurkat cells or HEK 293 cells [2] was injected through tail vein every day to mice for a total of six days. MiR-145-5p inhibitor was delivered simultaneously with EVs using TransIT®-EE Delivery Solution. Spot urine samples were collected at day 0 before injection and every day

after injection and subject to measurements of albumin and creatinine. At day 8, all mice were euthanized and the kidney samples were collected. For EV-miR biodistribution experiments, 100μg of EVs were injected into mice through tail vein. For chemically modified miRNA delivery experiments, five nanomoles of Cy5-conjugated agomir negative control (Ribio, Guangzhou, China) was injected through tail vein according to manufactures' manual. Mice were euthanized 48 hours after injection for kidney sample collection and glomeruli isolation after normal saline perfusion. To examine the effect of miR-145-5p agomir locally injected in kidney, five nanomoles miR-145-5p/Scramble agomir (Ribio, Guangzhou, China) was injected into left kidneys of mice. Three days after injection, mice were euthanized and kidney samples were collected. To examine the effect of systemically administered miR-145-5p agomir, five nanomoles of miR-145 agomir was injected into 8w male Balb/c mice through tail vein every other day. Spot urine samples were collected at day 0 before injection and the day after injection for albumin and creatinine measurements. At day 16, all mice were euthanized and kidney samples were collected. To euthanize mice, they were briefly anesthetized by inhaling Isoflurane in a chamber, followed by intraperitoneal injection of ketamine/xylazine hydrochloride solution (dose of ketamine, 100 mg/kg; xylazine, 10 mg/kg body weight). When mice were completely anesthetized, they were killed by perfusion with PBS buffer through left ventricle and the kidneys were then collected. A part of a kidney was embedded in OCT compound and snap frozen with liquid nitrogen for immunofluorescence staining and another part was placed in 4% paraformaldehyde (PFA) followed by paraffin embedding for IHC staining.

Urinary albumin and creatinine measurements

Urinary albumin and creatinine levels of the mice were measured using Albuwell M and Creatinine Companion Kits (Exocell Inc.) according to the manufacturer's instructions.

EV collection and labeling

EVs were collected from cell culture medium through a series of differential centrifugation as described in previous studies [3]. Briefly, culture medium was centrifuged at 300 g for 10 min in 4°C, to remove cells. Supernatant was then centrifuged at 2000 g for 20 min at 4°C to remove cell debris. The supernatant was then centrifuged at 100 000 g for 75 min at 4°C. Supernatant was pulled and pellet was washed in ice-cold PBS, centrifuged at 100 000 g for 75 min at 4°C. Pellets were resuspended in PBS. EV protein was quantified by BCA protein assay (Beyotime, Beijing, China). Protein quantity of EVs was the same in samples from the same amount of cells in 24 hours in different experiments. The purity of EVs was assessed by electron microscopy and immunoblotting of CD63 (Santa Cruz Biotechnology, sc-5275) and CD3-e (Santa Cruz Biotechnology, sc-1179) as described elsewhere [3].

Glomeruli isolation and sorting of podocytes

Glomeruli were isolated by Dynabead perfusion and magnetic concentration, followed by enzymatic dissociation of glomeruli and FAC sorting of EGFP-labeled podocytes [4]. Briefly, a mouse was perfused with prewarmed 20 ml PBS, 5 ml bead solution and 2 ml bead solution with digestion buffer containing 1mg/ml collagenase (C6885, Sigma-Aldrich, St. Louis, MO), 1 mg/ml Pronase E (P6911, Sigma-Aldrich) and 50 U/ml Dnase I (P5025, Sigma-Aldrich). Kidneys were decapsulated, and the cortex was minced into 1 mm^3 pieces, followed by digestion in 3 ml digestion buffer at 37°C for 5 min on a rotator (350 rpm). Digested tissues were passed through a 100-μm cell strainer and washed with PBS three times. Glomeruli were

collected by a magnetic concentrator and washed three times. The separated glomeruli were resuspended in 2 ml digestion buffer and incubated at 37°C for 30 min at 1,400 rpm/min on a thermomixer. Then, cell suspension was put on the concentrator to remove Dynabead, and sieved through a 40-μm cell strainer. The cells were centrifuged at 1,500 rpm for 5 min at 4°C. Single cells were resuspended in 0.5 ml of Hanks' buffered salt solution supplemented with 2% fetal bovine serum in 25 mM HEPES. Podocytes were sorted as EGFP-positive populations with a Beckman Coulter cell sorter with a laser excitation at 488 nm. On average, ~ 500,000 podocytes were obtained from each mouse.

miRNA sequencing

MiRNA sequencing of podocytes was performed by Kangchen Inc (Shanghai). Briefly, total RNA of the sorted EGFP-labeled mouse podocytes was prepared using the mirVana miRNA Isolation Kit (Thermo) and was used to prepare the miRNA sequencing library. The procedure included 1) 3'-adapter ligation with T4 RNA ligase 2; 2) 5'-adapter ligation with T4 RNA ligase; 3) cDNA synthesis with RT primer; 4) PCR amplification; 5) extraction and purification of ~135-155 bp PCR amplified fragments that contained both miRNA and adaptor sequences from the PAGE gel. The products of library were quantified with Agilent 2100 Bioanalyzer. Then, the DNA in the libraries were treated with 0.1 M NaOH to generate single-stranded DNA molecules that will be captured on Illumina flow cells. Then the captured DNA fragments were amplified *in situ* and sequenced for 50 cycles on Illumina HiSeq according to the manufacturer's instruction. The clean reads were processed to remove the adaptor sequence. The trimmed reads were aligned to the miRBase pre-miRNAs. miRNA read counts were normalized as tag counts per million miRNA alignments (TPM).

Transmission electron microscopy and quantification of podocyte foot process effacement

Renal cortex was minced into 1-mm^3 pieces, fixed in 2.5% glutaraldehyde and post-fixed in phosphate buffered 1% osmium tetroxide. The specimens were embedded in epoxy resin after dehydration. Ultrathin sections (50 nm) were stained. Sections were examined by Hitachi 7500 transmission electron microscope (Hitachi, Tokyo, Japan). Three glomeruli per mouse were evaluated with five images for each glomerulus. Resulting images were analyzed by Gatan 2.0 software. Podocyte foot process width quantification was adapted from previous report [5]. Mean foot process width (FPW) was calculated by the equation FPW= $\pi/4 \times (\Sigma \text{GBMlength}/\Sigma \text{foot process})$.

Immunohistochemical staining

For IHC staining, 3-μm sections of the paraffin-embedded kidneys were deparaffinized and rehydrated. The slides were then blocked with 5% fetal calf serum and incubated with primary antibodies against Arhgap24 (Abcam, Cambridge, UK). Immunohistochemical staining was performed with DAB and hematoxylin. Positively stained areas were quantified with Image J software (National Institutes of Health, Bethesda, MD).

Immunofluorescence staining of podocytes

Cryosections of mouse kidneys and PFA-treated immortalized podocytes were stained using antibodies against CD2AP (H-290)(Santa Cruz Biotechnology, sc-9137), Vinculin (Proteintech, 66305-1-Ig), Synaptopodin (N-14)(Santa Cruz Biotechnology, sc-21536), Arhgap24 (Abcam, ab84046) as previously described [6].

Quantitative of the actin cytoskeleton

F-actin was stained using rhodamine-labeled phalloidin, and the resulting microscopy images were digitized. The images were converted to 8-bit and then inverted. Rhodamine stained areas were quantified using Image J software (National Institutes of Health, Bethesda, MD). Mean actin per pixel and total actin content per cell were calculated and given as arbitrary units as previously described [6].

Luciferase reporter assay

The 3'-UTR of ARHGAP24 was obtained by PCR using human genomic DNA and inserted downstream of the pGL3-promoter (Promega, Madison, WI). Site-directed mutagenesis was conducted in the region corresponding to the miR-145-5p seed. The mutations were confirmed by sequencing. The resulting constructs were co-transfected with Renilla luciferase into podocytes in culture using Lipofectamine 2000. Cell lysates were subjected to luciferase assays using the Dual-Luciferase Report Assay System 24 hours after transfection (Promega). The firefly luciferase activities were normalized to the corresponding Renilla luciferase activities.

Western blotting

Cell lysates were prepared with radioimmunoprecipitation assay (RIPA) buffer containing protease inhibitor cocktail (Roche, Indianapolis, IN) and phosphatase inhibitor. The blots were incubated with primary antibodies against CD2AP(H-290) (Santa Cruz Biotechnology, sc-9137), Arhgap24 (Abcam, ab84046), Rac1 (Santa Cruz Biotechnology, sc-192), RhoA(11) (Santa Cruz Biotechnology, sc-179), Cdc42(P1) (Santa Cruz Biotechnology, sc-87), CD3-e (Santa Cruz Biotechnology, sc-1179) and CD63 (Santa Cruz Biotechnology, sc-5275).

GTPase activity assay

To measure endogenous small GTPase activity of podocytes, the Rho-GTP pull-down assay was performed using a RhoA/Rac1/Cdc42 Activation Assay Combo Kit (Cell Biolabs, San Diego, CA). Briefly, podocytes were washed two times in cold PBS and treated with ice-cold lysis buffer. Cell lysates were incubated with the Rhotekin PBD or PAK PBD-agarose beads at 4°C for 1 h on a rotator. The beads were spun down, repeatedly washed and lysed to generate GTP-bound forms of Rac1, and Cdc42. Activated, GTP-bound forms of Rac1, and Cdc42 were evaluated by western blotting with specific antibodies to the GTPases. Total Rac1, and Cdc42 protein of each sample were blotted at the same time.

Flow cytometric analysis of apoptosis via annexin V staining

After incubation with EVs, podocytes were digested with trypsin and washed twice with ice-cold PBS. The cells were then resuspended in 500 μl binding buffer containing 5 μl FITC-conjugated annexin V and 10 μl PI at room temperature for 5 min followed by flow cytometry analysis (BC).

Podocyte adhesion and wound healing assay

Real-time adhesion and migration assay were performed using the xCELLigence system (ACEA Biosciences) in E-plate 16 and CIM plate16 respectively according to the manufacturer’s instructions. Briefly, after incubation for 12 days at 37°C, differentiated podocytes were seeded at confluence in type-I collagen-coated E-plates or CIM plates and allowed to adhere overnight. In spreading assay, podocytes were transfected with miRNA mimics and scramble for 24 hours and then digested with trypsin and seeded to E-plates. In adhesion assay, podocytes were transfected with miRNA mimics and scramble for 24 hours

after seeding to RTCA plates. Adhesion and migration assays were both repeated for five times with four replicates each time. Wound healing experiment was conducted as previously described, and repeated six times in triplicate.

Supplementary Figures and Tables

Table S1. Predicted 611 podocyte essential genes.

ENSMUSG00000064352	COL4A4	TJP1	TMEM245	LAPTM4A	MT-ATP6	ACTB	COX6B1
MYOM2	Dst	NDUFB8	SQSTM1	KDELR2	RSRP1	PFN1	SPTBN1
FGFR1	Foxd2os	SPARC	PCMTD1	APLP2	ENSMUSG00000022820	MT-CYB	PPP1CB
LOC102640619	SDC4	RAD21	GRK4	SSBP2	ATP6V0E1	PSMA3	MORF4L1
IFT80	LGR4	FGD4	ENSMUSG00000085334	FUBP3	PSMC2	ITCH	RPL7
MMP12	ARHGEF18	GSN	ATP6AP1	SEP15	SPOP	Cox6c	BIRC6
SLC18B1	GADD45A	ITGA3	TMEM59	SEPT2	NKTR	ITGB1	EIF4A1
CPA6	ATP6V1B2	CD2AP	PLAT	CANX	YME1L1	MYL6	APBB2
ENSMUSG00000023737	ARPC1A	SMG1	KIAA1107	CAPS2	DYNLL1	KHSRP	SON
ENSMUSG00000081552	GPC1	Ptprd	Neat1	LRRC58	CLK1	ALKBH5	PIGV
ZNHIT3	SCHIP1	TMED7	HTRA1	NDUFA1	ATP5J	ZFR	PTGES3
TMEM69	PDIA4	ACOT2	USP9X	KIF1B	GAS5	AFF4	UBN2
AOX1	CLIC5	ITM2B	CD81	DECR2	YWHAQ	ATP5J2	RPS29
Vmn2r55	MPP5	UCP2	ORC3	ENSMUSG00000096808	OGT	MT-CO3	PRDX1
C920009B18Rik	ATG16L1	ENSMUSG00000090353	ENSMUSG00000085279	MRPL20	YBX1	TAX1BP1	SNU13
ENSMUSG00000086967	SRGAP1	NOP10	ATP5A1	Dync1i2	GATAD1	IFFO1	RPL26
ENSMUSG00000085950	HLA-A	ENSMUSG00000097287	ATP6V1G1	VIM	ACTN4	Ndufs5	Foxn3
Gm6211	ENSMUSG00000057577	PKIB	ST13	MYCBP2	RBM39	CST3	RPS14

ALOX15B	HLA-A	EHD2	DPYSL2	PTP4A1	HNRNPL	MIER1	AKR1A1
ENSMUSG00000085586	HYPK	KIF5B	POMP	BCAT2	LYPLA1	CDC42BPA	ENSMUSG00000093760
Gm16222	TSPAN13	ENSMUSG00000094472	KRCC1	RBMS3	RMDN1	PBRM1	Rps27/Rps27rt
ZNF253	SEMA3G	SYNJ2BP	RBM26	MALAT1	SMIM14	MTMR2	Eif1
D330041H03Rik	ZNF277	TSPAN15	CALR	GLRX2	NDUFA6	TOP1	FKBP1A
TOP2A	VEGFA	TIMM17B	JUP	SRP14	TMEM30A	RPL23	RPN2
SLC25A43	TNFRSF10A	ENSMUSG00000097911	RASL11A	DUSP3	Atp5e	RAB11B	CROT
S1PR4	PRDX3	Calm1 (includes others)	NPR3	RPS27L	APAF1	SOD1	RPS18
AIM1L	ITGB5	PTRF	PNISR	COX6A1	SBDS	MT-ND2	RPL38
Gm33780	RACGAP1	NFE2L1	ANXA2	IER3IP1	SEPW1	WTAP	SEPT7
SMCO1	Scd2	ERMP1	SSR3	GSK3B	MT-ND6	UNC13D	GNG5
HELLS	ENSMUSG00000090286	SUCLA2	PDLIM2	TMBIM6	TM4SF1	NDUFA7	CDC26
CLIC3	THSD7A	PHYKPL	SERBP1	MRFAP1	CTDSPL	ATP5C1	Gm16702
AIF1L	ZBTB8OS	ACSL4	TLN1	VPS53	SRPK1	ZAK	9930111J21Rik2
TCF21	Nes	BBX	HSPB11	RPL35	NRAS	ZSCAN26	ENSMUSG00000098183
YIPF1	Podxl	SEPT11	DENND5B	PTBP3	COX8A	IGFBP7	SENP1
DOCK5	MERTK	TIMP3	PAM	UQCR11	EEF1A1	H3F3A/H3F3B	RPL37
ANXA4	Nebl	ENSMUSG00000081471	SMDT1	SMIM10L1	N4BP2L2	MT-CO1	RPL35A
P3H2	NPHS1	PLOD2	SNX5	TGFBR3	Rbm25	MT-ND5	Rps3a1
ENPEP	ILDR2	TRIB2	ZNF207	KIAA1109	TMEM50A	EIF4G2	RPL21
HAUS8	TOB1	UBL5	LIN7C	MYH9	PLS3	ARGLU1	CMPK1
RAB3B	SYNPO	FYCO1	IMMT	CCNT1	NFIA	MATR3	RPL10A
PAK1	NSF	TMEM234	Tmsb4x (includes	RBM28	CTNNA1	Ubb	TPM3
EPB41L5	TMEM80	NOTCH2	MSI2	ENSMUSG00000083594	DDX5	Gm21596/Hmgb1	RPL41

ARHGAP24	WT1	MRPS14	OAZ1	ATP5B	APBB3	DYNLRB1	CLASP2
PTPRO	PDIA6	EMC2	SCP2	CYB5A	IQGAP1	COX7B	RPL37A
EZR	LYRM9	Vmn1r63 (includes others)	CSNK1A1	H3F3A/H3F3B	ACTR2	RAC1	PPIA
GAS7	TMBIM1	VDAC1	ENSMUSG000 00071107	DYNLT3	AP2M1	Rpl32	RPL27A
FAM81A	Zfp940	HSD3B1	NDUFA3	YARS	DDX58	Cdc42	ZKSCAN3
MAGI2	PTH1R	OSBPL9	HSP90AB1	PAIP1	KLHL9	GNB1	MRPL51
Cdkn1c	ROBO2	ENSMUSG0000 0061331	MT-ND4	RPL4	WAPAL	NBEAL1	RPS24
Gm26782	MAPT	EIF3M	SLC25A3	HP1BP3	ENSMUSG000 00090262	MT-ND1	RPS7
KANK1	ENSMUSG000 00083563	TMCO1	YWHAE	ZNF488	ENSMUSG000 00097695	HSBP1	TPT1
DTNB	ARF3	R3HDM4	Rpl14-ps1	RPL8	MT-ND3	PDIA3	FTL
DPP4	mt-Atp8	DSTN	Calm1 (includes others)	PTP4A2	JAK1	RPS11	Gm33780
Cd59a	ALCAM	TSPAN3	PRRC2C	Zfp60	MYO1C	WDR1	Plscr2
MAFB	LRRFIP1	AEBP1	SRSF2	RPS5	PRMT1	ZNF3	Ptma (includes others)
SEMA3E	ATP6V1A	MRPL27	CSDE1	APP	HLA-A	ACAD9	IVD
IQGAP2	MPC2	SGIP1	ATP5G3	RAB7A	DAZAP2	RPS16	ENSMUSG000 00040078
Gm3839	TSC22D1	DYNLT1	ZBTB20	SEPP1	RPS25	NCL	CD9
TDRD5	PARVA	ENSMUSG0000 0097815	GPBP1L1	SIK2	MYLK	RPS12	ATP5L
ARHGAP28	ENSMUSG000 00072692	LUC7L3	EIF4A2	MYO1D	Nedd4	TWF1	NFRKB
VEPH1	ENSMUSG000 00094030	GPX8	Srsf5	SELK	CBX1	Rps27/Rps27r t	RPL13
NPHS2	NUPR1	RBMS2	TRAM1	TTC3	ATP5F1	PAN3	RPS8
Gm11783	Psg16	ENSMUSG0000 0067344	SEC22B	MYL12B	MRPL48	MKLN1	ENSMUSG000 00044285
SHISA3	DNAJC11	NAP1L1	HSP90B1	ATRX	RPL10	TBP	RPS23
LPIN2	ENSMUSG000 00089940	CHPT1	PCNP	CHMP2A	RPL14	SAP18	B2M
COL4A3	ITGAV	PABPC1	PDCD4	SKP1	RHOA	REEP3	CBX3

CORO2B	SEPT10	QKI	ENSMUSG000 00085328	CNBP	HNRNPU	TNS3	ZDHHC21
LCP1	MOCS2	IFNGR1	PIAS4	CLTC	C6orf47	MT-CO2	NOA1
CD59	Fnbp1l	NCK2	SPCS1	RPS20	IFITM2	YWHAZ	CDK14
GOLIM4	CDC42SE2	PDXDC1	CPNE3	SSR1	UQCRB	Rplp1 (includes	SERINC3
SH2D4A	CRYAB	TNS2	LOC102637129 /S100a11	SLC39A1	COX4I1	CHCHD2	ENSMUSG000 00064339
NPNT	LPL	FKBP8	TCP1	ZMAT1	ENSMUSG000 00004980	C19orf53	ENSMUSG000 00098178
ENSMUSG000 00092400	MTSS1	GTF2A1	TIMMDC1	NDUFA4	Tmsb4x (includes	LRRC8A	
PLCE1	CTSV	MTCH1	RHEB	PURA	FTH1	RPLP0	
ENSMUSG000 00089828	LACTB2	GPX4	TIAL1	ENSMUSG000 00027942	CDC7	SWT1	
CERS6	TMOD3	CHMP5	ANAPC16	NDUFA13	MAP1LC3B	POLDIP3	
CYB5R4	DEGS1	AP1S3	ARPC2	SET	ANXA1	RPL9	

Table S2. Functional annotation of 32 predicted podocyte essential genes targeted by miR-145-5p.

Group	Description	LogP	InTerm_InList	Symbols
1	small GTPase mediated signal transduction	-4.100057945	6/485	Nras,Plce1,Ogt,Srgap1,Arhgap24,Arhgap28,Actb
2	actomyosin structure organization	-3.600584107	4/204	Tmod3,Epb41l5,Coro2b,Arhgap28,Actb,Dpysl2,Ogt,Dst
3	Proteoglycans in cancer	-3.584433416	4/206	Actb,Nras,Pdcd4,Plce1,Magi2,Gnb1
4	cell junction assembly	-3.568448891	4/208	Dst,Mpp5,Epb41l5,Coro2b,Podxl
5	muscle cell apoptotic process	-3.265781476	3/105	Gnb1,Pdcd4,Qk
6	protein heterooligomerization	-2.737266768	3/160	Gnb1,Magi2,Ogt,Actb,Mpp5
7	Axon guidance	-2.626508664	3/175	Dpysl2,Nras,Srgap1
8	DNA conformation change	-2.403335535	3/210	Hells,Pura,Set
9	proteasomal protein catabolic process	-2.302254377	4/459	Nfe2l1,Spop,Skp1a,Ogt
10	glial cell differentiation	-2.24697087	3/239	Nfe2l1,Qk,Mpp5

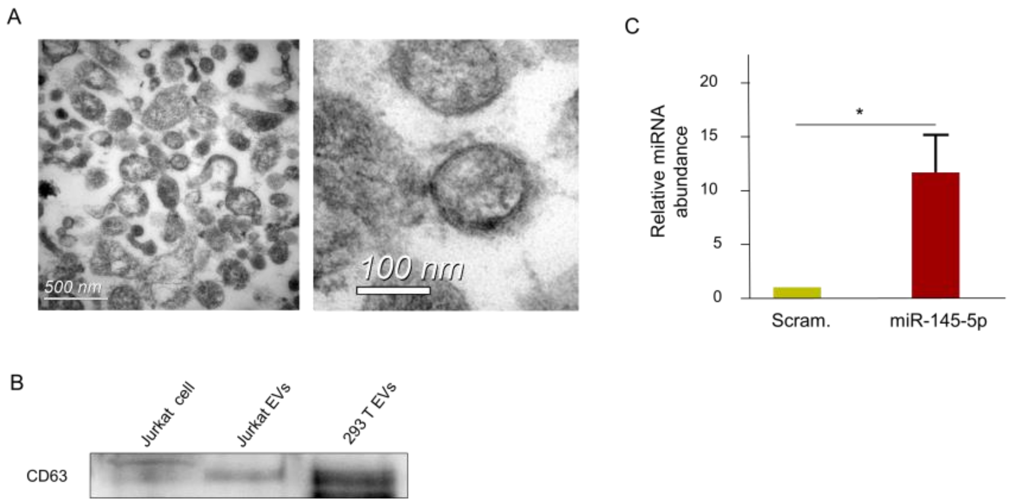

Figure S1. Characterization of miR-145-5p EM. A. EM micrographs showing morphology of the EVs. B. Immunoblotting of EV markers, CD63, which was positive for the EV samples. C. qPCR quantifications of miR-145-5p in the scramble EV and miR-145-5p EV samples (n=3), indicating that the miR-145-5p EVs had abundant miR-145-5p. * p<0.05, statistically significant.

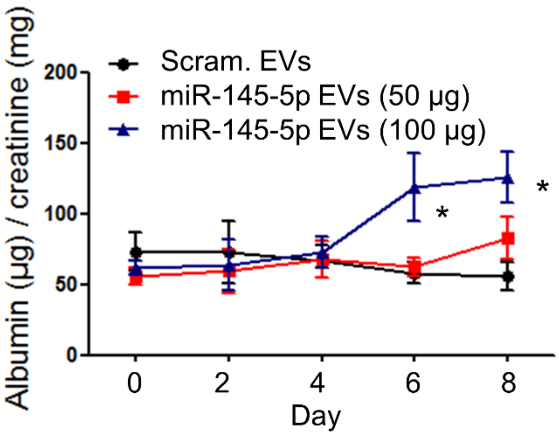

Figure S2. miR-145-5p enriched EVs induced albuminuria in healthy mice. miR-145-5p EVs were administered intravenously to mice each other day for a total of 6 days. But 100 µg dose (determined by EV-protein) of miR-145-5p EVs for every injection induced albuminuria in control mice. Data are represented as mean ±SD. *P<0.05, miR-145-5p EVs (100 µg) compared with Scram. EVs.

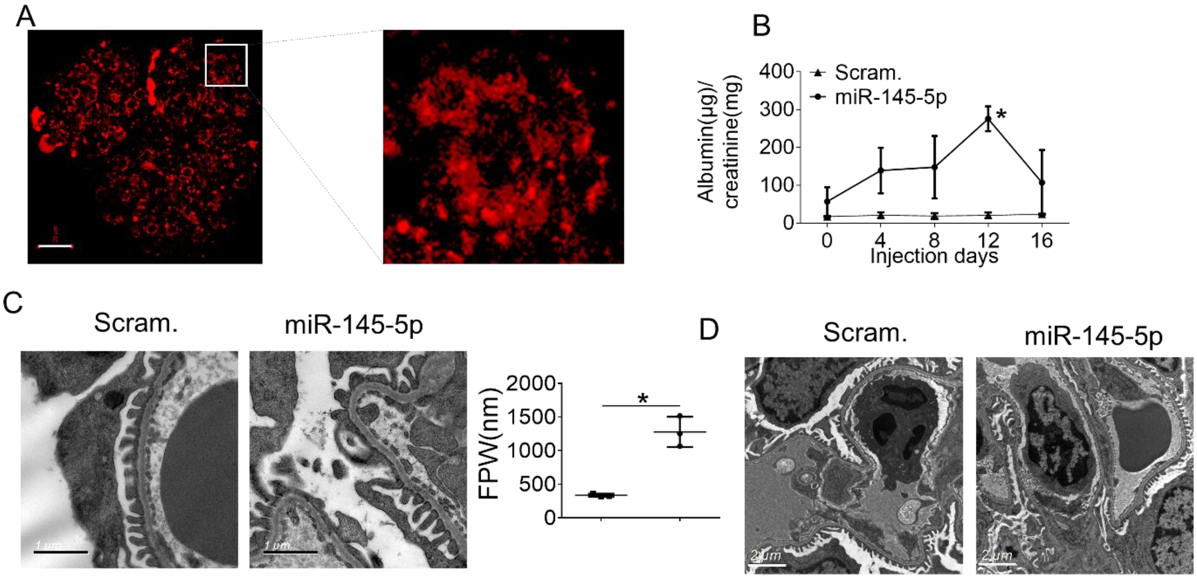

Figure S3. The in vivo effect of miR-145-5p agomir on mouse podocytes. A. Confocal microscopic imaging of a glomerulus from a mouse injected with Cy5-labeled scramble agomir via tail vein, which shows that Cy5-labeled scramble agomir was present in glomerular cells. Note that the cells surrounding capillary lumen (mainly podocytes) were heavily stained. Scale bar: 20 μm. B. Time course of albuminuria (ACR) after miR-145-5p agomir was injected through tail vein into the mice (n=3). C. EM showing podocyte foot process effacement in the mice in B. Scale bar: 1 μm. *P <0.05, statistically significant. D. EM revealed overt foot process effacement of podocytes in the mice to which miR-145-5p agomir was locally injected to the kidney. Scale bar: 2 μm.

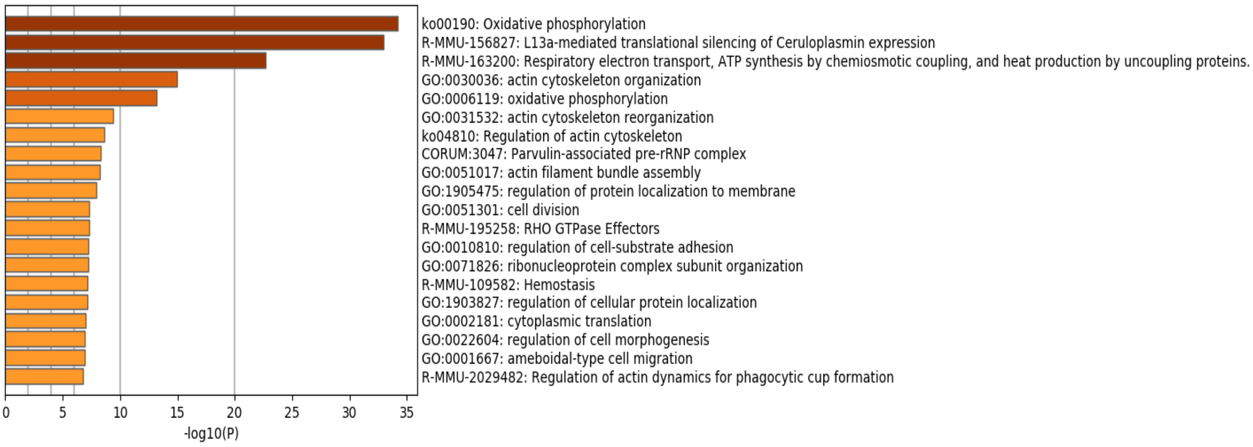

Figure S4. GO analysis of the predicted 611 podocyte essential genes.

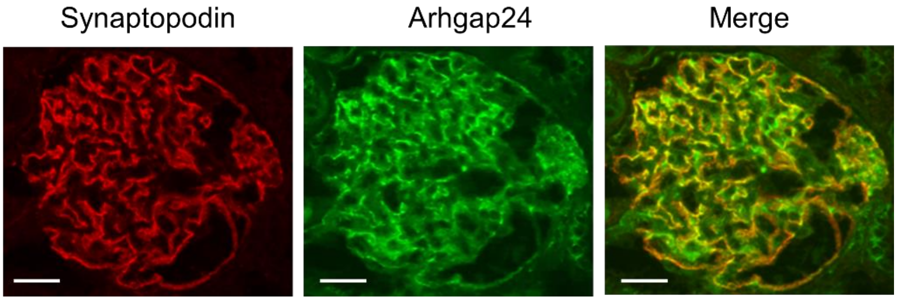

Figure S5. Immunofluorescent staining of Arhgap24 proteins in glomeruli, showing that it was colocalized with podocyte marker, synaptopodin. Scale bar: 20 μm.

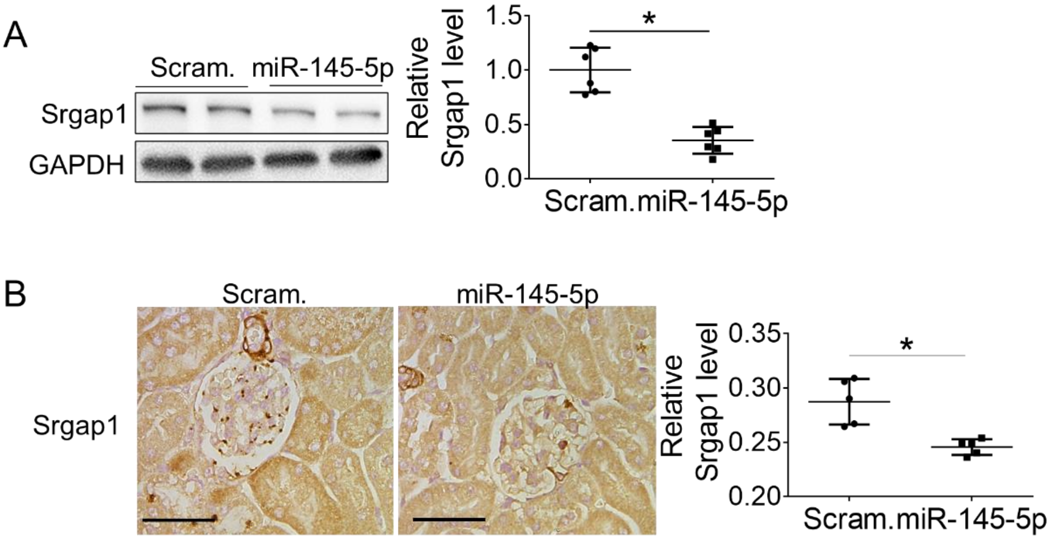

Figure S6. miR-145-5p induced Srgap1 protein loss in cultured human podocytes (A) and mice glomerular podocytes (B, n=5 mice in each group). *P<0.05. Data are represented as Mean ±SD. Scale bar, 40 μm.

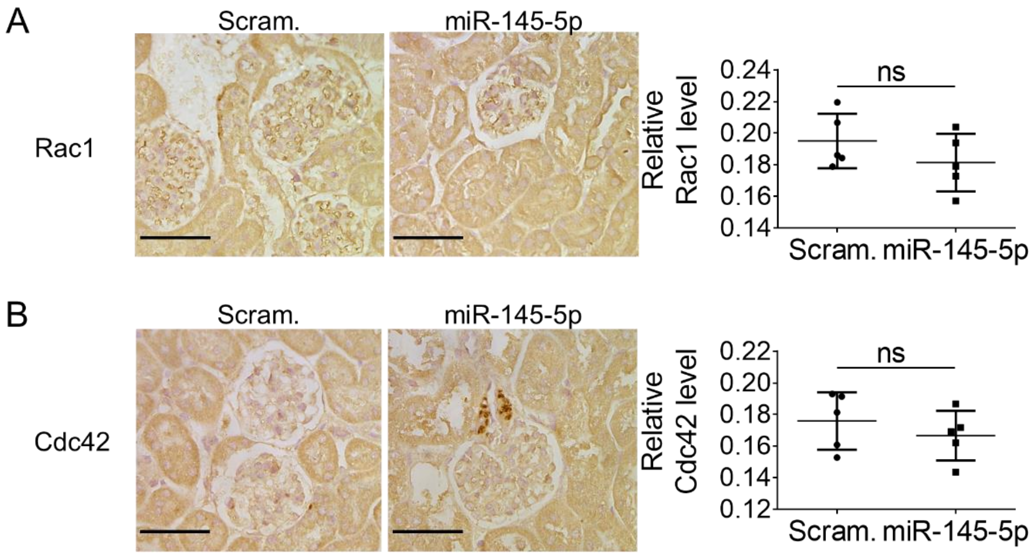

Figure S7. miR-145-5p didn’t change glomerular Rac1 (A) and Cdc42 (B) total protein levels in mice. n=5 mice in each group. ns, not significant. Data are represented as Mean ±SD. Scale bar, 40 μm.

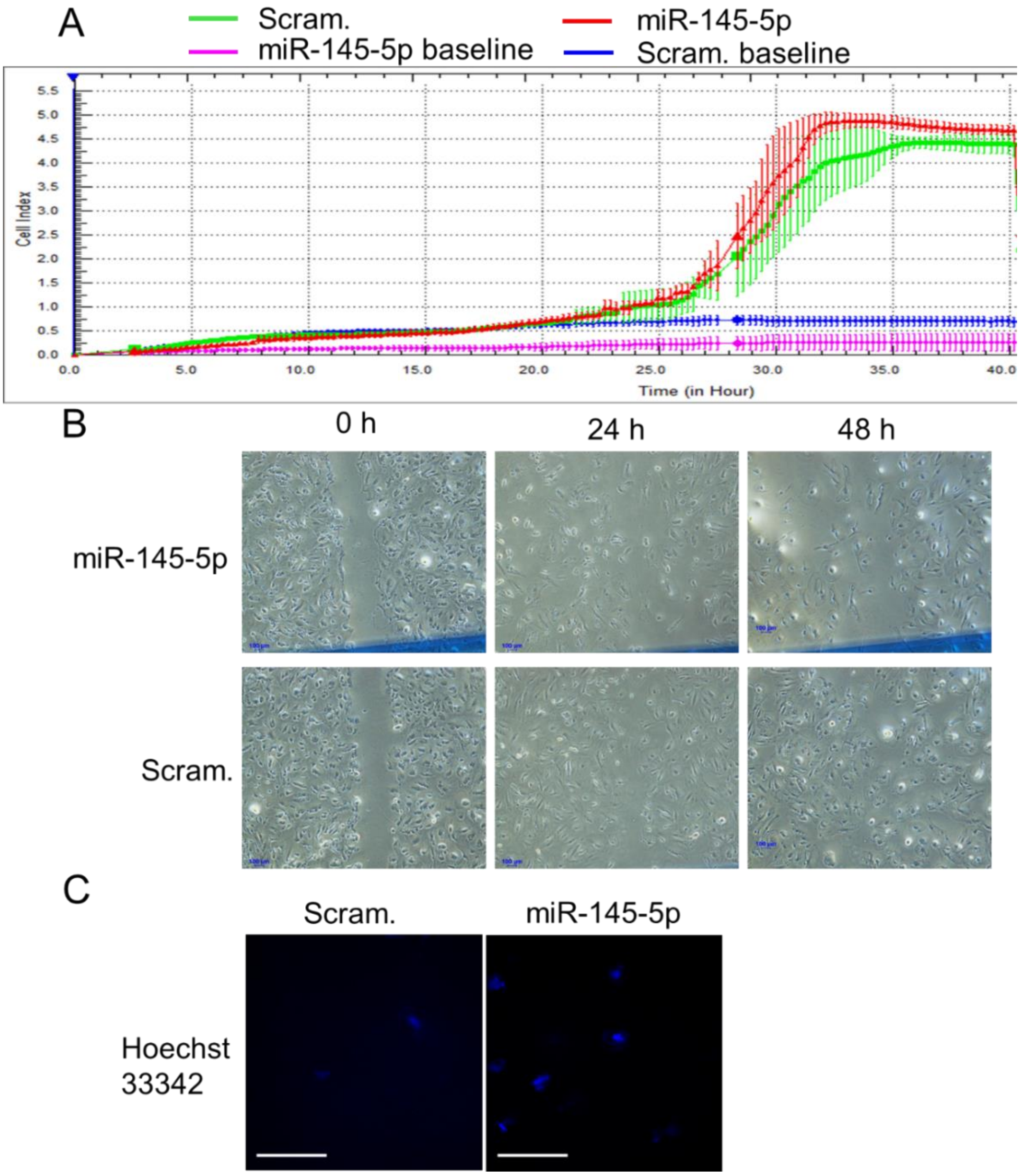

Figure S8. miR-145-5p did not affect migration but induced apoptosis of podocytes in culture. A. RTCA assay of cultured podocytes that were transfected with miR-145-5p mimic and scramble, respectively, showing no any difference between the cells treated as indicated. B. Wound healing assay showing there was no difference in scratch healing between miR-145-5p- and scramble-treated podocytes. C. Hoechst 33342 staining of the podocytes treated as indicated shows nuclear condensation that indicated the cells undergoing apoptosis. Scale bar: 100 μm.

References

1. Saleem, MA, O'Hare, MJ, Reiser, J, Coward, RJ, Inward, CD, Farren, T, *et al.* (2002). A conditionally immortalized human podocyte cell line demonstrating nephrin and podocin expression. *J Am Soc Nephrol* **13**: 630-638.

2. Bruno, S, Grange, C, Deregibus, MC, Calogero, RA, Saviozzi, S, Collino, F, *et al.* (2009). Mesenchymal Stem Cell-Derived Microvesicles Protect Against Acute Tubular Injury. *Journal of the American Society of Nephrology* **20**: 1053-1067.

3. Clotilde Th´ery, AC, Sebastian Amigorena, and Grac¸a Raposo (2006). Isolation and Characterization of Exosomes from Cell Culture Supernatants and Biological Fluids. *Current Protocols in Cell Biology*: 3.22.21-23.22.29.

4. Fu, J, Wei, C, Lee, K, Zhang, W, He, W, Chuang, P, *et al.* (2016). Comparison of Glomerular and Podocyte mRNA Profiles in Streptozotocin-Induced Diabetes. *J Am Soc Nephrol* **27**: 1006-1014.

5. van den berg, JG, van den Bergh Weerman, MA, Assmann, KJM, Weening, JJ, and Florquin, S (2004). Podocyte foot process effacement is not correlated with the level of proteinuria in human glomerulopathies. *Kidney International* **66**: 1901-1906.

6. Wu, J, Zheng, C, Wang, X, Yun, S, Zhao, Y, Liu, L, *et al.* (2015). MicroRNA-30 family members regulate calcium/calcineurin signaling in podocytes. *J Clin Invest* **125**: 4091-4106.
